# High-Yielding Flow Synthesis of a Macrocyclic Molecular Hinge

**DOI:** 10.1021/jacs.1c02891

**Published:** 2021-05-07

**Authors:** Christopher D. Jones, Laurence J. Kershaw Cook, David Marquez-Gamez, Konstantin V. Luzyanin, Jonathan W. Steed, Anna G. Slater

**Affiliations:** † Department of Chemistry and Materials Innovation Factory, University of Liverpool, Crown Street, Liverpool L69 7ZD, U.K.; ‡ Department of Chemistry, Durham University, South Road, Durham DH1 3LE, U.K.

## Abstract

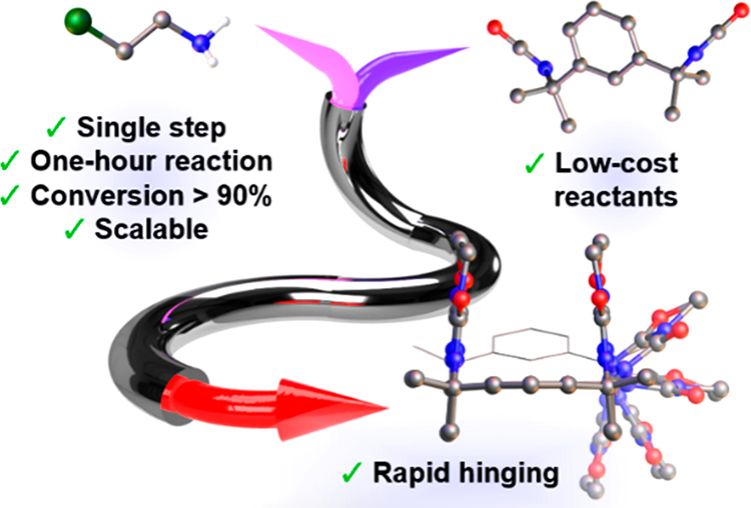

Many molecular machines are built from modular components with well-defined motile capabilities, such as axles and wheels. Hinges are particularly useful, as they provide the minimum flexibility needed for a simple and pronounced conformational change. Compounds with multiple stable conformers are common, but molecular hinges almost exclusively operate via dihedral rotations rather than truly hinge-like clamping mechanisms. An ideal molecular hinge would better reproduce the behavior of hinged devices, such as gates and tweezers, while remaining soluble, scalable, and synthetically versatile. Herein, we describe two isomeric macrocycles with clamp-like open and closed geometries, which crystallize as separate polymorphs but interconvert freely in solution. An unusual one-pot addition cyclization reaction was used to produce the macrocycles on a multigram scale from inexpensive reagents, without supramolecular templating or high-dilution conditions. Using mechanistic information from NMR kinetic studies and at-line mass spectrometry, we developed a semicontinuous flow synthesis with maximum conversions of 85–93% and over 80% selectivity for a single isomer. The macrocycles feature voids that are sterically protected from guests, including reactive species such as fluoride ions, and could therefore serve as chemically inert hinges for adaptive supramolecular receptors and flexible porous materials.

## Introduction

Biological processes depend strongly on the ability of molecules to undergo reliable and reversible changes in shape. Finely controlled conformational transitions play important roles, for example, in muscle contraction, transmembrane ion transport, and ATP synthesis.^1−3^ A key ambition of supramolecular chemists is to engineer molecular machines capable of performing useful work, such as catalysis, transport, and host–guest binding, with comparable precision.^4,5^ To date, synthetic molecular machines have featured complex arrangements of moving parts, including rings that shuttle between stations on a linear or circular track^6,7^ and crane-like arms that can transfer labile moieties between reactive docking sites.^8,9^

Molecular machines typically consist of modular components linked by covalent or mechanical bonds. Each component must deliver reversible conformational changes in a simple and predictable fashion. For example, a nanocar may be constructed by connecting pseudospherical adamantane or fullerene wheels to a central dipolar chassis via an alkyne axle.^10^ Rotors^11^ and shuttles^7^ commonly incorporate a catenane or rotaxane, while hinges are built from moieties with interconvertible geometric isomers. Suitable structures include photoisomerizable double bonds, such as stilbenes, imines, and azobenzenes,^12^ and fused aliphatic rings with distinct *chair*, *boat*, and *skew* conformations.^13,14^ Hinging provides a mechanism for controlling resonance energy transfer^15^ and other physical phenomena dependent on the separation of interacting groups. Alternatively, hinged architectures may function as molecular clips or tweezers,^16,17^ varying the distance between their closing “jaws” to maximize binding with an encapsulated guest.^18−20^

**Figure 1:**
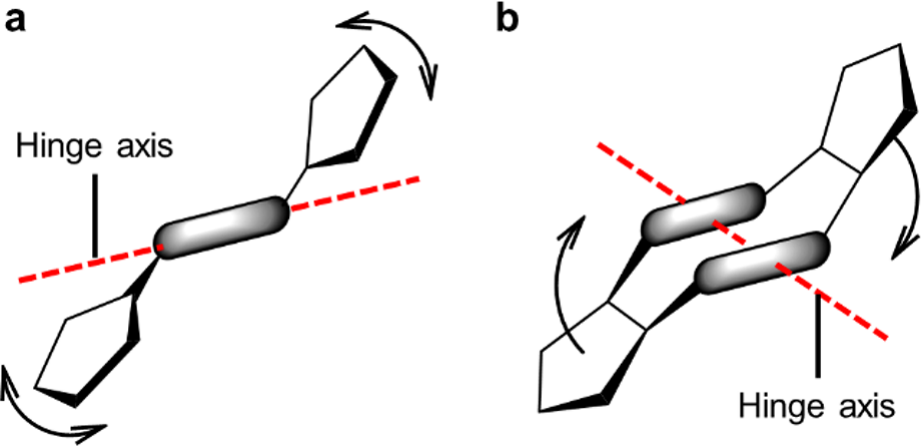
(a) Schematic mechanism of a typical molecular hinge based on dihedral rotations around a rigid spacer, such as a double bond or disubstituted ring. (b) Clamp-like mechanism of a macrocyclic molecular hinge (this work).

Despite their simple mechanical function, synthesizing molecular hinges with widespread utility remains a challenge. Hinges based on a flexible single bond,^21−23^ double bond,^15,24−26^ or disubstituted ring system^27−30^ operate through dihedral rotations, analogous to the twisting of a crank around a shaft. Because they cannot undergo a clamping motion, such hinges are suboptimal scaffolds for pincer-like receptors (Figure 1a). In addition, isomerization of these structures is often triggered by inputs such as heat, light, or redox reactions.^31^ The need for a stimulus is disadvantageous in applications such as adaptive host–guest binding, where switching must occur rapidly and reversibly under ambient conditions.^32^

A truly versatile molecular hinge must function in a range of chemical environments without disrupting target reactions and supramolecular motifs. Thus, hinges should be soluble, simple to synthesize, chemically inert, and incapable of significant host–guest interactions. To ensure that hinging is predictable and entropically feasible,^33^ the process should involve well-defined open and closed structures with no alternative stable conformations. Unfortunately, synthetic scaffolds displaying suitable conformational isomerism^34−36^ are often insoluble, reactive, or difficult to prepare, making them inconvenient building blocks for modular functional materials.^37^

Macrocycles represent a promising starting point for the construction of more reliable clamp-like molecular hinges (Figure 1b). Even complex macrocycles may be highly rigid, exhibiting a precise arrangement of functional groups around a well-defined central cavity.^38^ Thus, conformational isomerism in macrocycles often involves a small number of structures with shapes that are easy to distinguish and usefully diverse.^39−42^ Another important advantage of macrocycles is that binding sites are confined to fixed locations within an easily modified intrinsic void.^43^ The shape of this void may be tuned to ensure tight complementarity with a target guest, for applications such as catalysis, drug delivery, and molecular recognition.^44^ Likewise, undesirable guest uptake by a macrocyclic hinge may be avoided by the inclusion of competitive intramolecular motifs or bulky peripheral substituents, which present a steric barrier to incoming species.^34^

Designing and synthesizing a flexible macrocycle can be a challenging task. In the absence of preorganized intermediates, it is often necessary to use protecting groups, supramolecular templates, and high-dilution conditions to promote macrocycle formation over oligomerization pathways.^45^ Syntheses may thus be slow and low yielding or involve multiple protection and deprotection steps. Problems of this nature are increasingly resolved through the use of flow reaction platforms,^46,47^ in combination with in- or online reaction monitoring and automated optimization techniques^48^ to identify the most efficient and selective reaction conditions. Reactants in flow may be mixed, heated, and cooled more uniformly, and the synthetic protocol can be adjusted continuously in response to real-time conversion and kinetic measurements. By enabling more controlled addition of reagents and higher reaction temperatures, flow technology has allowed macrocycles to be generated more rapidly, in higher yields and with fewer side products than conventional batch methods.^49^

**Figure 2:**
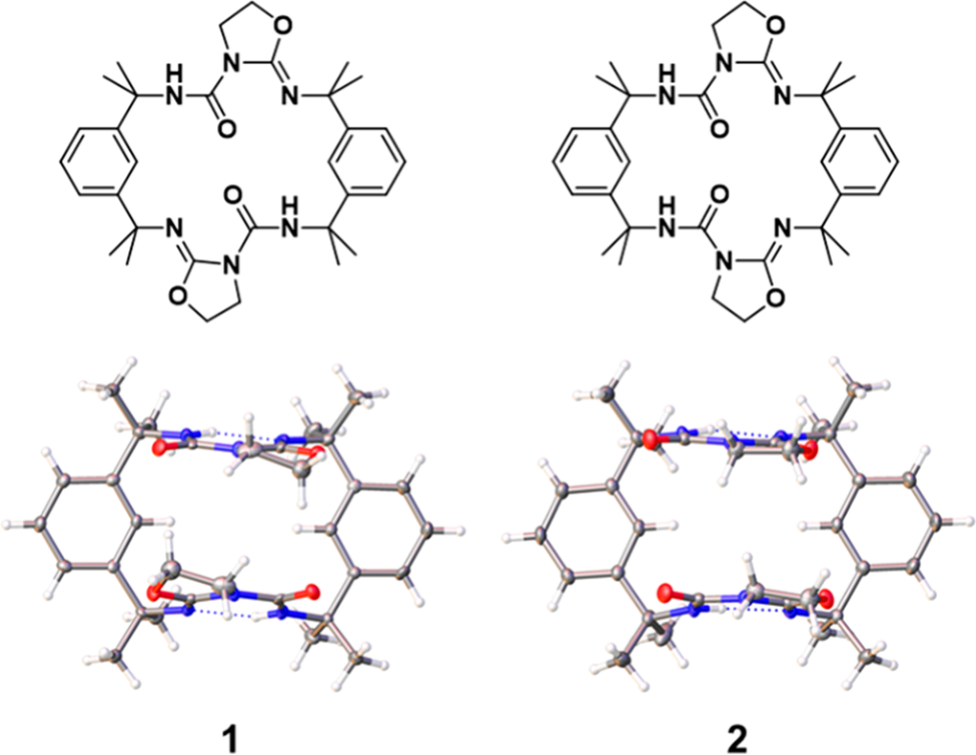
Formulas and crystal structures of isomeric macrocycles **1** and **2** in their *syn* conformations. Gray, white, blue, and red atoms in the crystal structures correspond to C, H, N, and O, respectively. Oxazolidine rings are angled out of the macrocycle plane, producing clamp-like geometries.

**Figure 3:**
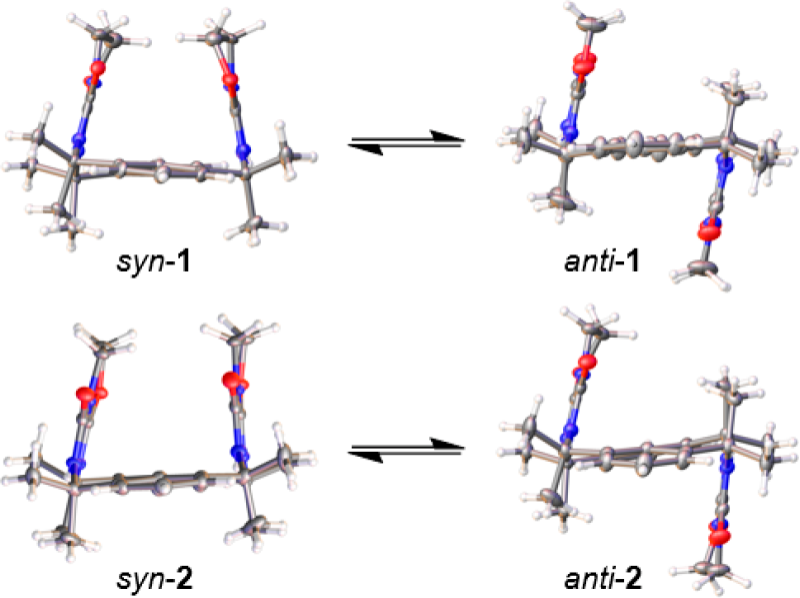
Crystal structures of **1** and **2** in their *syn* and *anti* geometries. The conformers interconvert readily in solution but crystallize separately as concomitant single crystals.

In this investigation, two isomeric macrocycles, **1** and **2** (Figure 2), were prepared from readily available reagents via a one-pot addition–cyclization reaction. Remarkably, each macrocycle transitions between a pair of distinct conformers in solution, which can be isolated as separate concomitant single crystals for analysis by single-crystal X-ray diffraction (SCXRD) (Figure 3). Furthermore, the ratio of macrocycles in the product may be tuned by varying the sequence in which the starting materials are mixed. A semicontinuous synthetic method was used to maximize the rate of macrocycle formation and attain high selectivity for product **2**. Synthesizing the macrocycles in flow allows the intermediates and products to be monitored over a range of temperatures, aiding optimization of the reaction conditions and kinetic analysis of key mechanistic steps.

As molecular hinges, macrocycles **1** and **2** display several useful properties. First, *syn*–*anti* transitions offer access to well-defined open and closed geometries, which can be readily characterized in both solution and the solid state. On the basis of variable-temperature NMR (VT-NMR), crystallographic, and computational studies, we propose that the hinging mechanism differs from that of similarly flexible macrocycles, such as calixpyrroles,^50^ in that it involves a simple rapid clamping motion with no alternative conformers. In addition, NMR and computational studies suggest that the macrocycles interact only weakly with guests, including protic solvents and highly basic species such as fluoride ions, due to shielding of their internal voids by bulky methyl groups and oxazolidine rings. Finally, both compounds are readily soluble and may be synthesized easily and inexpensively on a large scale. Given the simplicity and derivatizability of the amine^51^ and isocyanate^52^ reactants, these structures would serve as useful scaffolds for clamp-like receptors, hinged molecular machines, and porous framework materials, enabling reliable changes in shape and adaptive recognition of target guests.

## Results and Discussion

### Synthesis and Characterization

**Figure 4:**
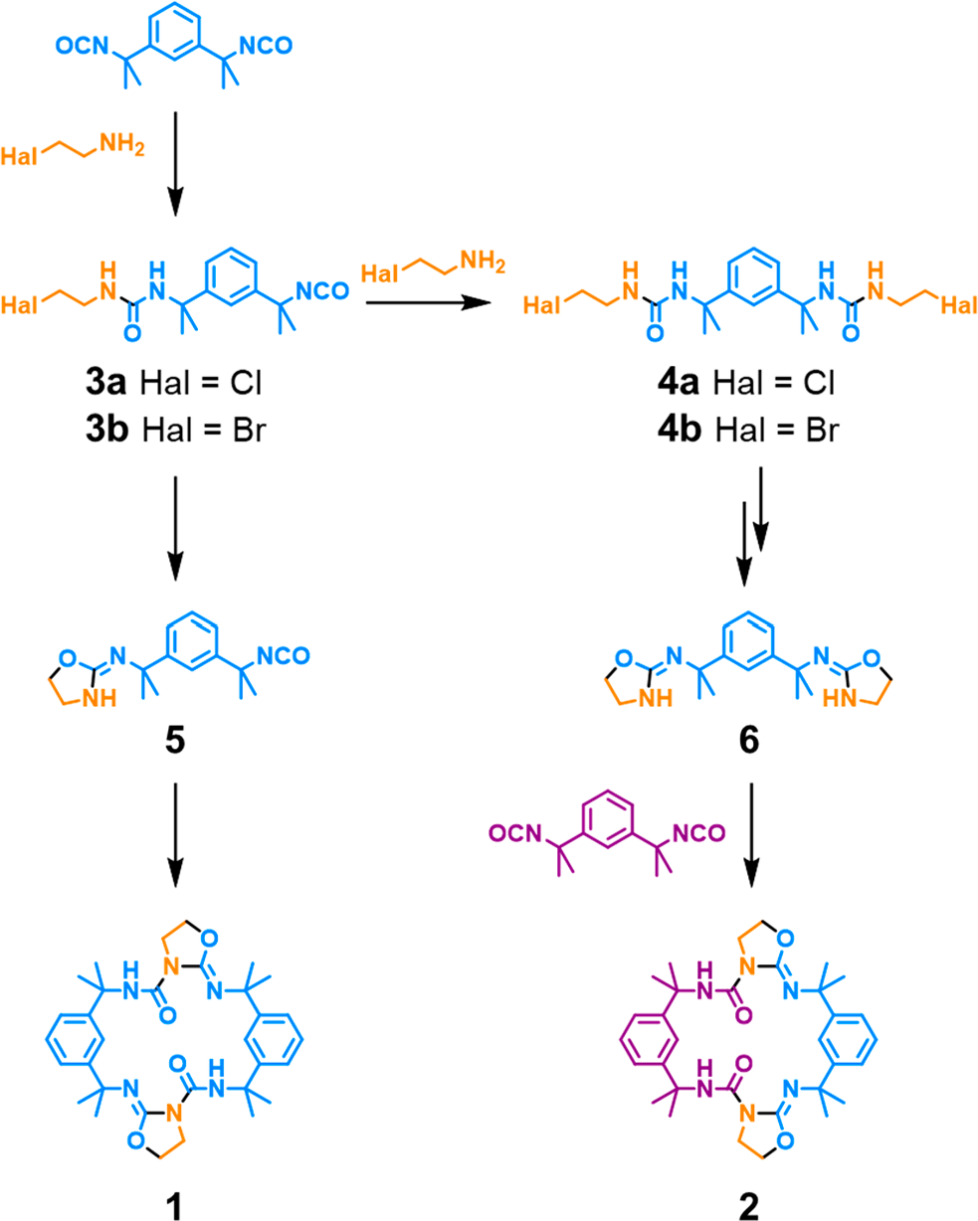
Proposed mechanism for the one-pot synthesis of macrocycles **1** and **2** via the unusual oxazolidine intermediates **5** and **6**. Formulas are colored to highlight the origins of atoms and bonds in the final products, while newly formed bonds are indicated in black. Reactions are performed in chloroform at 20–60 °C with 300 mM of triethylamine, 145 mM of the amine hydrohalide, and 1 equiv of the diisocyanate added in one or two stages.

A mixture of **1** and **2** is generated by reacting tetramethylxylylene diisocyanate with 2-chloroethylamine or 2-bromoethylamine in the presence of triethylamine. In our proposed mechanism (Figure 4), the reaction generates a monourea **3** or bis-urea **4**, which slowly cyclize to form the nucleophilic 2-iminooxazolidines **5** and **6**. These intermediates likely exist as a mixture of tautomers and stereoisomers^53^ but react further to form macrocycles only in the *Z* configurations, which allow for a stabilizing intramolecular hydrogen bond in the final product.^54^ Macrocycle **1** is produced by the homocoupling of **5**, while its isomer **2** results from the addition of **6** to a second equivalent of diisocyanate. The structures of the oxazolidine intermediates and their mechanism of formation are highly unusual. Although cyanate ions can undergo cyclization reactions with substituted alkylamines,^55^ comparable O-alkylation of an isocyanate is rarely reported. Indeed, to the best of our knowledge, this type of reaction has been described only once before^56^ and has never been exploited for macrocycle synthesis.

Macrocycles **1** and **2** are insoluble in water but dissolve readily in chloroform and dichloromethane and more sparingly in the polar solvents methanol and dimethyl sulfoxide. The compounds can be separated by column chromatography and purified by recrystallization from methanol. The structures of the compounds were confirmed by SCXRD (Figures S17–S20 and Table S1) and are consistent with elemental analysis, mass spectrometry, and NMR spectroscopy data (Figures S1–S12).

Intriguingly, each compound adopts two stable conformations that crystallize separately as concomitant polymorphs. The conformers differ in the orientations of the oxazolidine rings, displaying either U-shaped *syn* or Z-shaped *anti* configurations (Figure 3). Isomers *anti*-**1** and *syn*-**2** are achiral, while the chiral compounds *syn*-**1** and *anti*-**2** give rise to intrinsically racemic crystal forms. The conformers are rigidified by intramolecular hydrogen bonds between the urea and imine groups^57^ but otherwise exhibit no significant supramolecular motifs in the solid state. The lack of strong interactions between macrocycles likely accounts for their high solubility in certain nonpolar solvents. This solution processability allows the stereodynamic and host–guest binding properties of the compounds to be readily assessed and could simplify their large-scale synthesis and functionalization for practical applications.

### Molecular Hinge Behavior

**Figure 5:**
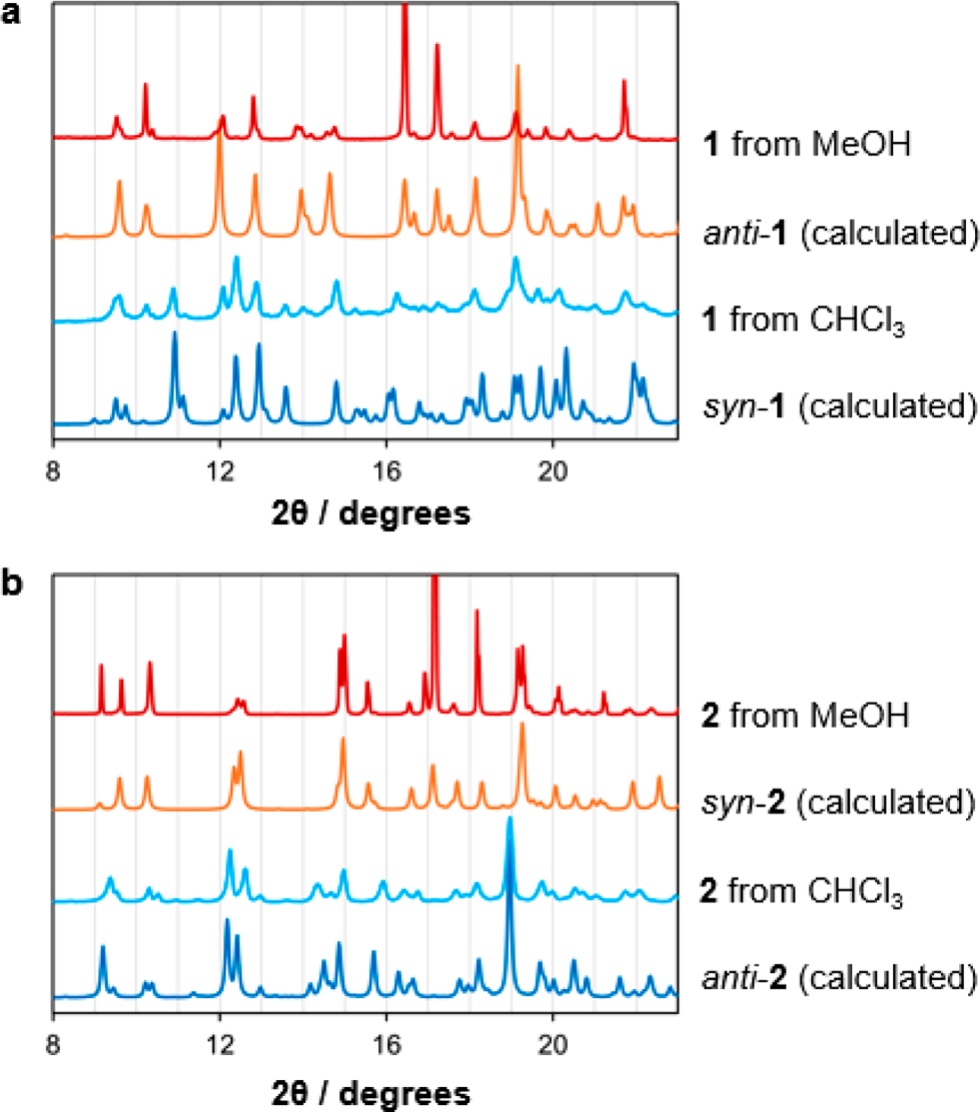
Calculated PXRD patterns of (a) **1** and (b) **2** from the crystal structures of their *syn* and *anti* conformers and the experimental patterns obtained after recrystallization of the compounds from chloroform and methanol.

The conformational isomerism of compounds **1** and **2** is unusually well-defined. Each macrocycle exhibits distinct *syn* and *anti* forms, which can be readily distinguished by powder X-ray diffraction (PXRD). When 10 mM chloroform solutions of the compounds are slowly evaporated, the resulting precipitates consist mainly of the *syn*-**1** (Figure 5a) and *anti*-**2** (Figure 5b) polymorphs. However, recrystallization of the materials from methanol causes the *anti***-1** and *syn***-2** polymorphs to preferentially form. The ^1^H NMR spectrum of each macrocycle at room temperature contains one set of resonances that can be assigned to the thermal average of its *syn* and *anti* conformations, with no indication of separate atropisomeric^58^ species (Figures S1 and S7). We conclude that interconversion of the conformers is not fully restricted by the bulky methyl groups of **1** and **2** and occurs rapidly in solution on the NMR time scale, as in the case of the similarly methylated calixpyrroles.^50^ This switching behavior allows the ratio of conformers to vary during crystallization, favoring different polymorphs depending on the crystal growth conditions.

**Figure 6:**
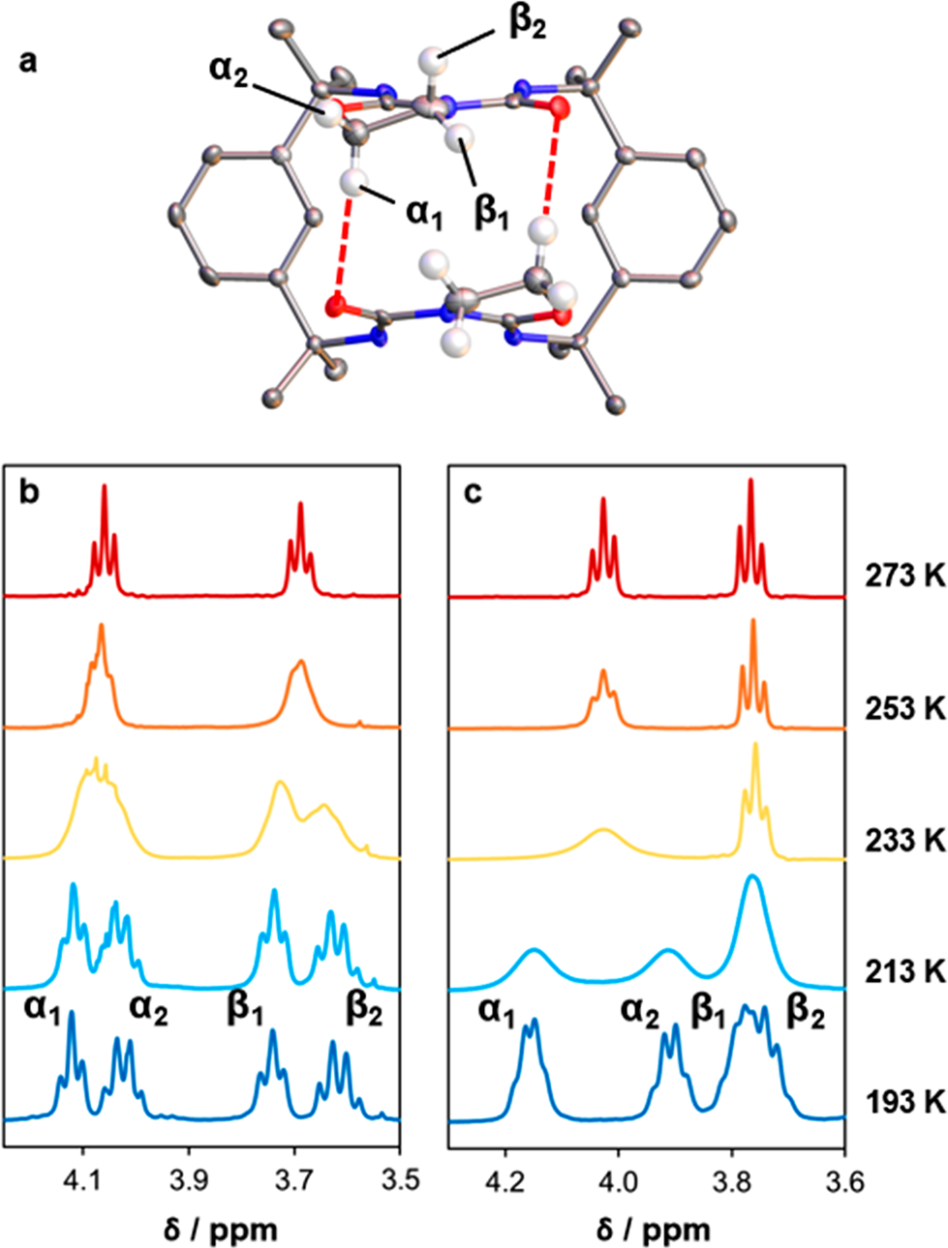
(a) Assignment of the four methylene proton environments in **1**, with close CH···OC contacts illustrated in red. The methylene protons are enlarged and other protons omitted for clarity. (b) VT-NMR spectra of **1**, showing splitting of the ^1^H methylene signals below 253 K. (c) VT-NMR spectra of **2**, showing splitting of the ^1^H methylene signals below 233 K.

Variable-temperature ^1^H NMR (VT-NMR) offers further insight into the mechanism of conformational switching (Figures S22–S24 and Table S3).^59^ Each macrocycle displays two triplet signals in the range 3.5–4.3 ppm that were attributed to the four methylene protons of the oxazolidine ring, matching the NMR assignments of literature analogues (Figure 6a).^54^ At room temperature, the protons of each CH_2_ site are chemically and magnetically equivalent due to rapid interconversion of the *syn* and *anti* conformers. However, when dichloromethane-*d*_2_ solutions of **1** (Figure 6b) and **2** (Figure 6c) are cooled below −20 and −40 °C, respectively, the inequivalent protons α_1_ and α_2_ and β_1_ and β_2_ are clearly resolved as separate with matching integrals. We propose that protons α_1_ and β_1_ give rise to downfield signals due to interactions with the opposing oxazolidine rings and, in macrocycle **1**, the attached urea carbonyl groups. Indeed, the crystal structure of *syn*-**1** displays interatomic CH···OC distances of 2.6–2.9 Å (with a mean value of 2.73 Å), while CH···HC contacts in both *syn*-**1** and *syn*-**2** lie in the range 2.3–2.8 Å (with means of 2.49 and 2.57 Å for **1** and **2**, respectively).

The coalescence temperature, *T*_c_, for each methylene group was estimated by extrapolating the chemical shifts of the split signals to the point of convergence. This value was used to estimate the activation energy for switching, Δ*G*^‡^, via the Eyring equation:^59^
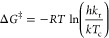
1where *k* is the Boltzmann constant, *h* the Planck constant, *R* the molar gas constant, and *k*_r_ the rate constant for the conformational change. The value of *k*_r_ is determined from Δδ, the maximum separation of the *syn* and *anti* signals in hertz:
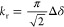
2For **1** and **2**, this analysis produces Δ*G*^‡^ values of 54 ± 1 and 47 ± 1 kJ mol^–1^, respectively. Macrocycle **1** displays a slightly larger barrier for the *syn*–*anti* transition, suggesting that opening of the *syn* form is resisted by stronger interactions between the oxazolidine rings.

To rationalize their relative stabilities, the *syn* and *anti* conformers of **1** and **2** were modeled by using the density functional theory (DFT) method B3LYP^60^ in the basis sets 6-31++G**,^61^ def2-TZVP,^62^ and aug-cc-pVDZ (Table S4).^63^ After geometry optimization, *syn*-**1** is ∼13 kJ mol^–1^ lower in energy than *anti*-**1**, while *anti-***2** is 6 kJ mol^–1^ more stable than *syn*-**2**. The oxazolidine rings of *syn*-**1** interact more strongly due to the antiparallel alignment of dipoles and close contacts between the methylene and carbonyl groups. In *syn*-**2**, where the oxazolidine rings exhibit a parallel configuration, no such CH···OC interactions are possible. Thus, *syn*-**1** and *anti*-**2** are expected to be the dominant conformers of the macrocycles in solution. This hypothesis is supported by our PXRD studies, which indicate a greater abundance of the predicted low-energy conformers when **1** and **2** are precipitated from chloroform (Figure 5). Preferential crystallization of the higher-energy *anti*-**1** and *syn*-**2** conformers from methanol could result from solvent–macrocycle hydrogen bonding or other stabilizing solvation effects, which are not accounted for in our DFT calculations.

**Figure 7:**
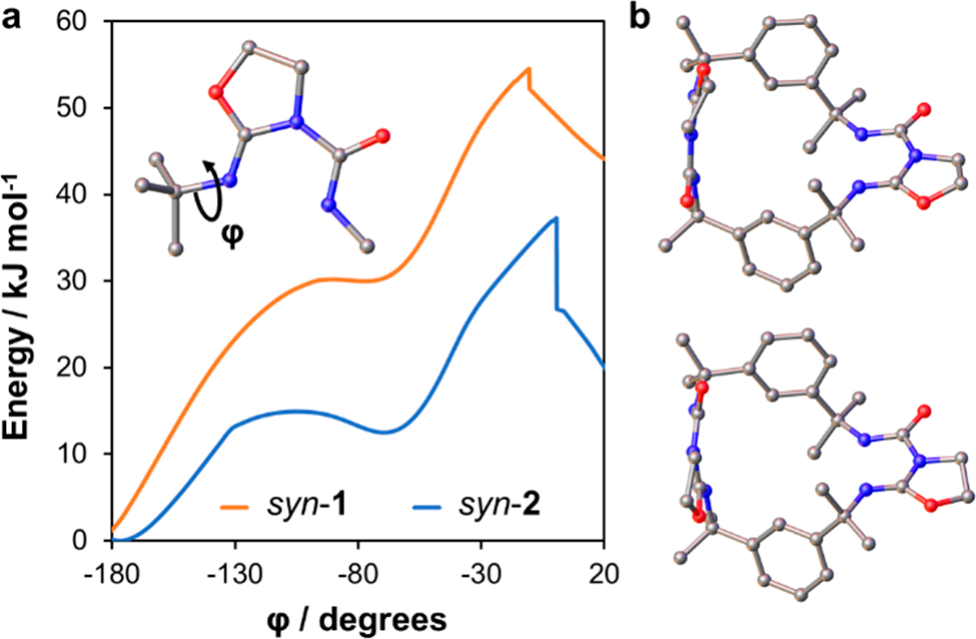
(a) DFT energies (B3LYP/6-31+G*) of **1** and **2** for varying values of the methylimine torsion angle (inset). (b) Highest energy conformations of **1** (top) and **2** (bottom), in which the oxazolidine rings are approximately perpendicular and the phenyl groups twist out of the macrocycle plane.

Additional DFT modeling was undertaken to explore the mechanism of conformational switching (Figures S25–S29 and Table S5). For each conformer, one torsion angle was altered in increments of 0.2°–5.0° until the alternative macrocycle structure was reached. The geometry was optimized for each fixed torsion angle in the 6-31+G* basis set and its energy compared with that of the starting conformation (Figure 7a). The calculations suggest that the two conformational changes are mechanistically similar, involving rotations of the phenyl groups out of the plane of the macrocycle (Figure 7b). The intramolecular hydrogen bonds are strongly preserved, forcing each urea–oxazolidine motif to move as a single rigid structure like the jaw of a clamp. Following refinement of the highest energy geometries in a range of larger basis sets, we estimated an activation barrier of 37–41 kJ mol^–1^ for conversion of *anti*-**2** to the less stable *syn* form. Conversion of *anti*-**1** to *syn*-**1** is opposed by a similar energy barrier, but the reverse transition displays a much larger activation energy of 51–54 kJ mol^–1^ due to the relatively high stability of the *syn* geometry.

Strong agreement between these results and those of our VT-NMR experiments suggests that the behavior of the macrocycles in solution has been accurately described. Interestingly, further refining the model with a D3BJ dispersion correction^64^ does not significantly alter the mechanisms of the conformational transitions or the energy barrier for macrocycle **2** (Figures S27–S29 and Tables S4 and S5). However, the correction increases the stability of *syn*-**1**, and thus the barrier for the *syn*-*anti* transition, by 16–19 kJ mol^–1^. This discrepancy could be due to an overestimation of dispersion forces between the oxazolidine rings or the omission of solvation effects from the modeled system.

It should be noted that VT-NMR experiments require low temperatures to be reached without precipitation of the solute or freezing of the solvent. In this investigation, dichloromethane-*d*_2_ was selected because it dissolves the macrocycles readily at the required temperatures and displays a lower freezing point (−96.7 °C) than chloroform-*d* (−63.5 °C). More polar media such as methanol-*d*_4_ are unsuitable due to the sparing solubility of the macrocycles in these solvents. Nonetheless, supramolecular effects on the hinging process could be usefully explored by repeating the VT-NMR experiments in solvent mixtures or in the presence of cosolutes such as organic acids and halide salts. Such comparative studies will be attempted as part of future investigations.

Given the simplicity and rapidity of their conformational switching behavior, compounds **1** and **2** represent appealing scaffolds for the construction of larger clamp-like macrocycles. There is much scope for derivatizing the xylylene spacers or decorating the oxazolidine rings with functional substituents, like the rim substituents of calixarenes^35^ and calixpyrroles,^36^ to deliver improved aqueous solubility, stronger host–guest binding, or useful stimuli-responsive properties.^65^ In addition, the ability to incorporate a reliable hinge into more complex materials, such as polymers and framework solids, could enable better control of characteristics such as porosity and gelation capacity. Exploring this modular synthetic approach is a key objective of our ongoing research.

### Batch Synthesis

**Figure 8:**
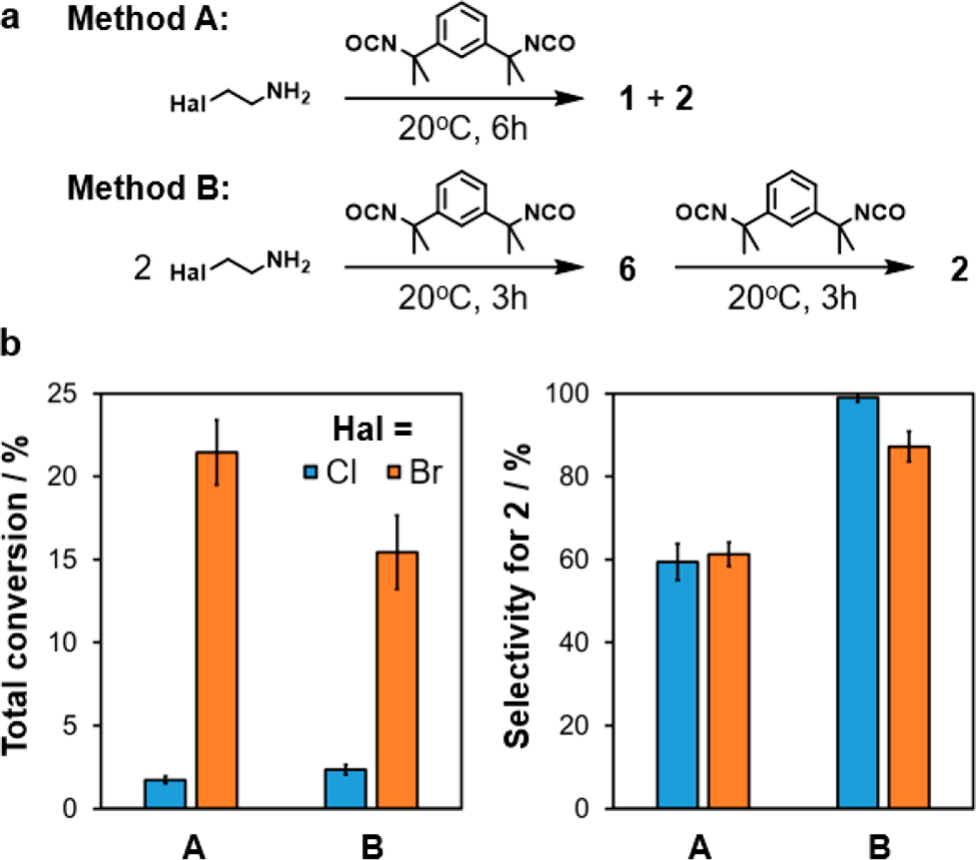
(a) One-pot macrocyclization reactions involving one (method A) or two (method B) reagent addition steps. (b) Total conversions and selectivities of macrocycle syntheses by using different methodologies and amine reactants. Selectivity is equal to the amount of **2** produced as a percentage of the total conversion. Error bars display the standard deviations for replicate trials (*n* = 4).

Macrocycles **1** and **2** can be produced in different ratios by varying the sequence in which reagents are mixed (Figure 8a). If the amine and isocyanate react in an equimolar ratio over 6 h (method A), neither isomer is strongly favored. By contrast, adding two equivalents of the amine to the neat isocyanate followed by a second equivalent of neat isocyanate after 3 h (method B) results in a high selectivity for compound **2**. It is proposed that the ureas **3** and **4** form rapidly but only slowly cyclize to yield the oxazolidines **5** and **6**. Thus, method B allows for near-quantitative conversion of mono-urea **3** to bis-urea **4**, preventing the formation of **1** via intermediate **5**.

The selectivity of the reaction can be reliably measured without purification due to the presence of highly deshielded urea protons in the macrocycle structures. The NMR signals for the urea groups of **1** and **2** occur as singlets at 10.8 and 10.6 ppm, respectively, making them easily distinguishable from other species. Conversions can also be determined by NMR analysis. After quenching and evaporating the reaction mixture, the residue is dissolved in CDCl_3_, and the integrals of the urea signals are normalized by using acetonitrile as an internal standard.

The outcome of the reactions strongly depends on the halide leaving group of the amine reactant (Figure 8b, Figure S32, and Table S7). Use of 2-chloroethylamine produces low conversions of 1.7 ± 0.2% in method A and 2.3 ± 0.3% in method B but enables a selectivity of nearly 100% in the latter process. Reactions with 2-bromoethylamine occur more readily, delivering conversions of 21 ± 2% and 15 ± 2% for methods A and B, respectively. However, the selectivity of method B for **2** is compromised when this starting material is used, reaching a value of just 87 ± 4%. The poorer leaving group of 2-chloroethylamine results in a higher selectivity and lower conversion because the oxazolidine rings are produced more slowly, ensuring that urea formation is complete before macrocyclization occurs.

In both methods A and B, higher temperatures and longer reaction times enable total isolated macrocycle yields of 60–80%. Furthermore, the selectivity of method B for isomer **2** exceeds 90% even if both steps occur at 50 °C with a total reaction time of 24 h. Higher reaction rates are possible if the synthesis is performed as a semicontinuous process (*vide infra*). Nonetheless, as one-pot processes involving inexpensive and readily available starting materials, the batch reactions offer a viable route for the large-scale manufacture of macrocyclic hinges. We anticipate reactions of this type finding widespread use in the synthesis of more complex molecular architectures, generating flexible structures with unusual three-dimensional morphologies in a single reaction step. Efforts to derivatize macrocycles for this purpose are currently underway.

### Mechanistic Studies

**Figure 9:**
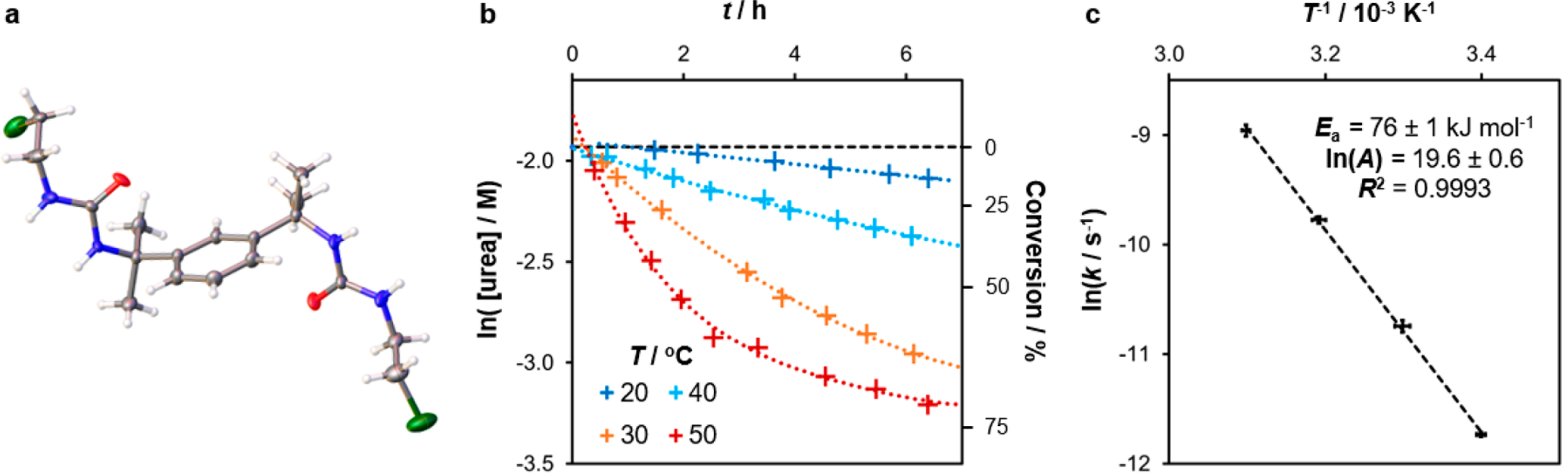
(a) Crystal structure of **4a** in its orthorhombic polymorph, obtained by slow evaporation of an acetonitrile solution. (b) First-order kinetic plot comparing urea conversions over time (*t*) at different reaction temperatures (*T*), with a dashed line marking the maximum urea concentration. (c) Arrhenius plot for the urea cyclization reaction, from which the activation energy (*E*_a_) and pre-exponential factor (*A*) may be calculated. Error bars represent the standard error in ln(*k*) and the standard deviation in *T*^–1^ for replicate experiments (*n* = 4).

The high selectivity of reactions involving 2-chloroethylamine suggests that conversion of the bis-urea **4a** to oxazolidine **6a** is a rate-determining step at room temperature. Indeed, by performing the first step of method B at 0 °C and evaporating the reaction mixture after 1 h, the intermediate **4a** was obtained in a 45% yield (Figures S13–S16). Like other bis-ureas derived from tetramethylxylylene diisocyanate and an alkylamine,^66^**4a** can be recrystallized from polar solvents to yield single crystals suitable for analysis by SCXRD (Figure 9a). Molecules of the bis-urea adopt an extended conformation and interact via urea–urea tape motifs, crystallizing from acetonitrile as a three-dimensional hydrogen-bonding network of hydrogen-bonded tapes and from methanol as a lamellar structure (Figure S21 and Table S2). Intriguingly, NMR analysis of the compound in DMSO-*d*_6_ indicates that cyclization may be induced by heating, without risk of oligomerization (Figure S33). Thus, it may be possible to generate oxazolidine **6** in a more controlled fashion for incorporation into asymmetric macrocyclic species.

To gain further insight into the rate-determining reaction step, formation of the urea and oxazolidine intermediates was monitored at room temperature by *in situ* NMR spectroscopy (Figures S35–S38 and Tables S8–S10). Because of strong overlap of their NMR signals, urea intermediates such as **3** and **4** cannot be quantified separately. Nonetheless, monitoring the disappearance of urea signals in the range 5.5–6.5 ppm allows the average cyclization rate constant to be measured (Figure 9b). The two amine starting materials react with the isocyanate at similarly high rates, delivering maximum urea concentrations within 15 min. However, the intermediates produced from 2-chloroethylamine, **3a** and **4a**, cyclize 87 ± 3 times more slowly. The cyclization displays pseudo-first-order kinetics, with a rate constant *k* of (7.0 ± 0.1) × 10^–4^ s^–1^ for 2-bromoethylamine and just (8.0 ± 0.2) × 10^–6^ s^–1^ for 2-chloroethylamine at 21 °C. The half-life of **3a** and **4a** is 24.0 ± 0.5 h, while **3b** and **4b** display a half-life of 16.5 ± 0.3 min.

Selective formation of macrocycle **2** is possible only if the initial amine-isocyanate addition reaction is considerably faster than the cyclization step. Once the unwanted intermediate **3a** has been consumed, cyclization may be safely accelerated to optimize the rate of macrocycle formation. We assessed the thermal dependency of cyclization in method B by performing NMR kinetic studies at several temperatures and estimating the activation energy *E*_a_ from an Arrhenius plot (Figure 9c). The results reveal an *E*_a_ value of 76.5 ± 1.4 kJ mol^–1^ and frequency factor^67^ ln *A* of (4 ± 2) × 10^8^ s^–1^, meaning that a reaction temperature of 70 ± 1 °C is needed to match the room-temperature *k* value of 2-bromoethylamine. Because the boiling point of chloroform at standard pressure is only 61 °C, heating a batch reaction mixture is unlikely to produce the optimum cyclization rate.

**Figure 10:**
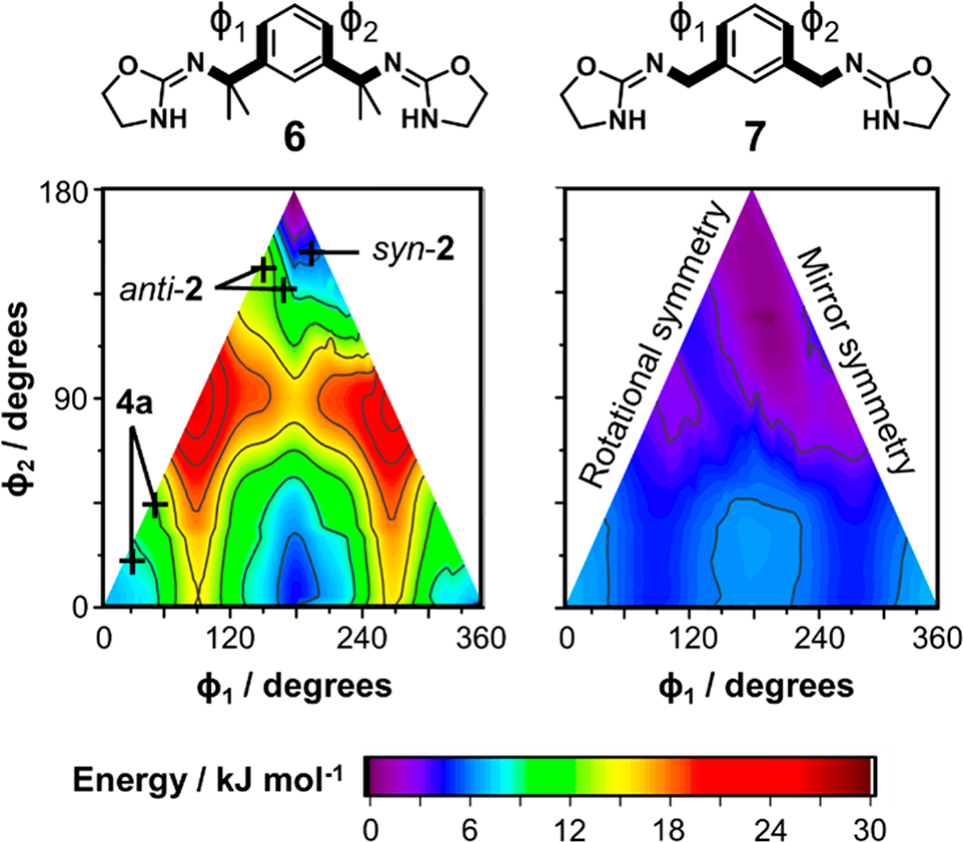
DFT energies (B3LYP/6-31+G*) of bis-oxazolidine **6** and its theoretical analogue **7** for 5° increments of the torsion angles φ_1_ and φ_2_. The contour plots are calculated by averaging the results of replicate geometry optimizations (*n* = 8) with different initial molecular conformations. Crystal structure geometries of bis-urea **4a** and macrocycle **2** are plotted for comparison.

In the final stage of the reaction, macrocyclization is favored over oligomerization due to preorganization of the reaction intermediates. It is likely that **6** adopts a C-shaped conformation similar to the rigid geometries of the bis-oxazoline (BOX) and related “boxman” compounds, which are used as chelating ligands for asymmetric catalysis.^68,69^ The methyl groups of the xylylene spacer are highly important, as reactions using the non-methylated isocyanate as a starting material produce insoluble ureas with no significant macrocycle formation (Figure S34). To rationalize this observation, DFT energies were calculated for varying conformations of **6** and its non-methylated analogue **7**, spanning all possible xylylene–oxazolidine torsion angles φ_1_ and φ_2_ (Figure 10). Because of the symmetry of the molecules, the energies are recorded on triangular contour plots, and convergence may be assessed by comparing symmetry-equivalent combinations of φ_1_ and φ_2_ (Figure S30 and Table S6). Optimizations were performed with a D3BJ correction for dispersion forces, but omitting this adjustment was found to have little effect on the final appearance of the contour plot (Figure S31).

The energy landscapes of the intermediates reveal large differences in molecular flexibility. Compound **7** can adopt many conformations with a maximum difference in energy of just 9.5 kJ mol^–1^. By contrast, conformations of **6** differ by up to 25 kJ mol^–1^ and are most stable when the phenyl ring and C–N bonds are approximately coplanar, as in macrocycles **1** and **2**. We conclude that methylation of the xylylene group lowers the entropic cost of macrocycle formation and increases the abundance of suitable precursor geometries.^70^ This conformational bias, which is comparable to the Thorpe–Ingold effect,^71^ usefully eliminates the need for templating or high-dilution conditions typically encountered in macrocycle syntheses.^45^

### Flow Synthesis

Selective production of **2** allows the macrocycle to be isolated without wasteful separation steps. However, the reliability of the synthesis is limited by the need for stepwise addition of the isocyanate. Furthermore, the reaction is inconveniently slow and could be challenging to scale up, as effective mixing is needed to maintain a constant stoichiometric ratio of the starting materials. While increasing the temperature can improve the efficiency of the process, excess heating may lower selectivity by enabling early accumulation of intermediate **5**. This problem could be minimized by completing each step of the synthesis at a different temperature. However, it is difficult to make rapid changes to the conditions of a batch reaction while ensuring uniformity of the reaction mixture, particularly if the process is conducted on a larger scale.

**Figure 11:**
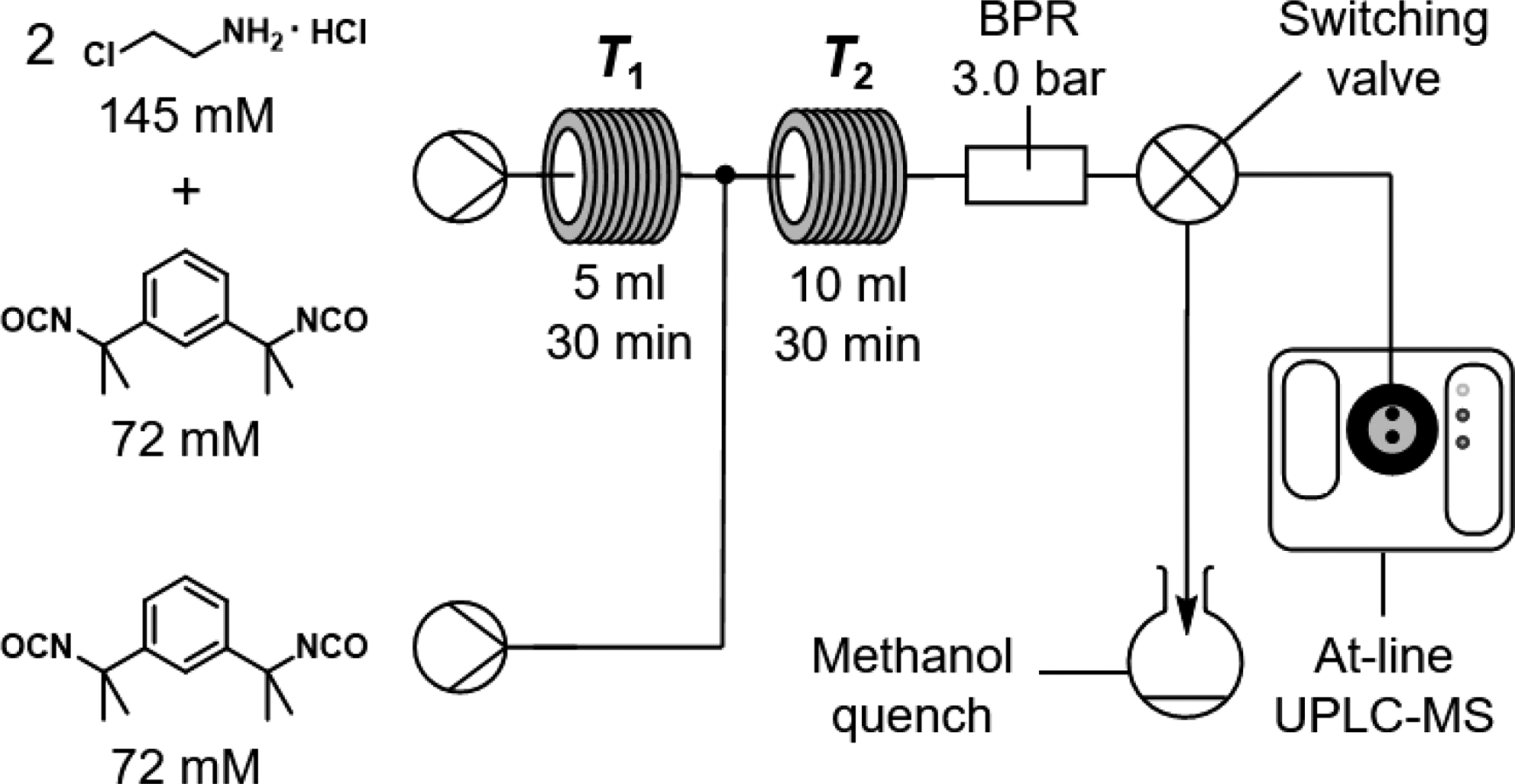
Flow reactor schematic for the semicontinuous synthesis of macrocycle **2**. The solutions are prepared with 300 mM triethylamine in chloroform and mixed after the first coil in a 1:1 volumetric ratio. A switching valve may be used to sample the reaction mixture before or after the second coil for at-line analysis by UPLC-MS. A methanol quench is included to allow reaction conversions to be accurately measured.

The yield, consistency, and scalability of the reaction can be enhanced by transferring the batch process to a continuous flow platform (Figure 11). By performing the synthesis in a Vaportec R-Series flow reactor with two heated coils, we were able to ensure a high mixing rate and automate reagent additions at fixed time points. Use of a dynamic back-pressure regulator (BPR) set to 3.0 bar allowed the reaction temperature to be safely increased up to 100 °C. Furthermore, a switching valve enabled automatic sampling of the reaction mixture for analysis by ultraperformance liquid chromatography mass spectrometry (UPLC-MS). These at-line measurements provided additional insight into the reaction mechanism by allowing intermediates and side products to be rapidly detected.

**Figure 12:**
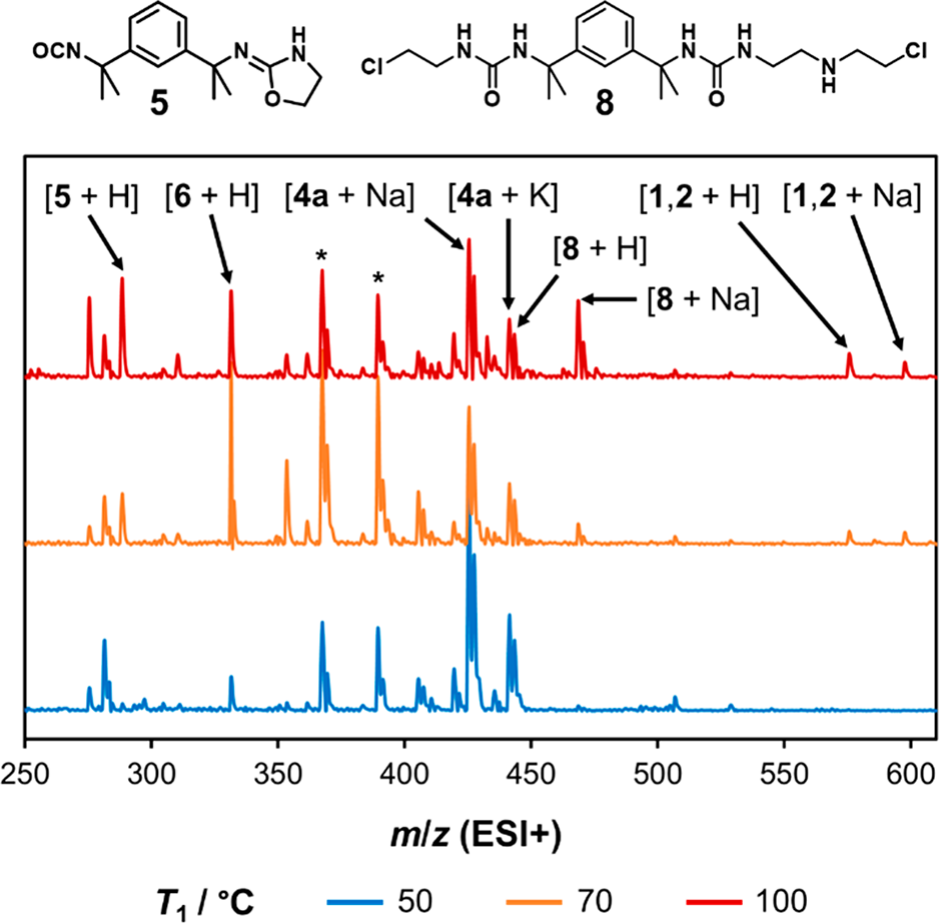
At-line mass spectra for reaction mixtures sampled after the first reaction step at different temperatures (*T*_1_). Molecular ions of the macrocycles, intermediates, and a proposed adduct of **4a** and 2-chloroethylamine are assigned. Monocyclization of **4a** produces a further intermediate, for which the protonated molecular ion (*m*/*z* 367) and sodium adduct (*m*/*z* 389) are marked with asterisks. All peaks occur at the predicted *m*/*z* values of the assigned species.

For each flow reaction, stock solutions of the starting materials were prepared by using a 300 mM solution of triethylamine in chloroform. NMR studies confirm that the compounds are stable in solution for over 24 h at room temperature (Figure S39). In the first, noncontinuous step, neat isocyanate was mixed with two equivalents of the amine as in method B. The resulting mixture was heated to temperature *T*_1_ in the first coil to drive formation of the oxazolidine species. Finally, the reaction was completed by mixing the solution with another equivalent of isocyanate in a second coil at temperature *T*_2_. The selectivity of the synthesis for product **2** depends on minimizing macrocycle formation in the first reaction step. Indeed, in less selective reactions, UPLC-MS measurements after the first coil (Figure 12) reveal positive ion signals for the protonated macrocycle (*m*/*z* 575) and its sodium adduct (*m*/*z* 597). A signal at *m*/*z* 288 is also observed after both coils, corresponding to the unwanted intermediate **5** in the first step and a fragment ion of **1** in the final product mixture (Figure S40).

Products of the flow reactions were quenched immediately in methanol and quantified against an acetonitrile standard as in the batch reaction studies (Figure S41, Tables S11 and S13). Repeat experiments confirm the reliability of the protocol, demonstrating that replicate conversions and selectivities typically vary by less than 2 percentage points (Tables S12 and S14). The impact of the first heated coil was assessed by varying *T*_1_ and fixing *T*_2_ at a low value of 50 °C to minimize oxazolidine formation in the second coil. Flow syntheses at *T*_1_ > 60 °C are higher yielding than the batch reactions of both 2-chloro- and 2-bromoethylamine at room temperature, despite being performed over shorter times and with higher dilutions in the macrocyclization step. This result suggests that oxazolidine formation is not rate limiting when *T*_1_ is high, allowing changes in *T*_2_ to strongly influence the macrocycle yield.

**Figure 13:**
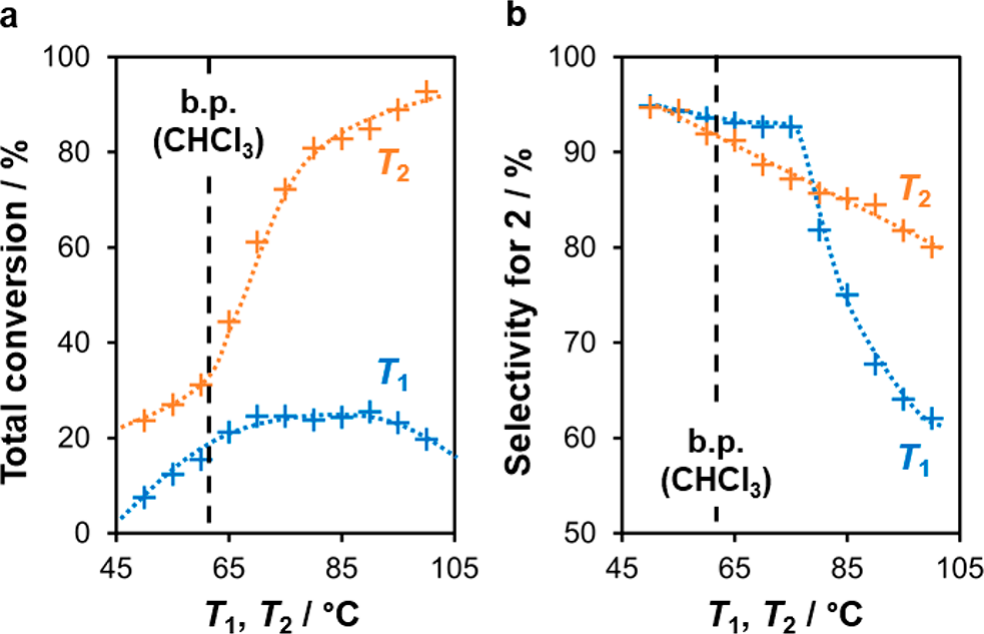
(a) Total conversions and (b) selectivities for reactions performed at different reaction temperatures *T*_1_ and *T*_2_. Values of *T*_1_ are compared at *T*_2_ = 50 °C, while values of *T*_2_ are compared at *T*_1_ = 70 °C. Where replicate experiments were performed, mean values are reported. Trend lines are drawn as guides for the eye only. The boiling point of chloroform is marked to indicate the maximum temperature of comparable batch reactions.

As *T*_1_ is increased from 50 to 75 °C, more macrocycle is generated due to greater formation of intermediate **6** (Figure 13a). However, a decrease in conversion above 90 °C is evidence of competing reaction pathways, which inhibit the cyclization process. Likewise, the selectivity for product **2** remains above 90% when *T*_1_ < 75 °C but falls sharply at higher temperatures (Figure 13b). UPLC-MS analysis after the first coil at *T*_1_ = 100 °C reveals a strong signal matching the sodium adduct of **8** (*m*/*z* 468), a bis-urea derived from the reaction of **4a** with 2-chloroethylamine (Figure 12). It is concluded that excess heating in the first coil promotes off-target amine alkylation reactions, preventing the later formation of macrocyclic products.

The final reaction step was optimized by varying *T*_2_ at a fixed value of *T*_1_ = 70 °C. Although increasing *T*_2_ also lowers the selectivity for **2**, more complete formation of intermediate **6** in the first coil limits the potential for alkyl adducts and other side products. Thus, selectivity varies more gradually with temperature than in the first coil, decreasing by just 3 percentage points per 10 °C of heating. Conversion, however, rises steeply with *T*_2_, reaching 85–93% between 90 and 100 °C with a maximum 70–74% yield of **2**. The maximum macrocycle production rate for our system is 0.25 g/h under steady-state conditions. All major NMR signals of the crude products at high *T*_2_ can be assigned to **1**, **2**, and triethylamine, confirming that the reaction approaches completion while avoiding off-target reaction pathways (Figure S42).

The optimized values of *T*_1_ and *T*_2_ exceed the boiling point of the chloroform solvent (61 °C). Thus, the high-yielding synthesis of **2** is only possible due to pressurization of the system in flow. Indeed, batch and flow syntheses conducted at 60 °C with equal reaction times produce similar total conversions of just 28 ± 1 and 27 ± 2%, respectively, with selectivities of 97.8 ± 0.5 and 95.5 ± 0.5% (Figure S43 and Table S15). We conclude that the use of a flow reactor allows yields to be more than tripled by providing safe and reliable access to higher reaction temperatures.

### Host–Guest Binding

**Figure 14:**
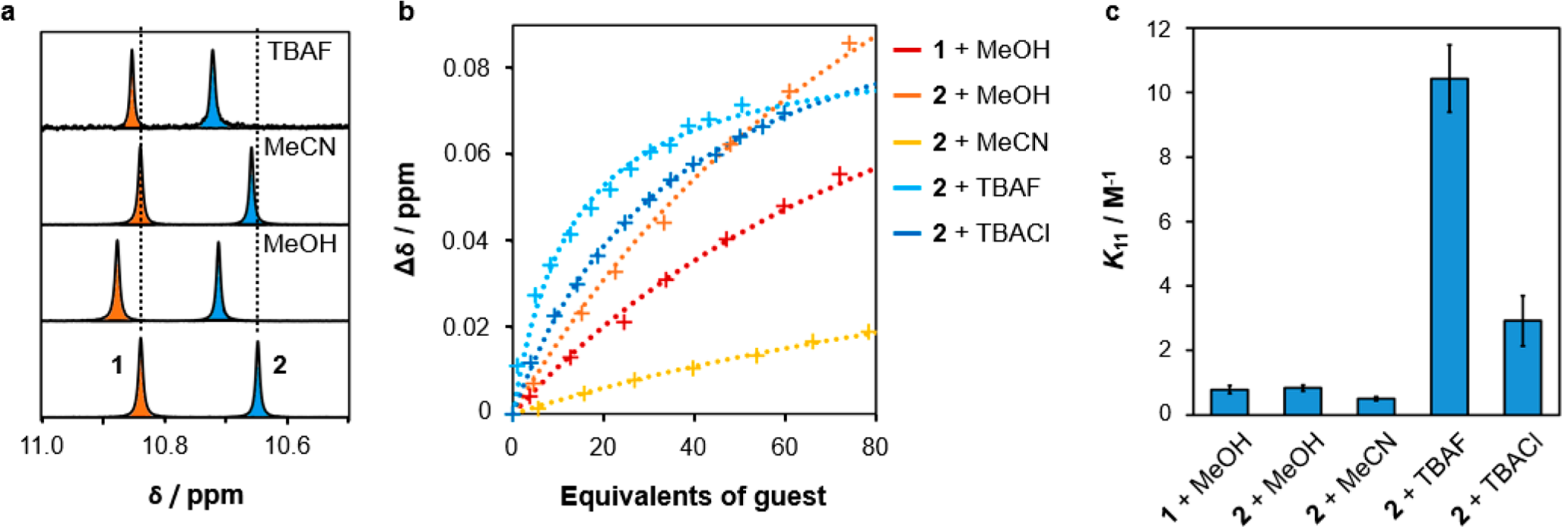
(a) ^1^H NMR signals of the NH groups of **1** (marked in orange) and **2** (blue), before and after the addition of different guests (50 equiv). Spectra were recorded by using separate 9 mM solutions of **1** and **2** in CDCl_3_ and concatenated to aid comparison. (b) ^1^H NMR chemical shifts of the NH groups in **1** and **2** relative to TMS, with fixed macrocycle concentrations (9 mM) and varying amounts of a neutral or ionic guest. Trend lines correspond to the best-fit binding isotherms for the Δδ values. (c) Mean 1:1 association constants for the macrocycle–guest combinations, with error bars representing the standard error in *K*_11_ for replicate experiments (*n* = 3).

In their *syn* conformations, macrocycles **1** and **2** are geometrically similar to molecular clips.^16,17^ These C-shaped molecules are designed to provide a rigid concave binding surface to encapsulate guests in a shape-selective fashion. Host–guest interactions could limit the usefulness of a molecular hinge, however, by impeding changes in conformation and competing with the binding sites of attached receptor moieties. To investigate this possibility, solutions of the macrocycles in CDCl_3_ were titrated with 0–120 equiv of various guests (Figure 14a, Figures S44–S46, and Table S16).^72^ Changes in the NMR chemical shift of the NH proton, Δδ, were measured relative to a reference tetramethylsilane (TMS) signal and fitted to a suitable isotherm if significant binding (Δδ > 0.02 ppm) was observed (Figure 14b).

Remarkably, neither macrocycle interacts strongly with most of the neutral guests tested. Indeed, only methanol was found to produce measurable values of Δδ for **1** as well as **2**, interacting in a 1:1 stoichiometry with an association constant (*K*_11_) of ∼0.8 M^–1^ in both cases (Figure 14c). Similarly low *K*_11_ values have been recorded for hydrogen-bonded complexes of esters,^73^ suggesting that methanol forms OH···OC hydrogen bonds but does not bind directly to the NH groups. Macrocycle **2** also displays small but significant Δδ values with hydrogen bond acceptors such as acetonitrile, acetone, and dimethylformamide. Titration studies with acetonitrile reveal a *K*_11_ value smaller than that of methanol, consistent with the formation of weaker CH···nitrile and CH···carbonyl interactions. We hypothesize that acetonitrile and other small polar guests can bind loosely to **2** by entering the narrow void of the macrocycle with only slight disruption of its stable conformation.

The mechanisms of binding were explored further by modeling the interactions of methanol, acetonitrile, acetone, and chloroform with the *syn* and *anti* conformers of both macrocycles (Figures S49 and S50, Table S17). DFT optimizations were performed from a variety of starting configurations in the 6-31+G* basis set and then refined in the larger basis set 6-31+G**. Counterpoise corrections for basis set superposition error^74^ were omitted due to their negligible impact on energy values (Table S18). The energy of each host–guest interaction (*E*_int_) was calculated by subtracting the total energy of the free host and guest from the energy of the geometry-optimized complex.^74^ Finally, the favorability of the structures was estimated by comparing their *E*_int_ values with those of the corresponding chloroform complexes. Although *E*_int_ does not account for guest–guest interactions or changes in solvation, it nonetheless offers insight into the relative binding strengths of **1** and **2** and the structural differences between their host–guest assemblies.

**Figure 15:**
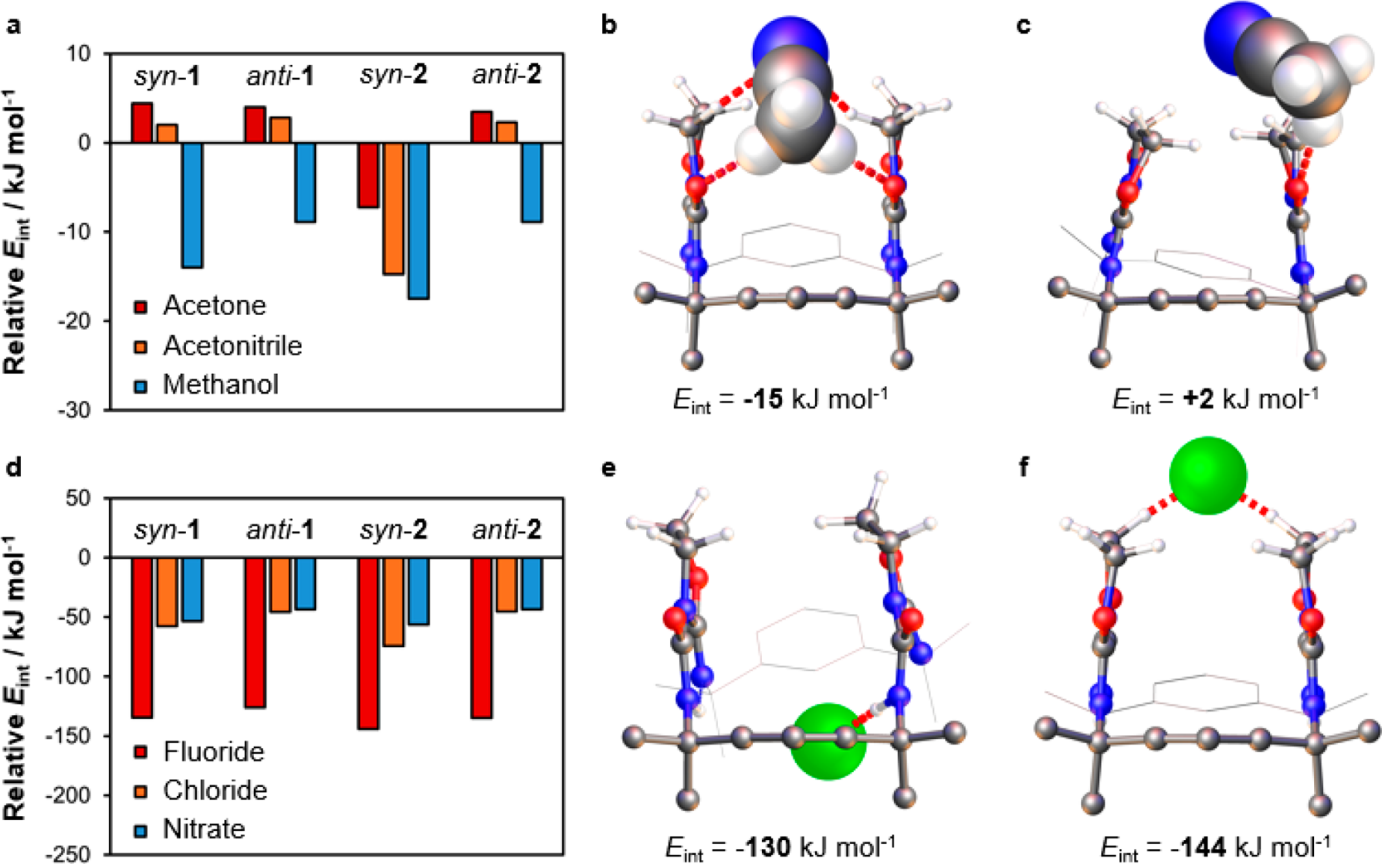
(a) Calculated (B3LYP/6-31++G**) interaction energies (*E*_int_) for complexes of **1** and **2** with neutral guests and the most stable binding complexes of acetonitrile with (b) **2** and (c) **1**. (d) *E*_int_ values for complexes of **1** and **2** with anionic guests and the most stable (e) NH···F^–^ and (f) CH···F^–^ contacts in fluoride complexes of **2**. All *E*_int_ values are expressed relative to the corresponding chloroform complexes. Hydrogen bonds and major dipole–dipole interactions are marked in red, and parts of the macrocycles are omitted for clarity.

As predicted, the DFT results suggest that all macrocycle conformers engage in methanol–carbonyl hydrogen bonding without undergoing significant deformation. However, only *syn*-**2** can interact effectively with acetone and acetonitrile (Figure 15a), establishing multiple interactions with the guests via the oxazolidine methylene and urea carbonyl groups (Figure 15b). Chloroform associates less strongly as it is unable to establish the same bifurcated dipole–dipole motifs. Likewise, **1** displays smaller *E*_int_ values because guests cannot interact simultaneously with both carbonyl groups (Figure 15c). In the *syn*-**1** conformer, binding is further weakened by the presence of CH···OC contacts, which compete with the formation of intermolecular hydrogen bonds and prevent separation of the oxazolidine rings.

Titration of the macrocycles with anionic species, in the form of tetrabutylammonium (TBA) salts, also produces measurable Δδ values. For **1**, these changes are too small for the association constants to be reliably quantified. Conversely, **2** interacts with fluoride and chloride to give Δδ values in the range 0.06–0.07 ppm. The smaller Δδ values of other salts indicate relatively weak binding to the TBA cation, while comparisons of hydrated and anhydrous TBACl suggest that interactions with water of crystallization are similarly minor (Figure S47). In addition, the absence of a triplet peak in the region 15–17 ppm confirms that macrocycles are not deprotonated by fluoride to form bifluoride (HF_2_^–^) ions (Figure S48).^75^ Both halides conform to a 1:1 binding model and interact more strongly than the neutral guests. However, the *K*_11_ values of the anions are smaller than those of typical NH-halide complexes by 3–4 orders of magnitude.^76^ For 90% of the molecules of **2** to participate in 1:1 binding, at least 0.88 ± 0.09 M (98 ± 10 equiv) TBAF or 3.3 ± 0.9 M (370 ± 100 equiv) TBACl would be required. It is likely that halide–macrocycle interactions are destabilized by competing intramolecular interactions or heavily disfavored by the compact macrocycle geometry.

DFT modeling (Figure S15d) suggests that the binding of anions by **2** is controlled by steric crowding around the NH sites. In both the *syn* and *anti* conformers, chloride and nitrate ions are too large to fit within the macrocycle void so interact primarily with external CH groups (Figure S51). The fluoride ion exhibits a larger *E*_int_ value and can penetrate further between the methyl groups and oxazolidine rings, even engaging in NH···F^–^ hydrogen bonding (Figure 15e). However, these interactions are weakened by the resulting conformational strain, making them less stable than the alternative CH···F^–^ contacts (Figure 15f). As expected from the NMR data, compound **1** binds anions consistently less strongly in both of its conformers. Though NH···F^–^ hydrogen bonds are still possible, particularly in the *anti* geometry, these offer only a small enthalpic advantage over alternative binding modes so are unlikely to be strongly favored in solution.

Neither the NMR nor the DFT studies assess the impact of solvent on host–guest binding. However, our analysis suggests that hydrogen bond donors such as methanol affect the relative stabilities of the macrocycle conformers, in agreement with the outcomes of crystallization trials. In combination with VT-NMR experiments, host–guest binding studies in different solvents and solvent mixtures could enable such effects to be quantified. Future work will also explore strategies to increase the solubility of the structures in water to quantify their supramolecular responses to a wider range of basic, acidic, and ionic species and assess their utility in more biologically relevant chemical environments.

Overall, our results illustrate that the macrocycles are resistant to interactions with a variety of potential guests, including highly basic fluoride ions. Though the carbonyl groups act as weak hydrogen bond acceptors, the urea protons are shielded by intramolecular hydrogen bonds and bulky peripheral groups. Thus, the shape and flexibility of the macrocycles are unlikely to be significantly affected by host–guest binding effects. This predictability is crucial if the structures are to function as modular molecular hinges, providing synthetic scaffolds for a novel family of clamp-like receptors and other conformationally adaptive materials.

## Conclusions

A pair of flexible clamp-like macrocycles have been produced from inexpensive starting materials via a multigram, one-pot addition–cyclization reaction. The mechanism of this process has been explored in detail, allowing key intermediates to be isolated and characterized and the ratio of isomeric products to be reliably controlled. Furthermore, by performing part of the process in continuous flow, we have achieved conversions of 85–93% with over 80% selectivity for a single isomer. Both macrocycles act as molecular hinges, undergoing simple clamp-like transitions between isolable *syn* and *anti* conformers. Thus, this work represents a valuable addition to the synthetic toolbox of supramolecular chemists, providing an efficient route to inert, versatile, and scalable building blocks for the modular assembly of molecular machines.

## Supporting Information Available

Experimental details, ^1^H and ^13^C NMR data, mass spectra, elemental analyses, crystallographic information, kinetic plots, reaction optimization data and DFT models (PDF)

## Accession Codes

CCDC 2067586–2067591 contain the supplementary crystallographic data for this paper. These data can be obtained free of charge via www.ccdc.cam.ac.uk/data_request/cif, or by emailing data_request@ccdc.cam.ac.uk, or by contacting The Cambridge Crystallographic Data Centre, 12 Union Road, Cambridge CB2 1EZ, UK; fax: +44 1223 336033.

## Abbreviations

NMR: nuclear magnetic resonance
VT-NMR: variable-temperature nuclear magnetic resonance
SCXRD: single-crystal X-ray diffraction
PXRD: powder X-ray diffraction
DFT: density functional theory
BPR: back-pressure regulator
UPLC: ultra-performance liquid chromatography–mass spectrometry
TMS: tetramethylsilane
TBA: tetrabutylammonium.
