# Supplementary material for: High-Yielding Flow Synthesis of a Macrocyclic Molecular Hinge
Source: J Am Chem Soc. 2021 May 7;143(19):7553–65. doi: 10.1021/jacs.1c02891 (PMC8397308; doi:10.1021/jacs.1c02891)
Supplement: Supplementary file 1 — ja1c02891_si_001.pdf [file ja1c02891_si_001.pdf]

High-Yielding Flow Synthesis of a Macrocyclic Molecular Hinge

Christopher D. Jones, Laurence J. Kershaw Cook, David Marquez-Gamez, Konstantin V. Luzyanin, Jonathan W. Steed and Anna G. Slater*

Supplementary Data

Contents:

1. Experimental procedures
    1.1 Materials and methods 1
    1.2 Flow reactions 1
    1.3 Synthesis 3
    1.4 Analytical studies 5
    1.5 Computational studies 7
2. Characterization
    2.1 Compound **1** 9
    2.2 Compound **2** 12
    2.3 Compound **4a** 15
3. Single-crystal X-ray diffraction 17
4. Variable-temperature NMR spectroscopy 22
5. Conformational energy calculations 24
6. Batch synthesis 30
7. Kinetic studies 33
8. Semi-continuous flow synthesis 37
9. Host-guest binding studies 43
10. Binding energy calculations 47
11. References 51

1 Experimental procedures

1.1 Materials and methods

All solvents, reagents and starting materials were obtained from commercial suppliers and used without further purification. Elemental (CHN) analysis was performed using a Thermo FlashSmart Elemental Analyzer. NMR spectra were recorded on Bruker Avance I and Avance III 400 MHz spectrometers, with typical sample concentrations of 30 mM, and processed using Bruker TopSpin 4.0 software. Electrospray ionization (ESI+) mass spectra were obtained from 0.001% (w/v) solutions in methanol doped with 0.1% (v/v) formic acid using a Waters Acquity UPLC-MS (H-class) instrument. Powder X-ray diffraction studies were performed on a PANalytical X'Pert PRO MPD, with a Cu X-ray source, used in high throughput transmission mode with Kα focusing mirror and PIXCEL 1D detector. Single-crystal X-ray structures for *syn*-**1** and the monoclinic polymorph of **4a** were obtained at 100 K using a Rigaku 007HF Mo rotating anode (λ = 0.70926 Å) with a Saturn 724+ CCD detector and Oxford Cryostream 700+. All other single-crystal X-ray diffraction experiments were performed at 150 K using a Bruker D8 Venture dual microfocus diffractometer with a Photon 100 CMOS detector, Oxford Cryostream 700 and Mo radiation source. Structures were solved in Olex2[1] with the ShelXT structure solution program using Intrinsic Phasing[2] and refined with the ShelXL refinement package using least-squares minimisation.[3]

1.2 Flow reactions

Reactor platform

Semi-continuous syntheses were performed using a Vapourtec R-Series Flow System fitted with peristaltic pumps, PFA coiled tube reactors and a Vapourtec SF-10 pump used as an active BPR (3.0 bar). A 5 mL reactor was used for the first step of the reaction and a 10 mL reactor for the second, and the two reagent solutions were mixed at a T-piece between the two reactors. Products were collected in the steady-state regime and sampled via a VICI Valco 4-port switching valve. At-line mass spectra were obtained with a Waters Acquity UPLC-MS fitted with a BEH C18 1.7 µm 2.1 x 50 mm column, using a flow rate of 0.4 ml (typical pressure 3500 psi) and 0.1% (v/v) formic acid in methanol as the carrier solvent.

Switching valve setup

Flow reaction sample queues were prepared in the Waters software MassLynx. The switching valve was connected to the flow path of the reactor platform and the solvent inlet (port 5) and column inlet (port 6) of the UPLC Sample Manager injection valve. Sampling was performed at 6 min intervals by switching the valve from Position A to Position B for 5 s. Switching was controlled via a custom Python script and used to trigger data acquisition via a hardware connection to the Inject Hold port of the Sample Manager.

Python script for valve control

```
#!/usr/bin/env python

"""
Author: David Marquez-Gamez, Christopher Jones
University of Liverpool
Date: July 2019
Python Version: 2.7
```

```python
"""
import serial
import time

# Serial communication parameters and control command list can be found
# on the Universal Electric Actuator Instruction Manual Models EUH, EUD, and EUT
# https://www.vici.com/support/manuals/universal-actuator.pdf

# Open serial port
ser = serial.Serial(
    port        = 'COM4', # Adjust to current PC setting
    baudrate    = 9600,
    parity      = serial.PARITY_NONE,
    stopbits    = serial.STOPBITS_ONE,
    bytesize    = serial.EIGHTBITS
)
ser.isOpen()

# Delay interval in milliseconds
set_delay_ = 5000 # 5 seconds
set_delay  = str(set_delay_)

# Number of runs
set_runs = 30 # 15 samples if using 1 reactor, 30 if using 2 reactors

# Wait time in seconds
wait_time = 355 # Time from injection to next experiment; wait_time + set_delay = 360 s

                          # COMMANDS
current_mode      = 'AM'  # Displays the current actuator mode
current_pos       = 'CP'  # Displays the current position
position_AB       = 'CC'  # Sends the actuator from position A to B
position_BA       = 'CW'  # Sends the actuator from position B to A
delay_time        = 'DT'  # Displays the current delay time. If "DTnnnnn", sets the
                          # delay time from 0 to 65535 milliseconds
actuator_go       = 'GO'  # Sends the actuator to a specified position.
                          # E.g. "GOB" moves the actuator from position A to B
toggles_pos       = 'TO'  # Toggles the actuator to the opposite position
toggles_wait_back = 'TT'  # Toggles the actuator to the opposite position, waits for
                          # the delay time then returns to the original position
actuator_help     = '/?'  # Displays a list of valid commands

# Loop
for n in range(set_runs):
    print("Experiment number: ", n+1)
    print("Init position A, filling with sample")
    ser.write(actuator_go + 'A' + '\r')
    # Send command: move the actuator to Position A
    ser.write(delay_time + set_delay + '\r')
    # Send command: set the delay interval to 5 seconds
    print("Going to position B, experiment started")
    ser.write(toggles_wait_back + '\r')
    # Send command: timed toggle from A to B
    time.sleep(set_delay_/1000) # Time in seconds
    print("Back to position A, waiting for experiment end")
    # wait for next injection
    time.sleep(wait_time)

# Close serial port
ser.close()
```

1.3 Synthesis

Batch synthesis of 1 and 2

A mixture of 2-bromoethylamine hydrobromide (2.25 g, 11.0 mmol) and triethylamine (0.300 M, 2.1 eq.) in chloroform (75 mL) was stirred at 50°C until a clear solution was obtained. The cooled solution was poured onto neat tetramethylxylylene diisocyanate (2.70 g, 11.0 mmol, 1.0 eq.), stirred at room temperature for 15 minutes, then at 50°C for 6 hours. The reaction was quenched with methanol (40 mL) and the resulting solution evaporated to dryness. The residue was sonicated with cold methanol (50 mL), filtered and washed with further cold methanol (2 x 50 mL) to obtain a mixture of **1** and **2** as a white crystalline solid (2.40 g, 4.19 mmol, 76% yield, 73% selectivity for **1**). A sample of the mixture (1.00 g, 1.74 mmol) was separated by column chromatography (1:1 DCM/ethyl acetate) to obtain compounds **1** (0.35 g, 48% recovery, 36% net yield, R_f 0.27) and **2** (0.11 g, 42% recovery, 32% net yield, R_f 0.20). Single crystals suitable for SCXRD studies were obtained by recrystallizing the compounds from methanol (10 mL, 7.0 mM) with slow evaporation at room temperature. Whilst the *anti*-**1** and *syn*-**2** polymorphs accounted for the majority of the solid products, small quantities of the alternative polymorphs were observed.

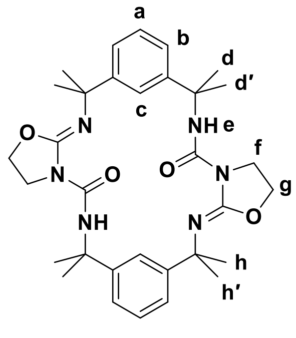

1

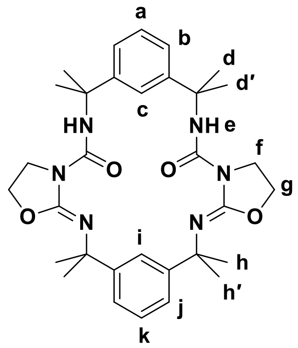

2

Compound **1**: *m*/*z* (ESI+) 575.3 [M + H] (theor. 575.3346), 597.3 [M + Na] (theor. 597.3165), 613.3 [M + K] (theor. 613.2905). Elem. Anal. Calc. (%) ($C_{32}H_{42}N_6O_4$) C 66.88, H 7.37, N 14.62; Found (%) C 66.64, H 7.31, N 14.62. ^{1}H NMR (400 MHz, $CDCl_3$) 10.84 (s, 2H, *e*), 7.49 (t, *J* = 0.9 Hz, 2H, *c*) 7.28 (t, *J* = 7.6 Hz, 2H, *a*), 7.22 (m, 4H, *b*), 3.97 (t, *J* = 3.8 Hz, 4H, *g*), 3.63 (t, *J* = 3.8 Hz, 4H, *f*), 1.70 (s, 12H, *d*, *d'*) 1.59 (s, 12H, *h*, *h'*). ^{13}C NMR (101 MHz, $CDCl_3$), 151.5, 150.3, 148.5, 147.8, 127.7, 122.0, 121.3, 77.2, 63.7, 57.8, 55.0, 42.5, 31.5, 30.1.

Crystal data for *syn*-**1**: orthorhombic, space group $Pca2_1$ (no. 29), colorless plate, a = 19.6490(4) Å, b = 10.9456(2) Å, c = 28.5401(5) Å, V = 6138.1(2) Å^3, Z = 8, Z' = 2, T = 100.0 K, μ(MoKα) = 0.084 mm^{-1}, D_{calc} = 1.244 g cm^{-3}, 72161 reflections measured (4.146° ≤ 2θ ≤ 52.772°), 12513 unique (R_{int} = 0.0968, R_{sigma} = 0.0844) which were used in all calculations. The final R_1 was 0.0714 ($I > 2\sigma(I)$), wR_2 was 0.1578 (all data) and *GoF* was 1.044.

Crystal data for *anti*-**1**: triclinic, space group P-1 (no. 2), colorless block, a = 10.0018(12) Å, b = 10.9370(13) Å, c = 15.4860(17) Å, α = 85.948(4)°, β = 72.043(3)°, γ = 76.061(4)°, V = 1564.0(3) Å^3, Z = 2, Z' = 1, T = 150.0 K, μ(MoKα) = 0.082 mm^{-1}, D_{calc} = 1.220 g cm^{-3}, 49375 reflections measured (5.128° ≤ 2θ ≤ 56.696°), 7759 unique (R_{int} = 0.0316, R_{sigma} = 0.0211) which were used in all calculations. The final R_1 was 0.0541 ($I > 2\sigma(I)$), wR_2 was 0.1521 (all data) and *GoF* was 1.037.

Compound **2**: *m*/*z* (ESI+) 575.3 [M + H] (theor. 575.3346), 597.3 [M+Na] (theor. 597.3165), 613.3 [M + K] (theor. 613.2905). Elem. Anal. Calc. (%) ($C_{32}H_{42}N_6O_4$) C 67.02, H 7.35, N 14.67; Found (%) C 66.64, H 7.31, N 14.62. ^{1}H NMR (400 MHz, $CDCl_3$) 10.63 (s, 2H, *e*), 7.50 (t, *J* = 0.8 Hz, 1H, *c*), 7.43 (t, *J* = 0.8 Hz, 1H, *i*), 7.3-7.1 (m, 6H, *a, b, j, k*), 3.99 (t, *J* = 3.8 Hz, 4H, *g*), 3.78 (t, *J* = 3.8 Hz, 4H, *f*), 1.67 (s, 12H, *d*, *d'*) 1.55 (s, 12H, *h*, *h'*). ^{13}C NMR (101 MHz, $CDCl_3$), 151.5, 150.0, 147.9, 147.3, 128.1, 127.5, 122.7, 121.9, 121.4, 121.1, 77.2, 63.9, 57.9, 55.0, 42.3, 31.6, 29.8.

Crystal data for *syn*-**2**: monoclinic, space group $P2_1/n$ (no. 14), colorless block, a = 15.4684(14) Å, b = 10.8078(9) Å, c = 19.8946(19) Å, β = 111.816(3)°, V = 3087.8(5) Å^3, Z = 4, Z' = 1, T = 150.0 K, μ(MoKα) = 0.083 mm^{-1}, D_{calc} = 1.236 g cm^{-3}, 58309 reflections measured (4.716° ≤ 2θ ≤ 56.712°), 7696 unique (R_{int} = 0.0532, R_{sigma} = 0.0353) which were used in all calculations. The final R_1 was 0.0456 ($I > 2\sigma(I)$), wR_2 was 0.1173 (all data) and *GoF* was 1.027.

Crystal data for *anti*-**2**: triclinic, space group P-1 (no. 2), colorless plate, a = 10.8032(7) Å, b = 15.6010(11) Å, c = 20.3666(15) Å, α = 68.586(3)°, β = 80.258(2)°, γ = 87.278(2)°, V = 3149.2(4) Å^3, Z = 4, Z' = 2, T = 150.0 K, μ(MoKα) = 0.082 mm^{-1}, D_{calc} = 1.212 g cm^{-3}, 98172 reflections measured (4.354° ≤ 2θ ≤ 56.804°), 15672 unique (R_{int} = 0.0373, R_{sigma} = 0.0278) which were used in all calculations. The final R_1 was 0.0469 ($I > 2\sigma(I)$), wR_2 was 0.1245 (all data) and *GoF* was 1.025.

Selective batch synthesis of 2

A mixture of 2-chloroethylamine hydrochloride (1.29 g, 11.1 mmol) and triethylamine (0.300 M, 2.1 eq.) in chloroform (75 mL) was stirred at 50°C until a clear solution was obtained. The cooled solution was poured onto neat tetramethylxylylene diisocyanate (1.35 g, 5.53 mmol, 0.50 eq.), stirred at room temperature for 1 hour, then at 50°C for 3 hours. Additional isocyanate (1.36 g, 5.57 mmol, 0.50 eq.) in chloroform (10 mL) was added and the mixture stirred at 60°C for a further 20 hours. The reaction was quenched with methanol (40 mL) and the mixture evaporated to dryness. The residue was sonicated with cold methanol (50 mL), filtered and washed with further cold methanol (2 x 50 mL) to obtain a mixture of **1** and **2** as a white crystalline solid (2.21 g, 3.85 mmol, 69% yield, 91% selectivity for **2**). A sample of the mixture (1.11 g, 1.94 mmol) was recrystallized twice from methanol to obtain pure compound **2** as a crystalline white solid (0.730 g, 1.27 mmol, 72% recovery, 45% net yield, 98% purity).

Semi-continuous synthesis of 2

Solution A: A mixture of 2-chloroethylamine hydrochloride (0.128 g, 1.10 mmol) and triethylamine (0.300 M, 2.1 eq.) in chloroform (11.4 g) was stirred at 50°C until a clear solution was obtained. The cooled solution was poured onto neat tetramethylxylylene diisocyanate (0.135 g, 0.55 mmol, 0.50 eq.) immediately before use.

Solution B: A mixture of tetramethylxylylene diisocyanate (0.135 g, 0.55 mmol, 0.50 eq.) and triethylamine (0.300 M) in chloroform (11.4 g).

Solution A (6.00 ml) was passed into Reactor 1 (5 mL, T_1 = 50-100°C) with a residence time of 30 min and flow rate of 0.167 mL min^{-1}. Solution B (5.14 ml) was added in a 1:1 steady-state volumetric ratio and the mixture transferred to Reactor 2 (10 mL, T_2 = 50-100°C) at the same flow rate. The resulting solution was collected in the steady-state regime (75-100 min, collection volume 7.98 ml), quenched immediately in methanol (20 mL) and evaporated to dryness. The composition of the crude product was determined by dissolving the residue in a weighed $CDCl_3$ solution of acetonitrile (typically 1 M, 3 g) with swirling and recording the ^{1}H NMR

spectrum of the resulting mixture. Each macrocycle was quantified by normalizing the integral of the corresponding NH signal to the CH_3 signal (δ = 2.0 ppm) of the acetonitrile standard.

Batch synthesis of 4a

A mixture of 2-chloroethylamine hydrochloride (1.29 g, 11.1 mmol) and triethylamine (0.300 M, 2.1 eq.) in chloroform (75 mL) was stirred at 50°C until a clear solution was obtained. The cooled solution was poured onto neat tetramethylxylylene diisocyanate (1.36 g, 5.57 mmol, 0.50 eq.) and the mixture stirred at 0°C for 1 hour. The reaction was quenched with methanol (40 mL) and the mixture evaporated to dryness. The resulting white solid was washed with cold chloroform (3 x 20 mL), filtered and dried in air at room temperature. Compound **4a** was obtained as a white solid (1.04 g, 2.58 mmol, 46%). Single crystals of polymorphs I and II were obtained by recrystallizing the compound from methanol (2.0 mL, 38 mM) and acetonitrile (3.5 mL, 17 mM) solutions, respectively, with slow evaporation at room temperature.

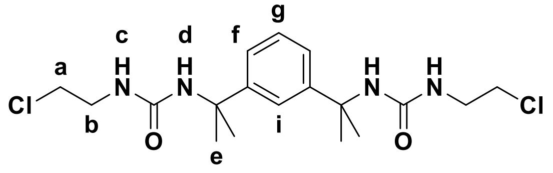

Compound **4a**: *m/z* (ESI+) 425.2 [M+Na] (theor. 425.15). Elem. Anal. Calc. (%) ($C_{18}H_{28}N_4O_2Cl_2$) C 53.60, H 7.00, N 13.89; Found (%) C 53.52, H 7.02, N 13.78. ^{1}H NMR (400 MHz, DMSO-d_6) 7.32 (t, J = 0.8 Hz, 1H, *i*), 7.2-7.1 (m, 4H, *f*, *g*), 6.43 (s, 2H, *d*), 6.11 (t, J = 3.0 Hz, 2H, *c*), 3.53 (t, J = 3.0 Hz, 4H, *a*), 3.25 (dt, J = 3.0, 3.0 Hz, 4H, *b*), 1.52 (s, 12H, *e*). ^{13}C NMR (101 MHz, DMSO-d_6) 157.3, 148.8, 127.8, 122.8, 121.7, 54.8, 45.3, 41.6, 30.6.

Crystal data for **4a**, Form I: monoclinic, space group I2/a (no. 15), colorless needle, a = 15.9023(7) Å, b = 9.1526(3) Å, c = 30.4646(14) Å, β = 105.031(4)°, V = 4282.3(3) Å^3, Z = 8, Z' = 1, T = 99.99(16) K, μ(MoKα) = 0.322 mm^{-1}, D_{calc} = 1.251 g/cm^3, 33084 reflections measured (3.294° ≤ 2θ ≤ 50.052°), 3776 unique (R_{int} = 0.0628, R_{sigma} = 0.0321) which were used in all calculations. The final R_1 was 0.0664 ($I > 2\sigma(I)$), wR_2 was 0.1686 (all data) and *GoF* was 1.108.

Crystal data for **4a**, Form II: orthorhombic, space group $P2_12_12_1$ (no. 19), colorless block, a = 10.6959(6) Å, b = 10.9131(8) Å, c = 18.0408(11) Å, V = 2105.8(2) Å^3, Z = 4, Z' = 1, T = 150.0 K, μ(MoKα) = 0.327 mm^{-1}, D_{calc} = 1.272 g/cm^3, 42165 reflections measured (4.516° ≤ 2θ ≤ 67.082°), 8232 unique (R_{int} = 0.0485, R_{sigma} = 0.0371) which were used in all calculations. The final R_1 was 0.0475 ($I > 2\sigma(I)$), wR_2 was 0.1358 (all data) and *GoF* was 1.039.

1.4 Analytical studies

Rate constant measurements (room temperature)

A mixture of 2-chloroethylamine hydrochloride (128 mg, 1.10 mmol) or 2-bromoethylamine hydrobromide (226 mg, 1.10 mmol) in a $CDCl_3$ solution of triethylamine (0.300 M, 2.1 eq., 11.4 g) was stirred at 50°C until a clear solution was obtained. The cooled solution was poured onto neat tetramethylxylylene diisocyanate (135 mg, 0.55 mmol, 0.50 eq.) and stirred. Kinetic data were obtained by taking 6-10 in-situ NMR measurements of an individual sample over a period of 5 hours for the reaction of 2-chloroethylamine and 30 minutes for the reaction of 2-bromoethylamine. Integrals of the macrocycle NH signals were normalized to the weighted average integrals of the triethylamine CH_2 (quartet, δ = 2.95 ppm) and CH_3 (triplet, δ = 1.30 ppm) signals. First-order rate

constants were calculated by plotting the natural logarithms of the normalized and corrected NH integrals against the sampling time and measuring the gradient of the initial straight-line region.

Rate constant measurements (heated)

A mixture of 2-chloroethylamine hydrochloride (128 mg, 1.10 mmol) in a $CDCl_3$ solution of triethylamine (0.300 M, 2.1 eq., 11.4 g) was stirred at 50°C until a clear solution was obtained. The cooled solution was poured onto neat tetramethylxylylene diisocyanate (135 mg, 0.55 mmol, 0.50 eq.) and stirred continuously in a thermostatically controlled water bath set to 30, 40 or 50°C. Kinetic data for heated solutions were measured by taking aliquots at intervals of 15-60 minutes and recording their ^{1}H NMR spectra. Integrals of the macrocycle NH signals were normalized to the weighted average integrals of the triethylamine CH_2 (quartet, δ = 2.95 ppm) and CH_3 (triplet, δ = 1.30 ppm) signals. For each measurement, the sampling and NMR acquisition times were both recorded, and the NH integrals corrected based on the measured rate laws of the room-temperature reactions. First-order rate constants were calculated from 5-10 data points as in the room-temperature kinetic studies. Fewer data points were used at higher temperatures due to the shorter timescale of the reaction and more rapid divergence from first-order kinetics.

Conversion and selectivity measurements

A mixture of 2-chloroethylamine hydrochloride (128 mg, 1.10 mmol) or 2-bromoethylamine hydrobromide (226 mg, 1.10 mmol) in a chloroform solution of triethylamine (0.300 M, 2.1 eq., 11.4 g) was stirred at 50°C until a clear solution was obtained. In Method A, the cooled solution was poured onto neat tetramethylxylylene diisocyanate (269 mg, 1.10 mmol, 1.0 eq.) and stirred continuously at room temperature for 6 hours. In Method B, the cooled solution was poured onto the neat isocyanate (135 mg, 0.55 mmol, 0.50 eq.), stirred continuously at room temperature for 3 hours, mixed with additional neat isocyanate (135 mg, 0.55 mmol, 0.50 eq.) then stirred at room temperature for a further 3 hours. The reaction was quenched by shaking with methanol (5 mL) and the resulting solution evaporated to dryness. The residue was dissolved in a weighed $CDCl_3$ solution of acetonitrile (typically 1 M, 3 g) with swirling and a sample taken for ^{1}H NMR analysis. Each macrocycle was quantified by normalizing the integral of the corresponding NH signal to the CH_3 signal (singlet, δ = 2.0 ppm) of the acetonitrile standard.

Flow and batch comparison at 60°C

A mixture of 2-chloroethylamine hydrochloride (97 mg, 84 mmol) in a chloroform solution of triethylamine (0.300 M, 2.1 eq., 8.64 g) was stirred at 50°C until a clear solution was obtained. The cooled solution was poured onto neat tetramethylxylylene diisocyanate (102 mg, 42 mmol, 0.50 eq.) and the mixture was left to stand at room temperature for 30 minutes, matching the average time for steady-state conditions to be established in flow. The mixture was stirred at 60°C for 30 minutes, mixed with a solution of tetramethylxylylene diisocyanate (102 mg, 42 mmol, 0.50 eq.) and triethylamine in chloroform (0.300 M, 8.64 g) and stirred at 60°C for a further 30 minutes. The reaction was quenched by shaking with methanol (5 mL) and the resulting solution evaporated to dryness. The residue was dissolved in a weighed $CDCl_3$ solution of acetonitrile (typically 1 M, 3 g) with swirling and a sample taken for ^{1}H NMR analysis. Each macrocycle was quantified by normalizing the integral of the corresponding NH signal to the CH_3 signal (δ = 2.0 ppm) of the acetonitrile standard. The results were compared with semi-continuous flow experiments performed at $T_1 = T_2 = 60$°C.

Host-guest binding measurements

To quantify the binding of guests by **1** and **2**, a range of species (100-200 eq.) were added to stock solutions of the macrocycles in $CDCl_3$ (9.0 mM) with tetramethylsilane (TMS, 0.05% w/v) as an internal reference (δ = 0.0 ppm). Ionic guests were added as tetrabutylammonium (TBA) salts. For each guest producing significant changes in the NMR spectrum (**1** + methanol, **2** + methanol, acetonitrile, TBAF trihydrate and TBACl), weighed aliquots (15-200 mg) of the host-guest solution were added sequentially to a known mass of the macrocycle stock (typically 1.0 g) in a single NMR tube. After each addition, the sample was inverted multiple times to ensure complete mixing then analyzed by ^{1}H NMR spectroscopy at room temperature. A total of 10-14 aliquots were added, reaching total guest concentrations of 40-130 eq. (maximum concentrations of the TBA salts were lower due to their limited solubility). Binding isotherms were produced by measuring changes in the chemical shift of the macrocycle NH signal, $\Delta\delta$, for increasing concentrations of the added guest. Data were fitted to 1:1 binding isotherms using a Nelder-Mead algorithm in the online software BindFit.[4, 5]

1.5 Computational studies

Geometry optimizations

Atomic coordinates of the macrocycles were extracted from their single-crystal X-ray structures and optimized in Gaussian 16, Revision A.03,[6] using the DFT method B3LYP[7] with tight SCF convergence. Structures were modeled in the basis set[8] 6-31+G* and refined in the larger basis sets 6-31++G**, def2-TZVP[9] and aug-cc-PVDZ.[10] Optimizations were performed both with and without the D3BJ dispersion correction,[11] and their accuracy evaluated by comparison with VT-NMR data.

Conformational transitions

Activation energies for conformational transitions were estimated by varying the C=N-C-C torsion angle between one oxazolidine ring and its *anti* methyl group and re-optimizing the structure after each scan step. Geometry optimizations were performed using the method B3LYP and basis set 6-31+G*. Torsions were incremented in steps of 0.2° near the energetic maximum and 1-5° elsewhere, depending on the smoothness of the geometric changes. The final activation energies were refined by fixing the scanned torsion angle of the highest-energy geometry and re-optimizing the structure in the larger basis set aug-cc-PVDZ. All calculations were performed both with and without the D3BJ dispersion correction, and their accuracy evaluated by comparison with VT-NMR data.

Conformational energy landscapes

The conformational energies of **6** and its theoretical analogue **7** were analyzed by fixing one methyl-imine (C=N-C-C) torsion angle, incrementing the other in steps of 5°, and re-optimizing the structure after each scan step. Geometry optimizations were performed using the method B3LYP and basis set 6-31+G* with the D3BJ dispersion correction. The calculations were repeated using eight different starting conformations, resulting in 8-16 replicate energy values for each combination of torsion angles. Conformational energies were obtained by calculating the mean energies of symmetry-unique geometries and subtracting the average energy of the most stable conformer. The results were analyzed graphically using triangular contour plots, while convergence of was assessed by comparing the mean energies of symmetry-equivalent torsion angle pairs.

Binding energy calculations

Model 1:1 host-guest complexes of **1** and **2** were based on low-energy, partially open macrocycle geometries, selected from the calculated pathways of the *syn-anti* and *anti-syn* conformational transitions. Guest molecules were added manually to produce a variety of configurations, spanning all possible relative orientations and hydrogen bond donor-acceptor pairs. Geometries were optimized using the method B3LYP and basis set 6-31+G*, then refined in the larger basis set 6-31++G**. Binding energies were calculated by subtracting the energy of the complex from the energies of the free host and guest in their optimized geometries. A sample of structures were re-optimized using a counterpoise correction for basis set superposition error,[12] but this procedure was found to have a negligible impact on the calculation outcome while substantially increasing computation time.

2 Characterization

2.1 Compound 1

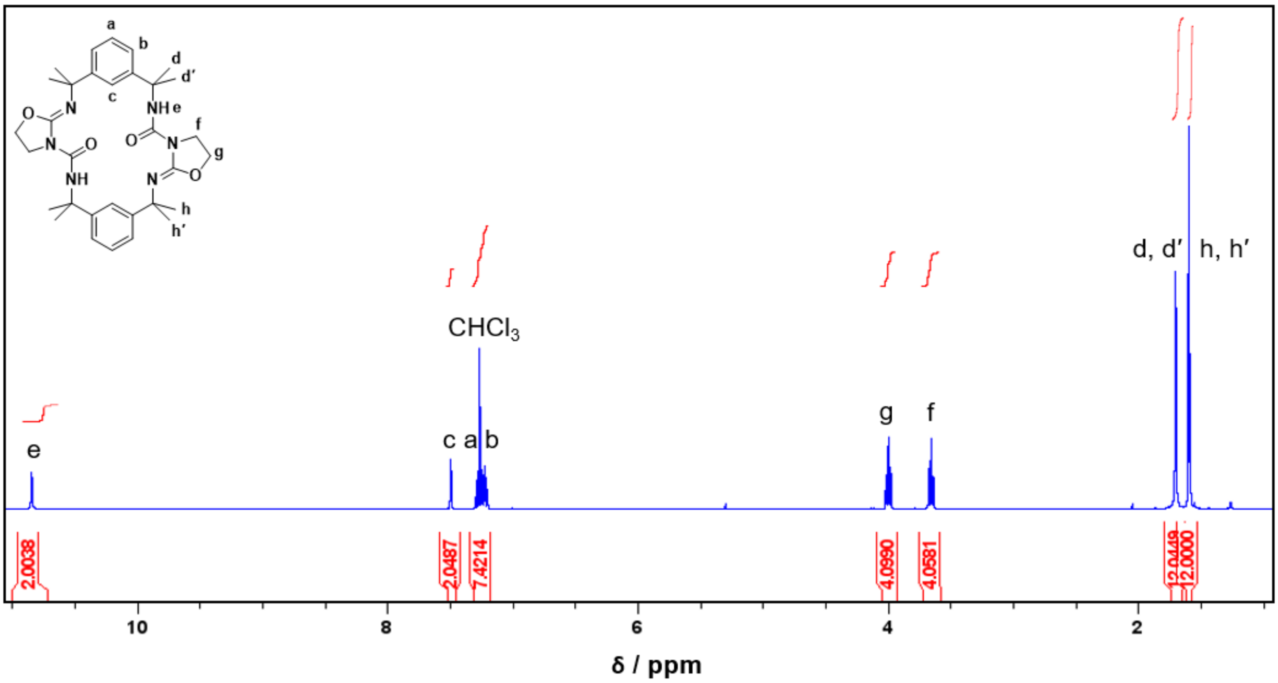

Fig. S1 ^{1}H NMR spectrum of **1** in $CDCl_3$ with structural assignments (inset) and integrals shown. Accurate integration of the multiplet corresponding to the aryl proton *a* is not possible due to overlap with the $CHCl_3$ signal.

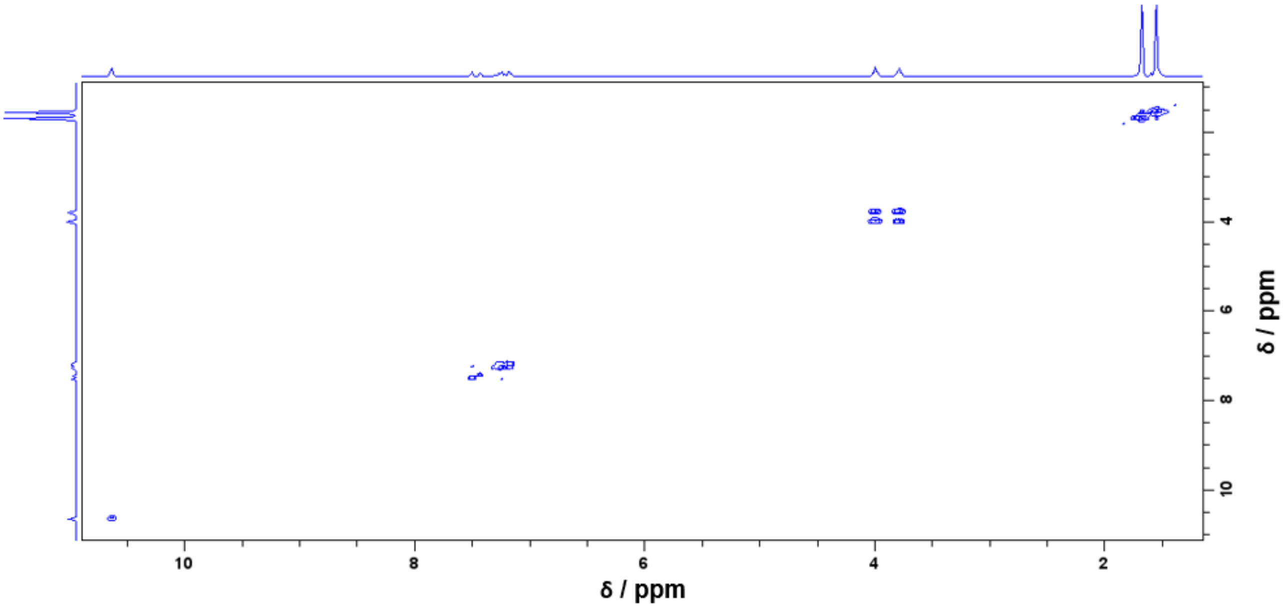

Fig. S2 $^{1}H,^{1}H$-COSY NMR spectrum of **1** in $CDCl_3$.

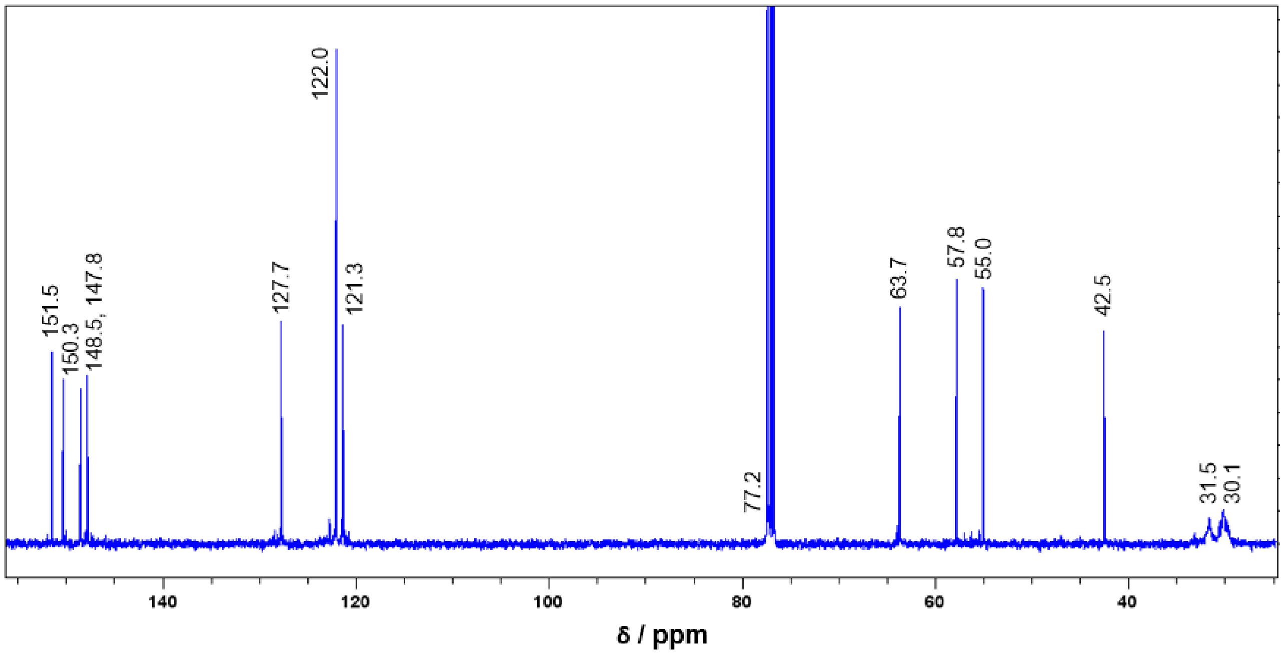

Fig. S3 $^{13}C\{^1H\}$ NMR spectrum of **1** in $CDCl_3$.

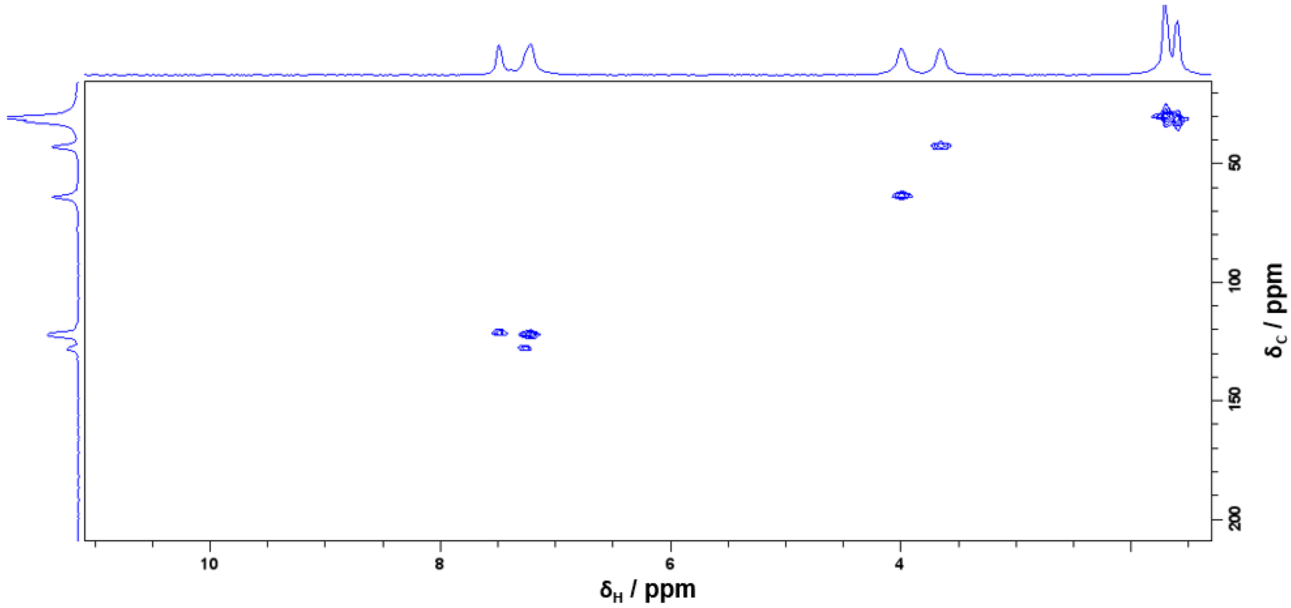

Fig. S4 $^1H,^{13}C$-HSQC NMR spectrum of **1** in $CDCl_3$.

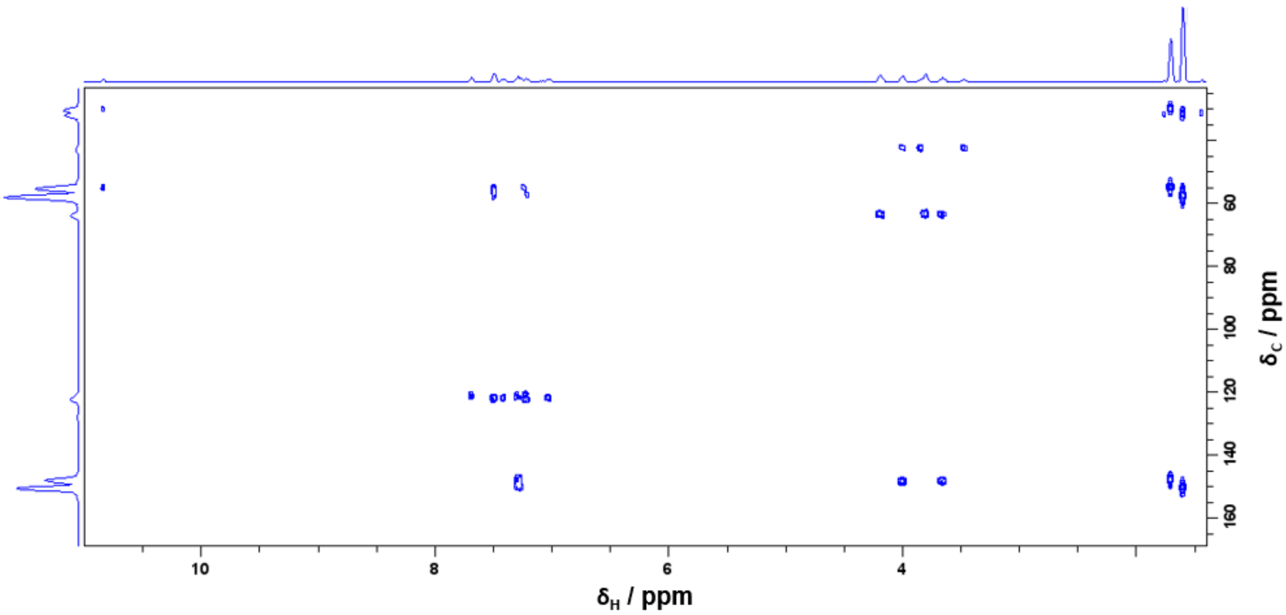

Fig. S5 $^{1}H,^{13}C$-HMBC NMR spectrum of **1** in $CDCl_3$.

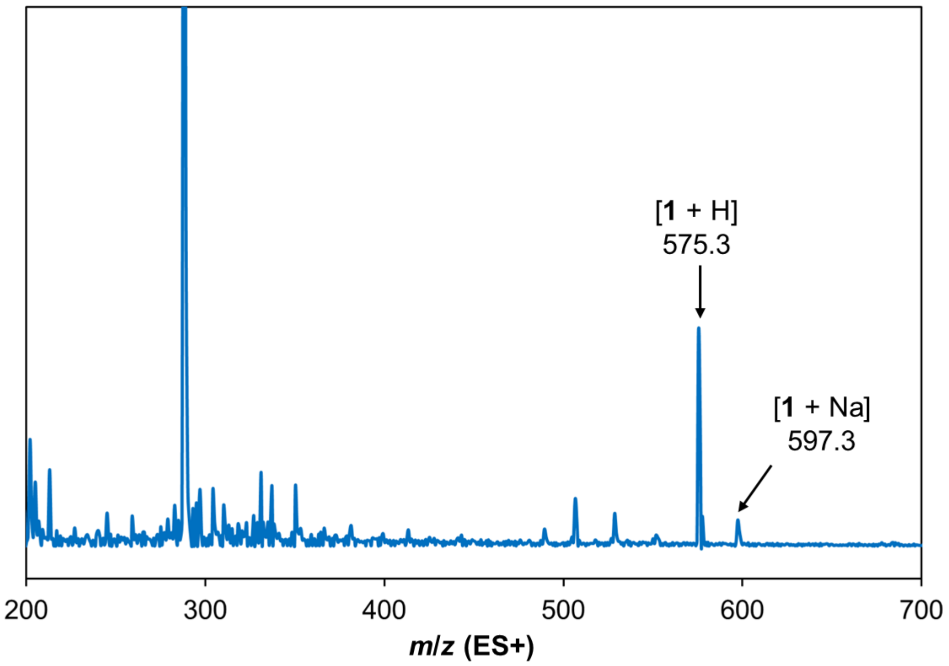

Fig. S6 Mass spectrum (ESI+) of **1** in methanol doped with 0.1% (v/v) formic acid.

2.2 Compound 2

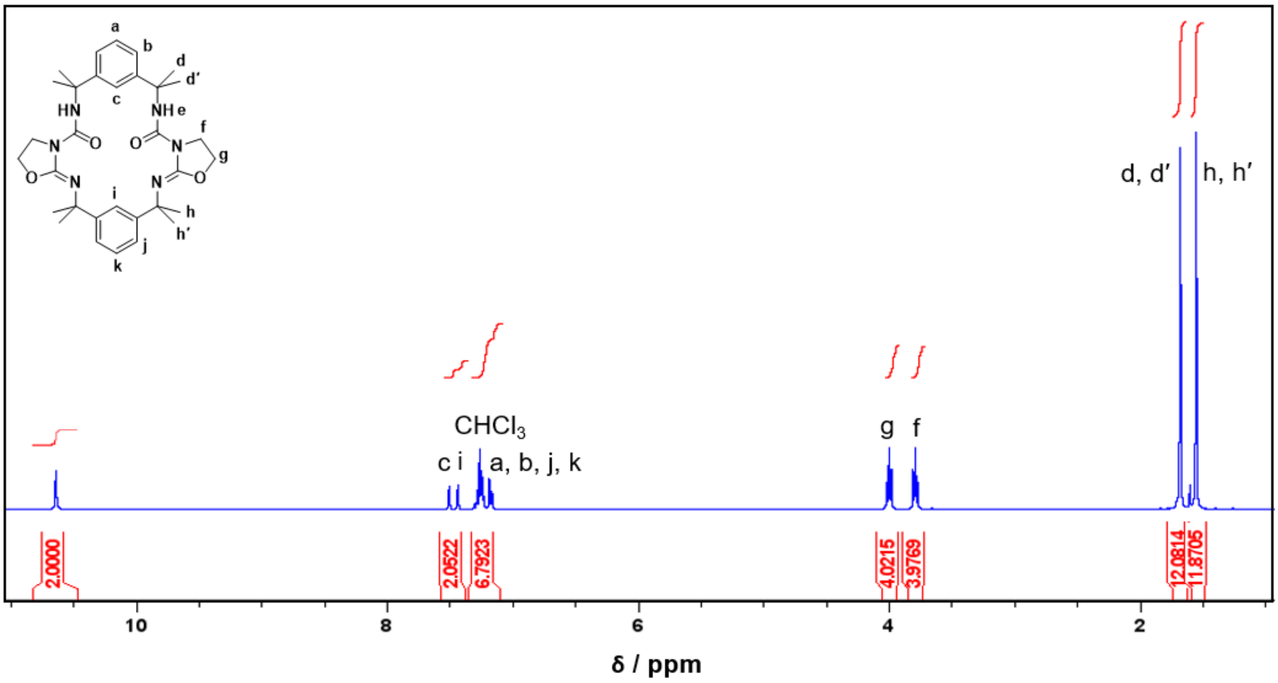

Fig. S7 ^{1}H NMR spectrum of **2** in $CDCl_3$ with structural assignments (inset) and integrals shown. Accurate integration of the multiplet corresponding to aryl protons *a*, *b*, *j* and *k* is not possible due to overlap with the $CHCl_3$ signal.

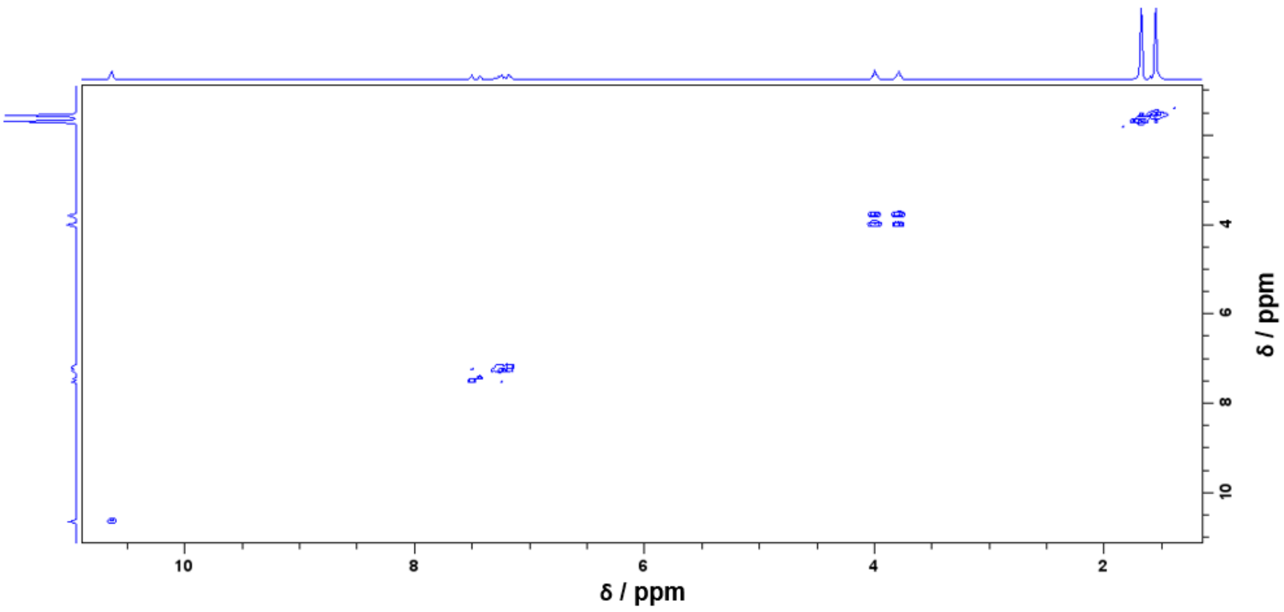

Fig. S8 ^{1}H,^{1}H-COSY NMR spectrum of **2** in $CDCl_3$.

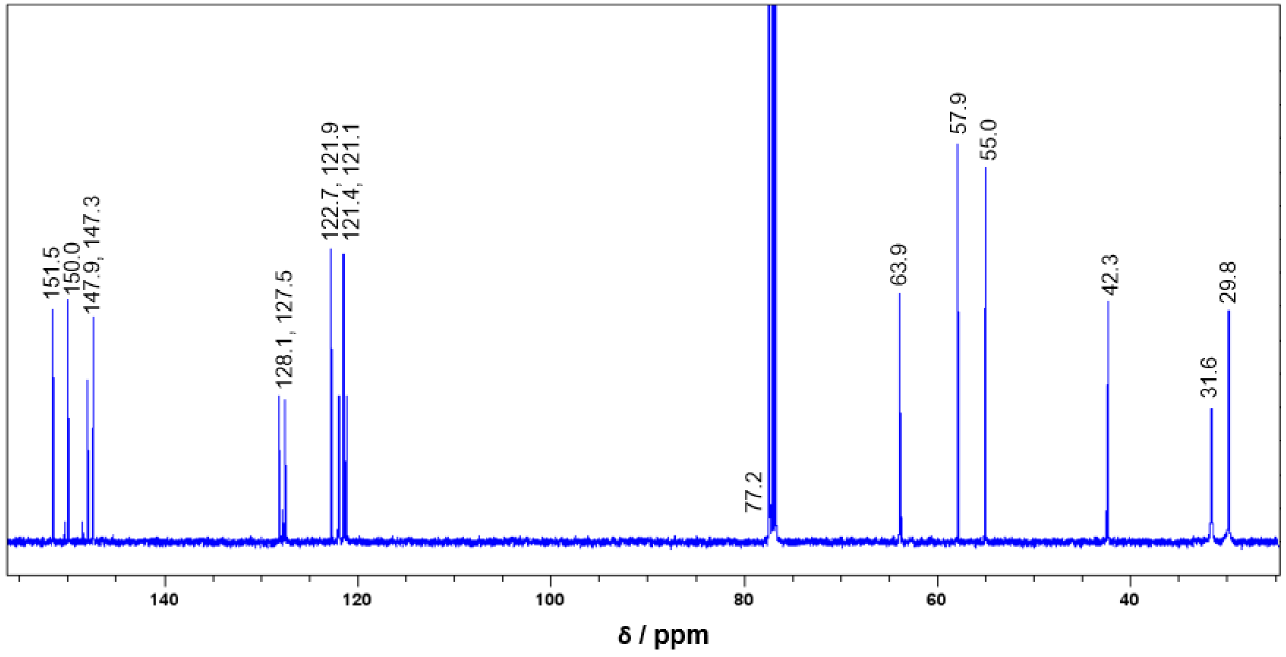

Fig. S9 $^{13}C\{H\}$ NMR spectrum of **2** in $CDCl_3$.

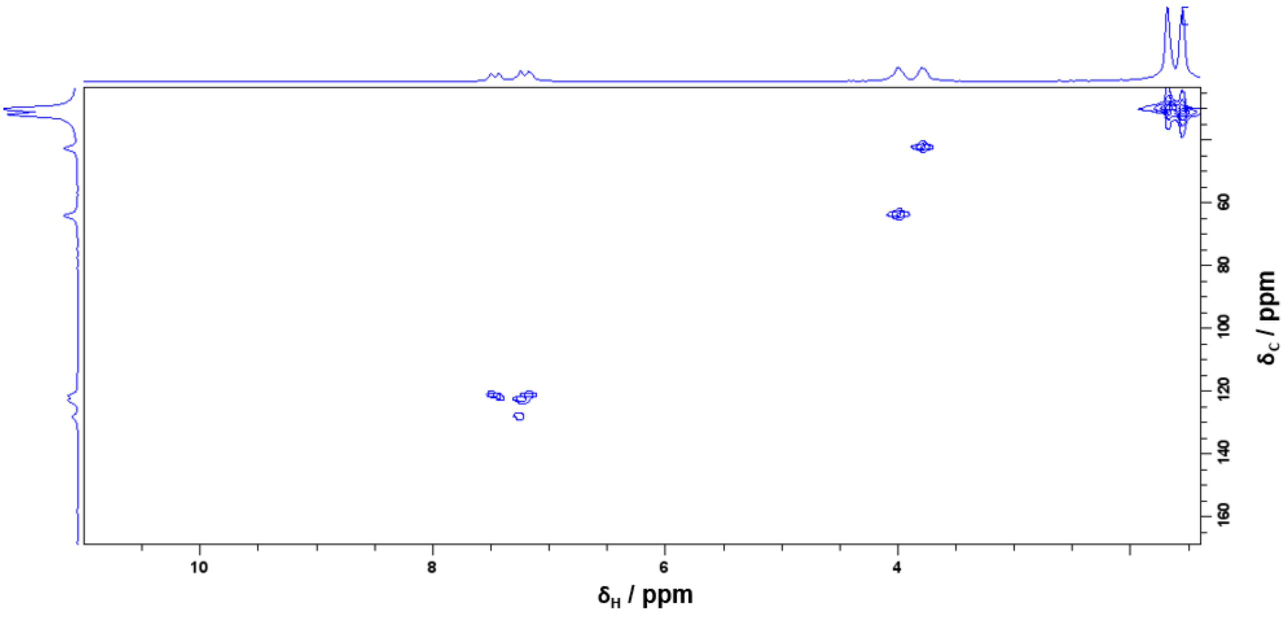

Fig. S10 $^{1}H,^{13}C$-HSQC NMR spectrum of **2** in $CDCl_3$.

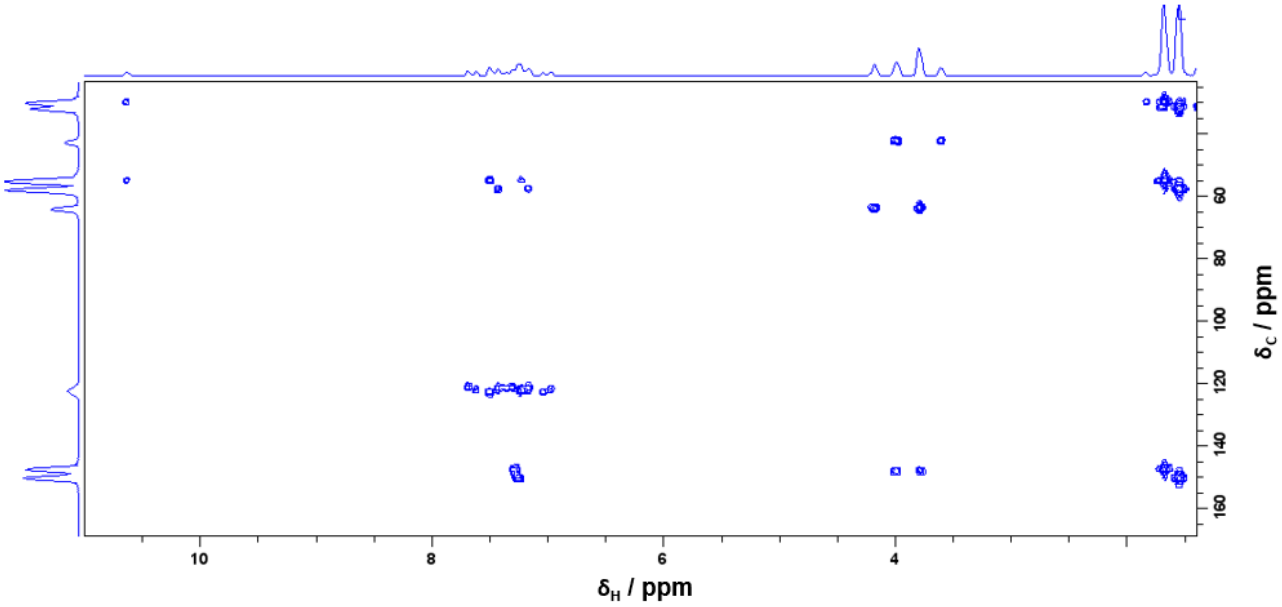

Fig. S11 ^{1}H,^{13}C-HMBC NMR spectrum of **2** in $CDCl_3$.

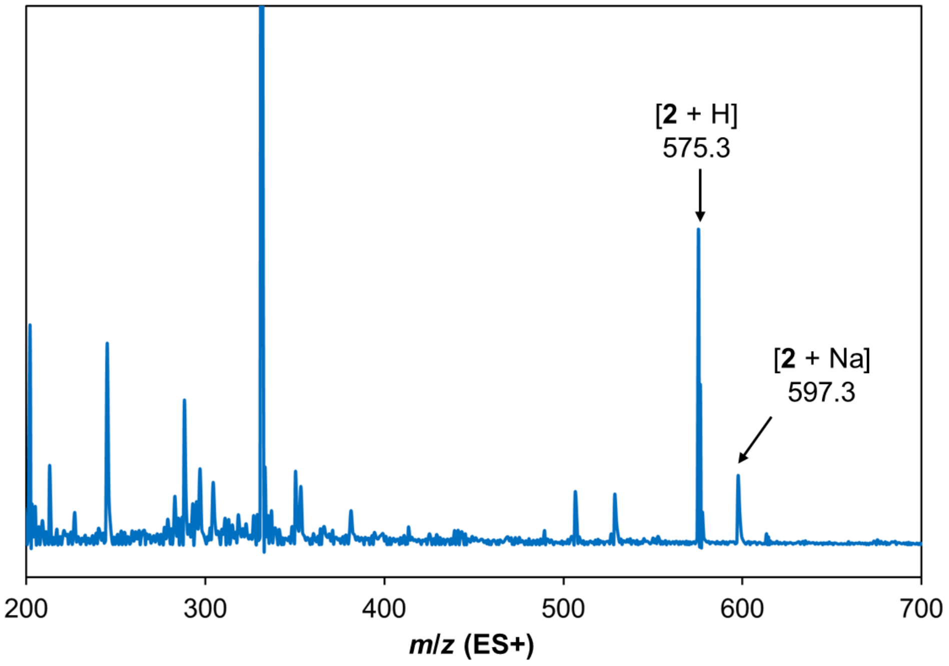

Fig. S12 Mass spectrum (ESI+) of **2** in methanol doped with 0.1% (v/v) formic acid.

2.3 Compound 4a

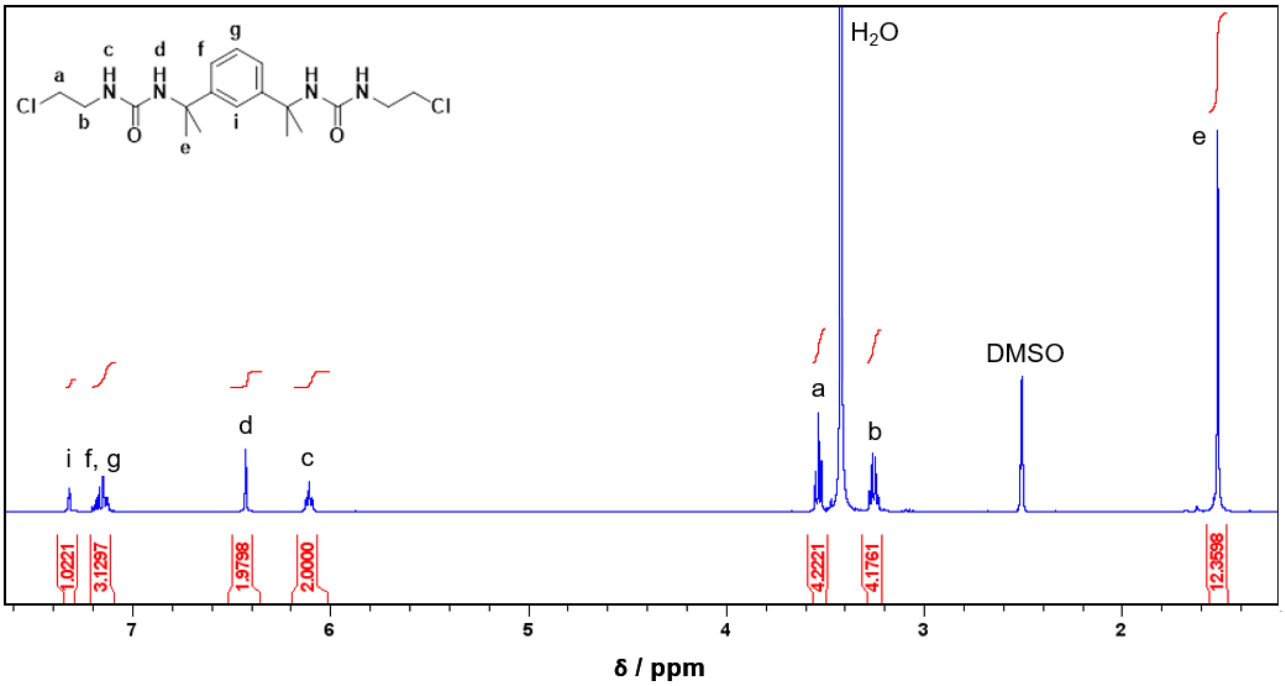

Fig. S13 ^{1}H NMR spectrum of **4a** in DMSO-d_6 with structural assignments (inset) and integrals shown.

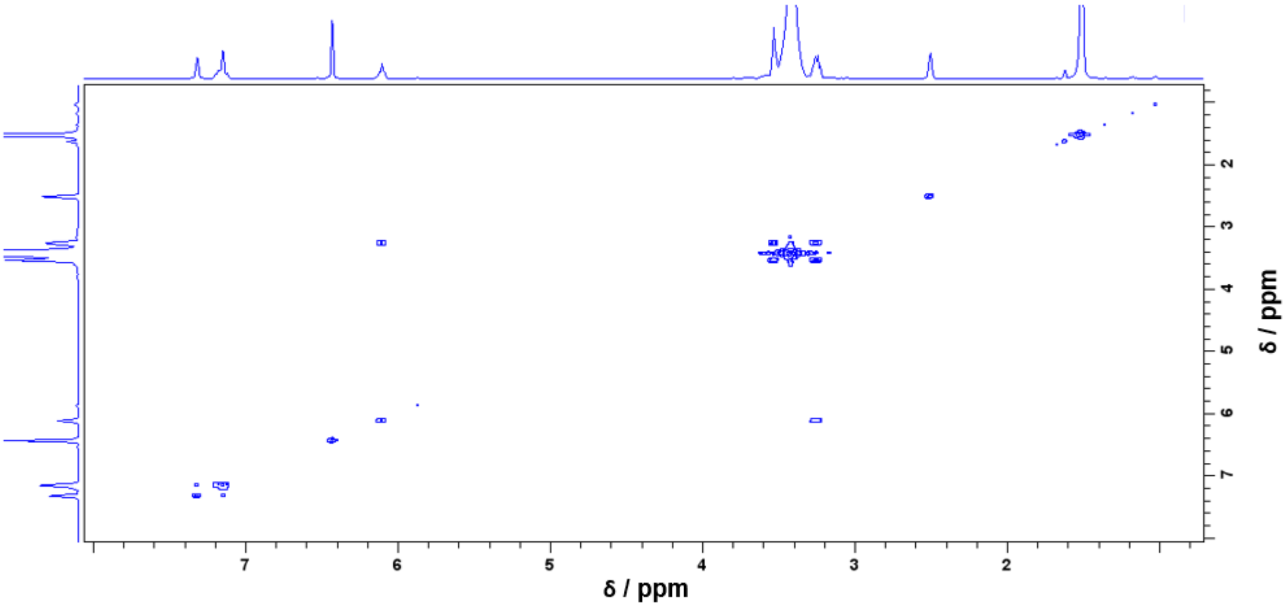

Fig. S14 ^{1}H,^{13}C-COSY NMR spectrum of **4a** in DMSO-d_6.

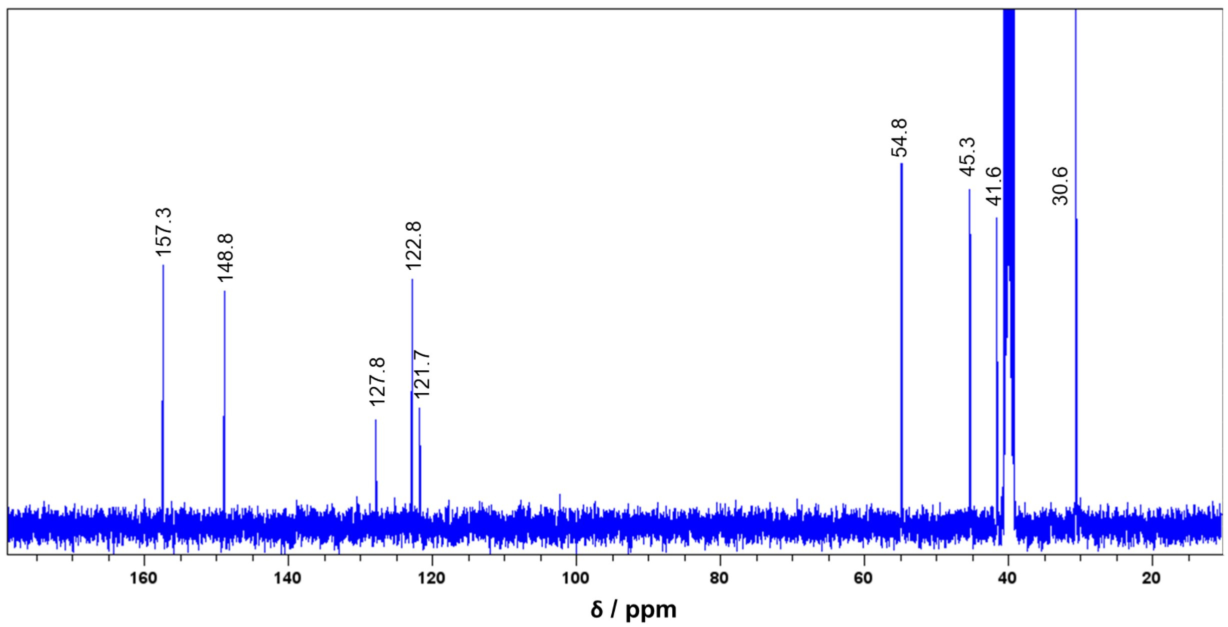

Fig. S15 $^{13}C\{^{1}H\}$ NMR spectrum of **4a** in DMSO-d_6.

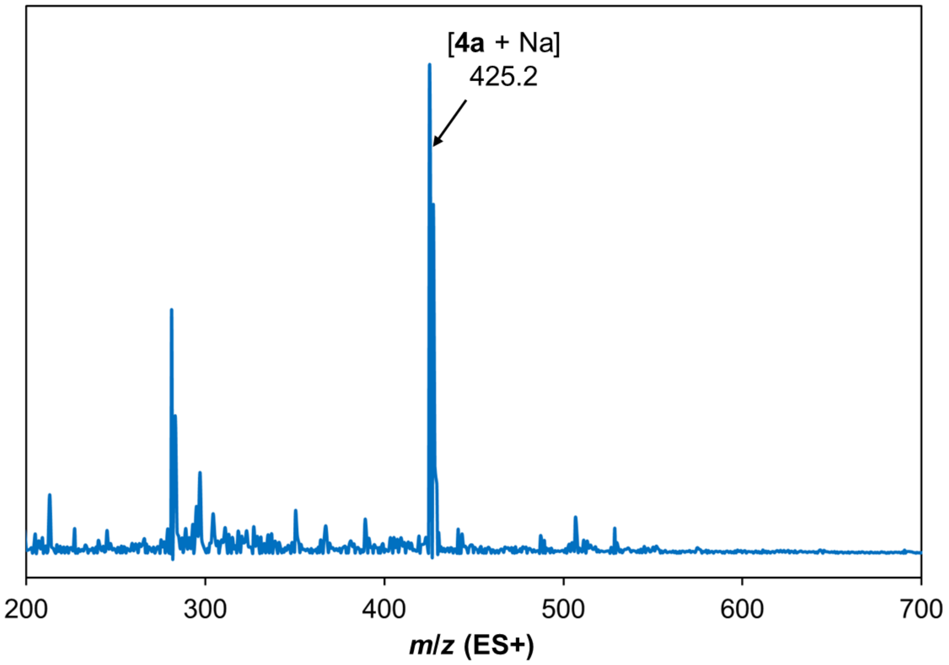

Fig. S16 Mass spectrum (ESI+) of **4a** in methanol doped with 0.1% (v/v) formic acid.

3 Single-crystal X-ray diffraction

	syn-**1**	*anti*-**1**	*syn*-**2**	*anti*-**2**
**Formula weight**	574.71	574.71	574.71	574.71
T **/ K**	100.0	150.0	150.0	150.0
**Crystal system**	orthorhombic	triclinic	monoclinic	triclinic
**Space group**	$Pca2_1$	P-1	$P2_1/n$	P-1
a **/ Å**	19.6490(4)	10.0018(12)	15.4684(14)	10.8032(7)
b **/ Å**	10.9456(2)	10.9370(13)	10.8078(9)	15.6010(11)
c **/ Å**	28.5401(5)	15.4860(17)	19.8946(19)	20.3666(15)
**α / °**	90	85.948(4)	90	68.586(3)
**β / °**	90	72.043(3)	111.816(3)	80.258(2)
**γ / °**	90	76.061(4)	90	87.278(2)
V **/ Å**^{3}	6138.1(2)	1564.0(3)	3087.8(5)	3149.2(4)
Z	8	2	4	4
Z'	2	1	1	2
ρ_{calc} **/ g cm**$^{-3}$	1.244	1.220	1.236	1.212
**μ / mm**$^{-1}$	0.084	0.082	0.083	0.082
F**(000)**	2464	616	1232	1232
**Crystal size / mm**^{3}	0.18 x 0.04 x 0.04	0.25 × 0.15 × 0.15	0.55 × 0.34 × 0.11	0.40 × 0.25 × 0.10
**Radiation**	MoKα	MoKα	MoKα	MoKα
**2θ range / °**	4.146 to 52.772	4.738 to 56.696	4.716 to 56.712	4.354 to 56.804
**Index ranges**	$-24 \le h \le 24$<br>$-10 \le k \le 13$<br>$-35 \le l \le 35$	$-13 \le h \le 13$<br>$-14 \le k \le 14$<br>$-20 \le l \le 20$	$-20 \le h \le 20$<br>$-14 \le k \le 14$<br>$-26 \le l \le 26$	$-14 \le h \le 14$<br>$-20 \le k \le 20$<br>$-26 \le l \le 27$
**Reflections collected**	72161	49375	58309	98172
**Independent reflections**	12513	7759	7696	15672
R_{int}	0.0968	0.0316	0.0532	0.0373
R_{sigma}	0.0844	0.0211	0.0353	0.0278
**Data/restraints/parameters**	12513/1/773	7759/0/395	7696/0/395	15672/0/789
**Goodness-of-fit on** F^2	1.044	1.037	1.027	1.025
**Final** R **indexes [**$I \ge 2\sigma(I)$**]**	R_1 = 0.0714<br>wR_2 = 0.1440	R_1 = 0.0541<br>wR_2 = 0.1460	R_1 = 0.0456<br>wR_2 = 0.1054	R_1 = 0.0469<br>wR_2 = 0.1157
**Final** R **indexes [all data]**	R_1 = 0.1014<br>wR_2 = 0.1578	R_1 = 0.0619<br>wR_2 = 0.1521	R_1 = 0.0695<br>wR_2 = 0.1173	R_1 = 0.0630<br>wR_2 = 0.1245
**Largest peak/hole / e Å**$^{-3}$	0.72/-0.31	0.37/-0.45	0.36/-0.24	0.47/-0.44
**Flack parameter**	0.5(9)	-	-	-

Table S1 Crystal data for *syn* and *anti* polymorphs of **1** and **2**. The structure of *syn*-**1** unambiguously confirms the connectivity of the molecule but displays high R_1 and low I/σ values due to the small size and weak diffraction of the sample crystal. Attempts to obtain larger, more strongly diffracting crystals were unsuccessful. The asymmetric units of the chiral isomers *syn*-**1** and *anti*-**2** are racemic, containing one molecule each of the two possible enantiomers. It should be noted that no chiral excess is possible due to the lack of chirality in the starting materials. The absolute structure of the non-centrosymmetric crystal of *syn*-**1** is in an arbitrary configuration due to the low precision of the calculated Flack parameter.

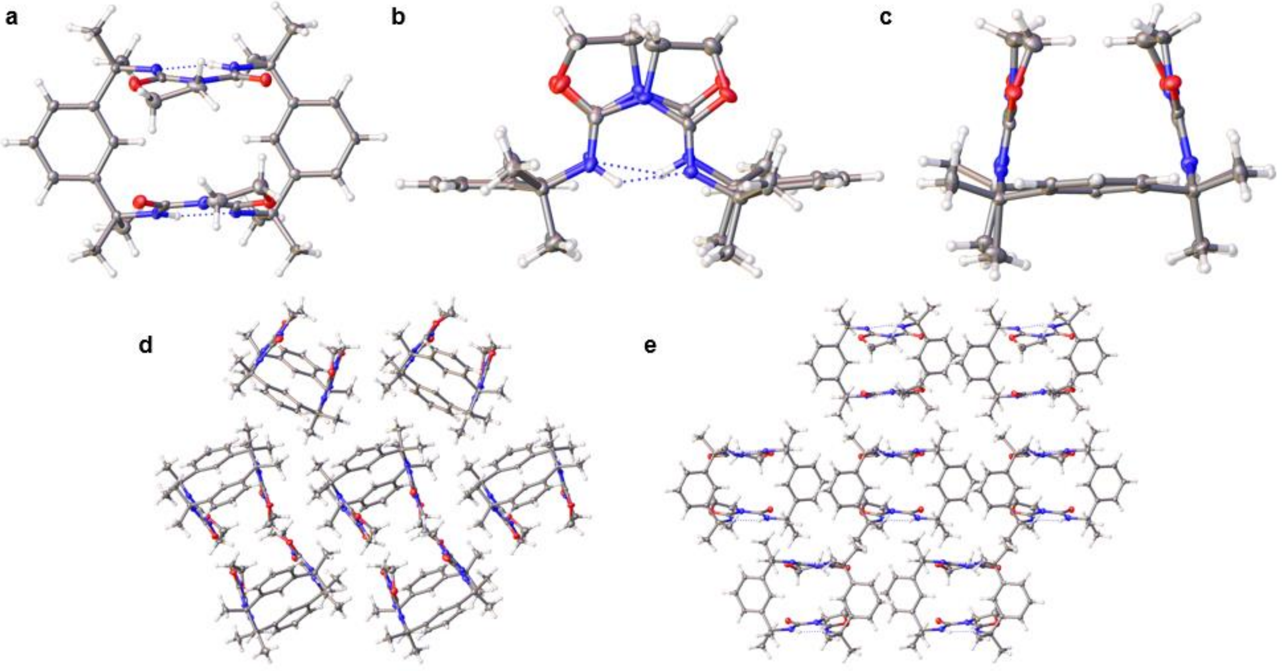

Fig. S17 SCXRD geometry of *syn*-**1** viewed from the (a) top and (b) side and (c) along the phenyl-phenyl axis, and crystal packing viewed along (d) (010) and (e) (101).

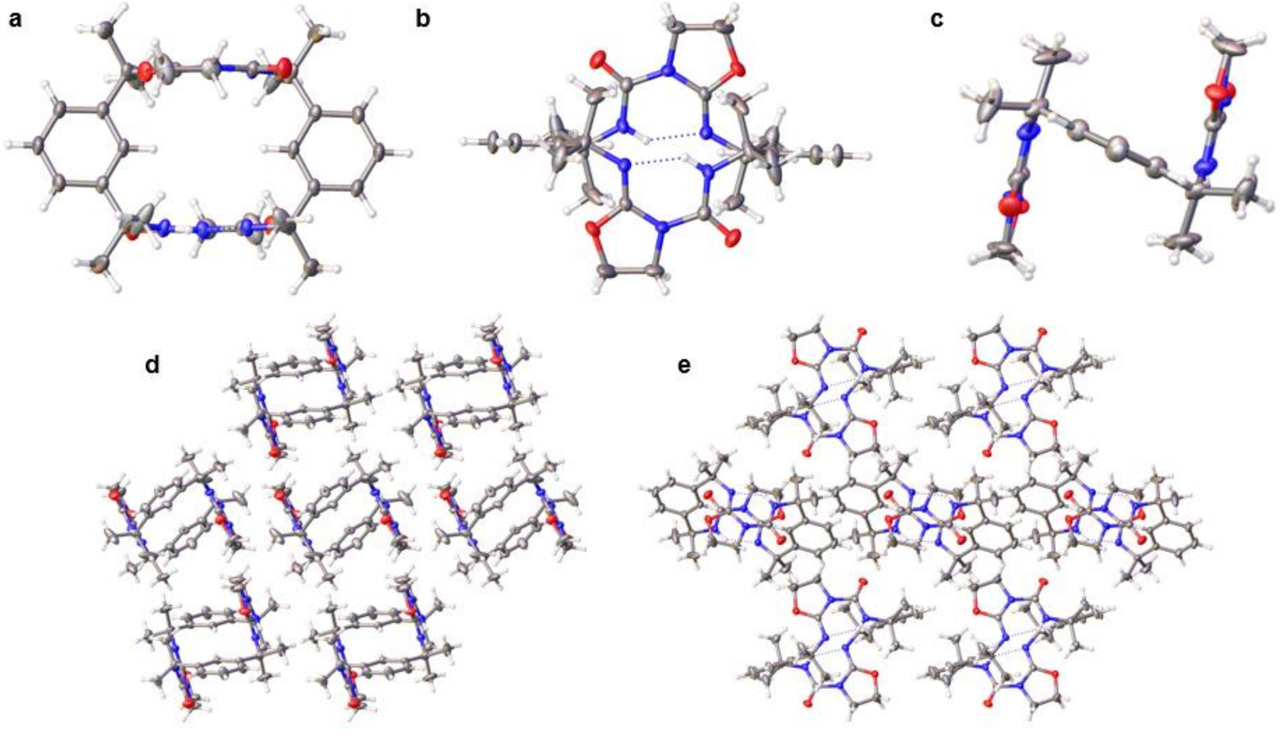

Fig. S18 SCXRD geometry of *anti*-**1** viewed from the (a) top and (b) side and (c) along the phenyl-phenyl axis, and crystal packing viewed along (d) (010) and (e) (100).

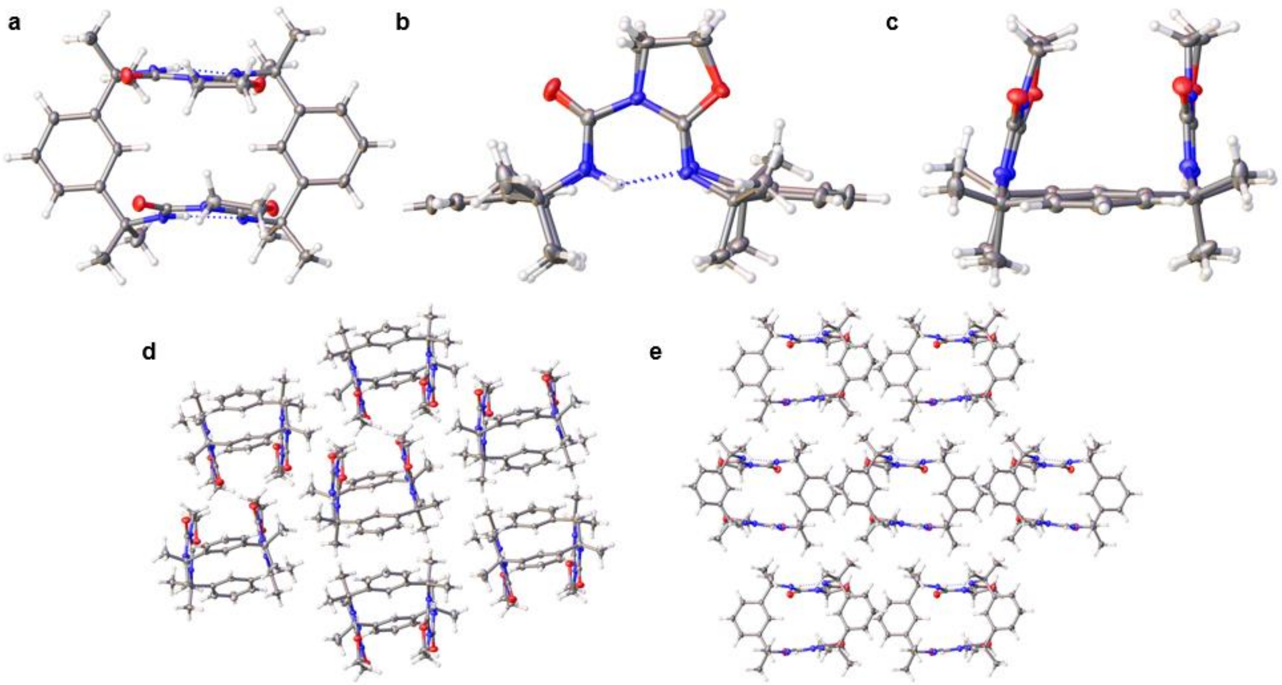

Fig. S19 SCXRD geometry of *syn*-**2** viewed from the (a) top and (b) side and (c) along the phenyl-phenyl axis, and crystal packing viewed along (d) (010) and (e) (100).

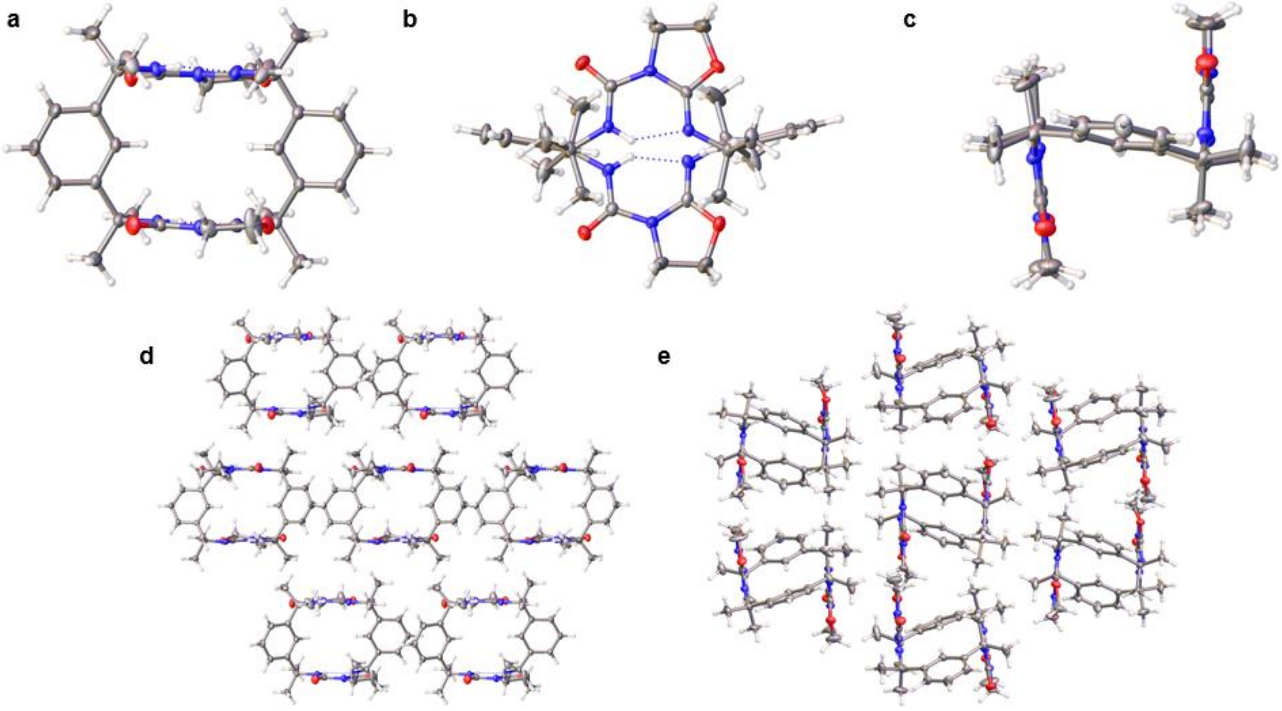

Fig. S20 SCXRD geometry of *anti*-**2** viewed from the (a) top and (b) side and (c) along the phenyl-phenyl axis, and crystal packing viewed along (d) (010) and (e) (100).

	**4a** Form I	**4a** Form II
**Formula weight**	403.34	403.34
***T* / K**	100.0	150.0
**Crystal system**	monoclinic	orthorhombic
**Space group**	I2/a	$P2_12_12_1$
***a* / Å**	15.9023(7)	10.6959(6)
***b* / Å**	9.1526(3)	10.9131(8)
***c* / Å**	30.4646(14)	18.0408(11)
**α / °**	90	90
**β / °**	105.031(4)	90
**γ / °**	90	90
**V / Å^3**	4282.3(3)	2105.8(2)
Z	8	4
Z'	1	1
**ρ_{calc} / g cm^{-3}**	1.251	1.272
**μ / mm^{-1}**	0.322	0.327
***F*(000)**	1712	856
**Crystal size / mm^3**	0.20 x 0.05 x 0.04	0.25 × 0.22 × 0.15
**Radiation**	MoKα	MoKα
**2θ range / °**	3.294 to 50.052	4.516 to 67.082
**Index ranges**	$-18 \leq h \leq 18$<br>$-10 \leq k \leq 10$<br>$-36 \leq l \leq 36$	$-16 \leq h \leq 16$<br>$-13 \leq k \leq 16$<br>$-26 \leq l \leq 28$
**Reflections collected**	33084	42165
**Independent reflections**	3776	8232
**R_{int}**	0.0628	0.0485
**R_{sigma}**	0.0321	0.0371
**Data/restraints/parameters**	3776/0/240	8232/0/273
**Goodness-of-fit on F^2**	1.108	1.039
**Final *R* indexes [$I \geq 2\sigma(I)$]**	R_1 = 0.0664<br>wR_2 = 0.1634	R_1 = 0.0475<br>wR_2 = 0.1303
**Final *R* indexes [all data]**	R_1 = 0.0746<br>wR_2 = 0.1686	R_1 = 0.0548<br>wR_2 = 0.1358
**Largest peak/hole / e Å^{-3}**	0.82/-0.49	0.57/-0.76
**Flack parameter**	-	0.14(2)

Table S2 Crystal data for polymorphs of **4a**. The absolute structure of the non-centrosymmetric crystal of Form II is in an arbitrary configuration due to the low precision of the calculated Flack parameter.

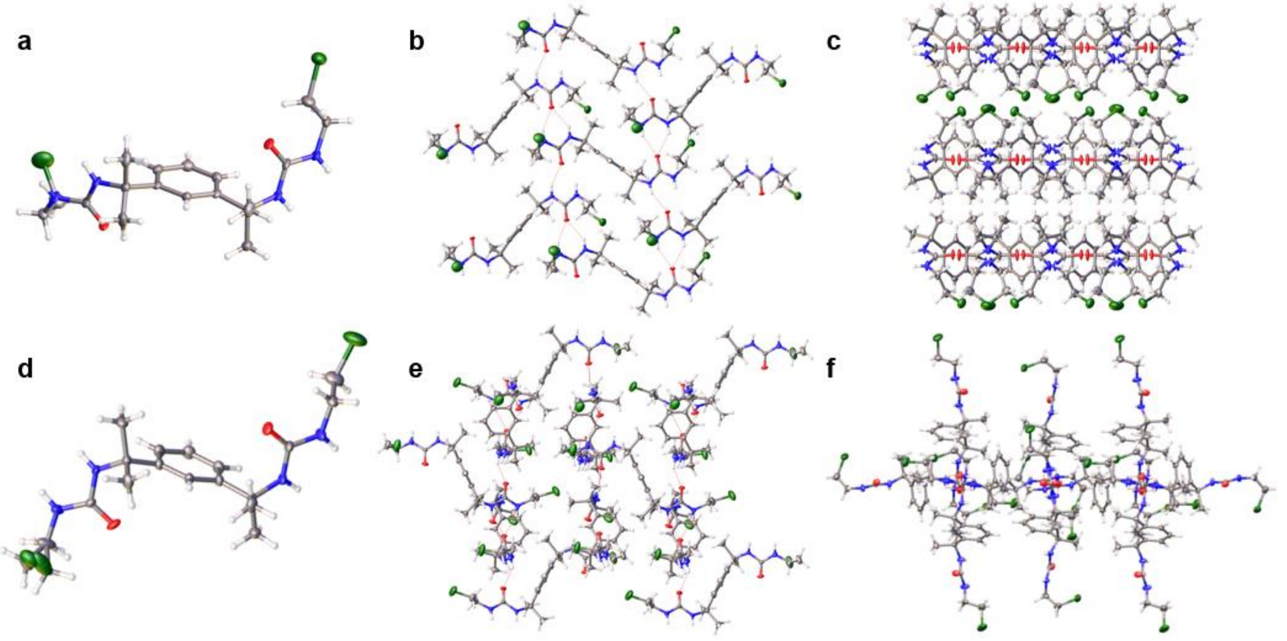

Fig. S21 (a) SCXRD geometry of **4a** in crystals of Form I; (b) urea tape network in layers of Form I, which lie parallel to the *a*-*b* plane and comprise a “brick-wall” [AB] repeat unit;[13] (c) layering of two-dimensional urea tape networks in Form I, viewed along (100); (d) SCXRD geometry of **4a** in crystals of Form II, illustrating the disorder in one chloroethyl end-group (modeled over two sites with equal occupancies); (e) urea tape network in Form II, which exhibits a three-dimensional [ABCD] hydrogen bonding topology; (f) orientation of molecules around the urea tape axes of Form II, which lie parallel to (001).

4 Variable-temperature NMR spectroscopy

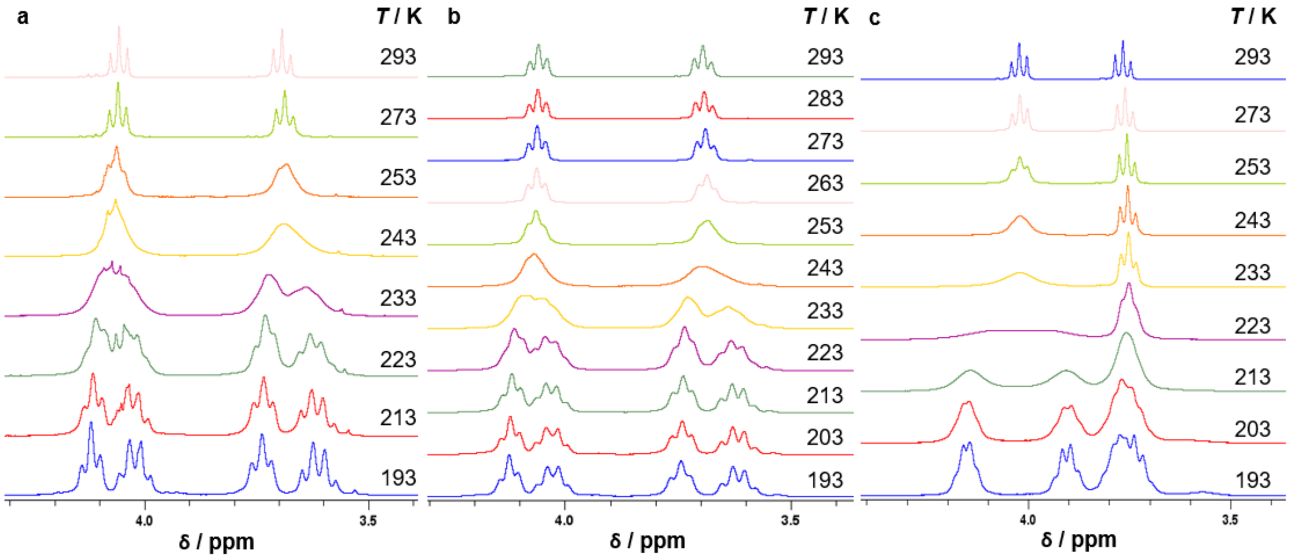

Fig. S22 ^{1}H NMR spectra (a) **1**, (b) a repeat sample of **1** and (c) **2** in dichloromethane-d_2 at varying temperatures, *T*. Spectra were recorded upon lowering the temperature, and peak positions calibrated to the reference dichloromethane-d_2 peak at δ = 5.355 ppm.

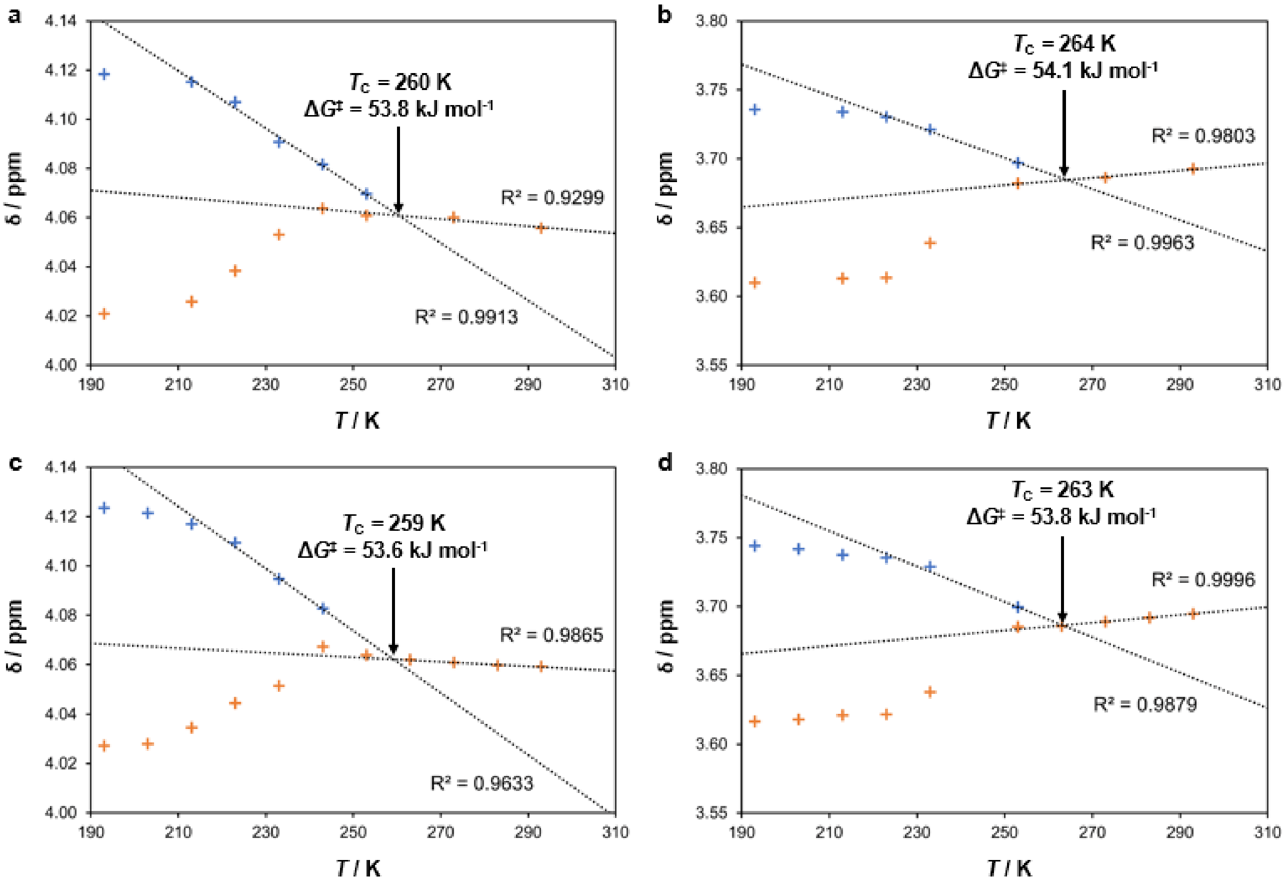

Fig. S23 ^{1}H NMR chemical shifts, δ, of the (a) α and (b) β oxazolidine CH_2 environments in **1** for a dichloromethane-d_2 solution of the compound at 190-290 K. Peak positions are calibrated to the reference dichloromethane-d_2 peak at δ = 5.355 ppm. Data for the (c) α and (d) β environments of a replicate sample are also shown, illustrating the reproducibility of the variable-temperature measurements. For each plot, the interpolated coalescence temperature, T_C, is labelled, in addition to the estimated activation energy for the conformational transition, Δ*G*‡.

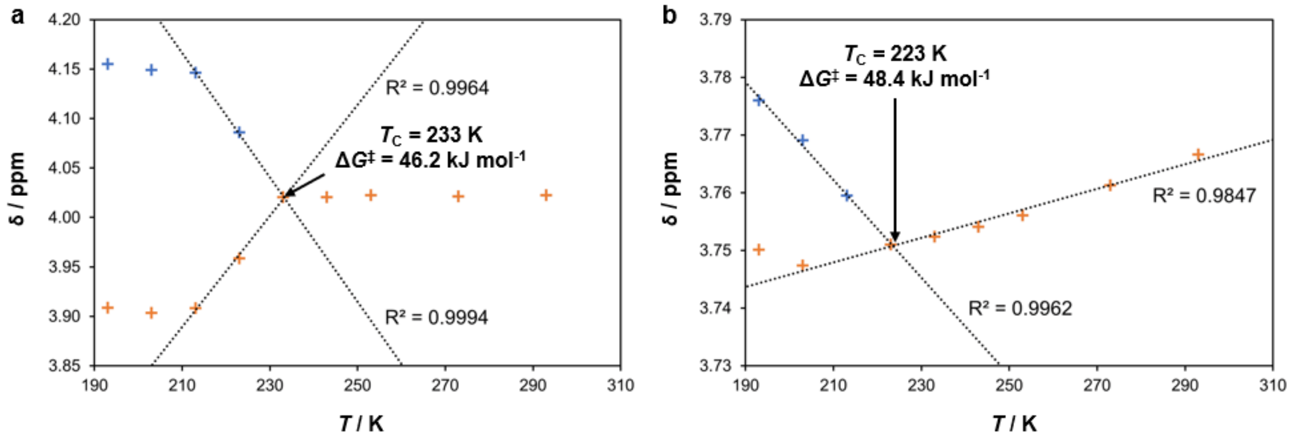

Fig. S24 ^{1}H NMR chemical shifts, δ, of the (a) α and (b) β oxazolidine CH_2 environments in **2** for a dichloromethane-d_2 solution of the compound at 190-290 K. Peak positions are calibrated to the reference dichloromethane-d_2 peak at δ = 5.355 ppm. For each plot, the interpolated coalescence temperature, T_C, is labelled, in addition to the estimated activation energy for the conformational transition, $\Delta G^‡$.

Compound	Proton	Δδ / ppm	k_r / s^{-1}	T_C / K	$\Delta G^‡$ / kJ mol^{-1}	Mean $\Delta G^‡$ / kJ mol^{-1}
**1**	α	0.098	86.6	260	53.8 ± 1.1	54 ± 1
	β	0.126	112	264	54.1 ± 1.1	
**1**	α	0.096	85.5	259	53.6 ± 1.1	54 ± 1
	β	0.128	113	263	53.8 ± 1.1	
**2**	α	0.246	219	233	46.2 ± 1.0	47 ± 1
	β	0.026	23.0	223	48.4 ± 1.1	

Table S3 Measured peak splitting parameters (Δδ), coalescence temperatures (T_C) and transition rate constants (k_r) for dichloromethane-d_2 solutions of **1** and **2**, and the resulting activation energy estimates for *syn-anti* interconversions ($\Delta G^‡$). Peak positions (δ) are calibrated to the reference dichloromethane-d_2 peak at δ = 5.355 ppm. Values of Δδ are measured at the point of maximum splitting in the variable-temperature NMR plots, while k_r is calculated using an NMR frequency of 400.13 MHz. Errors in $\Delta G^‡$ are calculated assuming a conservative systematic uncertainty in T_C of ±5 K for each experiment.

5 Conformational energy calculations

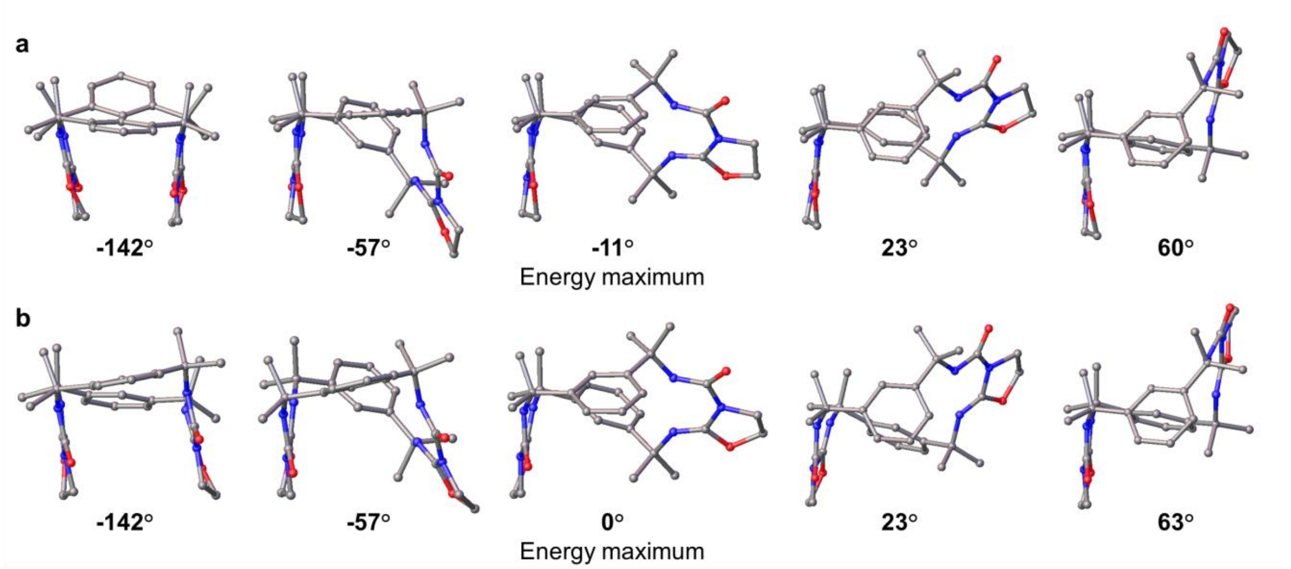

Fig. S25 Changes in the geometry of (a) *syn*-**1** and (b) *syn*-**2** as one methyl-imine torsion angle (φ) is increased. Values of φ for each optimized geometry are shown in bold and hydrogen atoms are omitted for clarity. Geometries were optimized in the basis set 6-31+G* using the DFT method B3LYP with no dispersion correction. The modeling reveals that the *syn* → *anti* transitions for the two macrocycles are mechanistically similar.

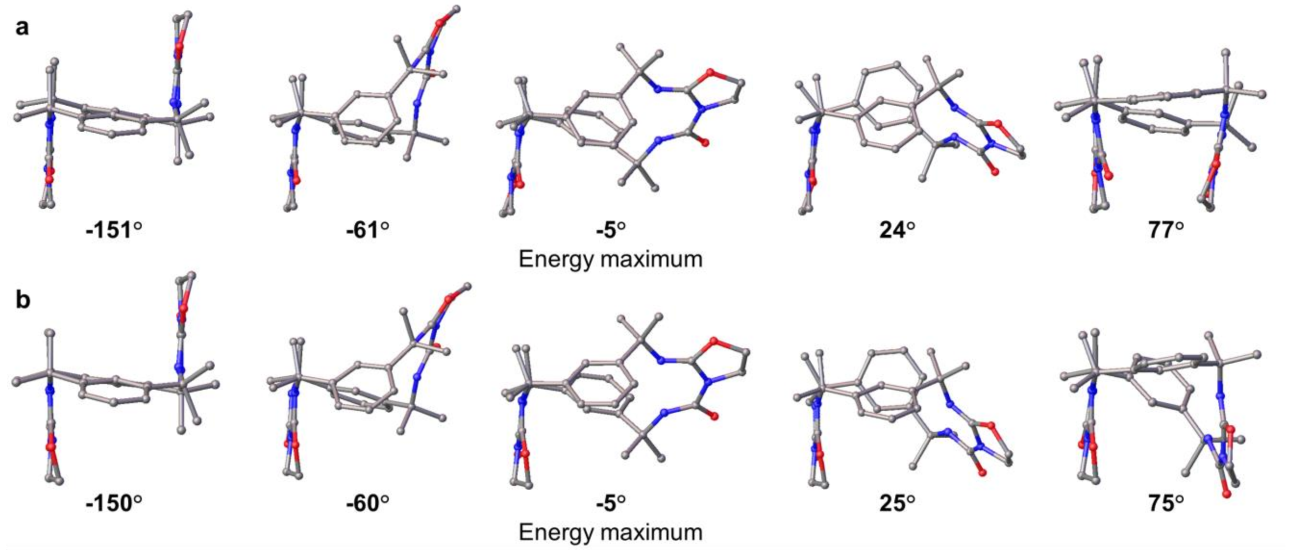

Fig. S26 Changes in the geometry of (a) *anti*-**1** and (b) *anti*-**2** as one methyl-imine torsion angle (φ) is increased. Values of φ for each optimized geometry are shown in bold and hydrogen atoms are omitted for clarity. Geometries were optimized in the basis set 6-31+G* using the DFT method B3LYP with no dispersion correction. The modeling reveals that the *anti* → *syn* transitions for the two macrocycles are mechanistically similar.

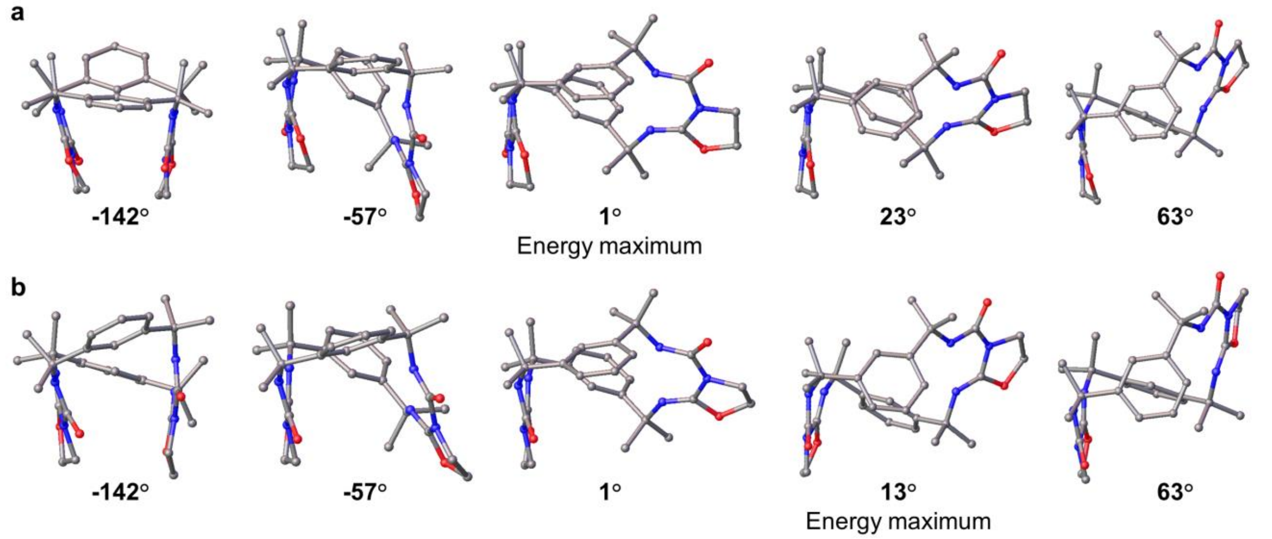

Fig. S27 Changes in the geometry of (a) *syn*-**1** and (b) *syn*-**2** as one methyl-imine torsion angle (φ) is increased. Values of φ for each optimized geometry are shown in bold and hydrogen atoms are omitted for clarity. Geometries were optimized in the basis set 6-31+G* using the DFT method B3LYP with the D3BJ dispersion correction. The modeling reveals that the *syn* → *anti* transitions for the two macrocycles are mechanistically similar.

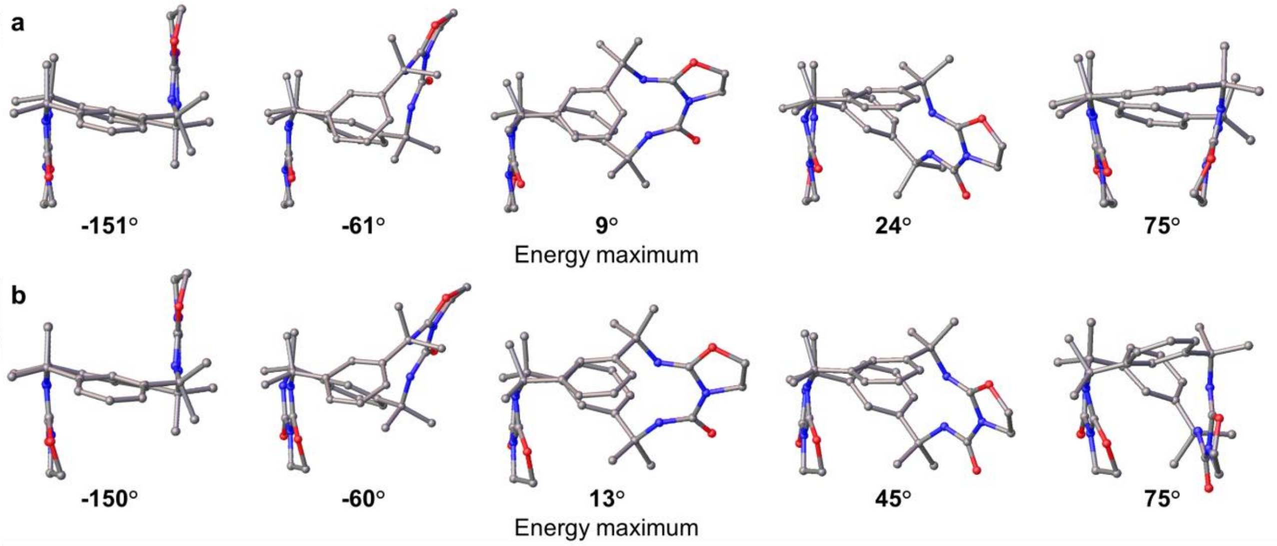

Fig. S28 Changes in the geometry of (a) *anti*-**1** and (b) *anti*-**2** as one methyl-imine torsion angle (φ) is increased. Values of φ for each optimized geometry are shown in bold and hydrogen atoms are omitted for clarity. Geometries were optimized in the basis set 6-31+G* using the DFT method B3LYP with the D3BJ dispersion correction. The modeling reveals that the *anti* → *syn* transitions for the two macrocycles are mechanistically similar.

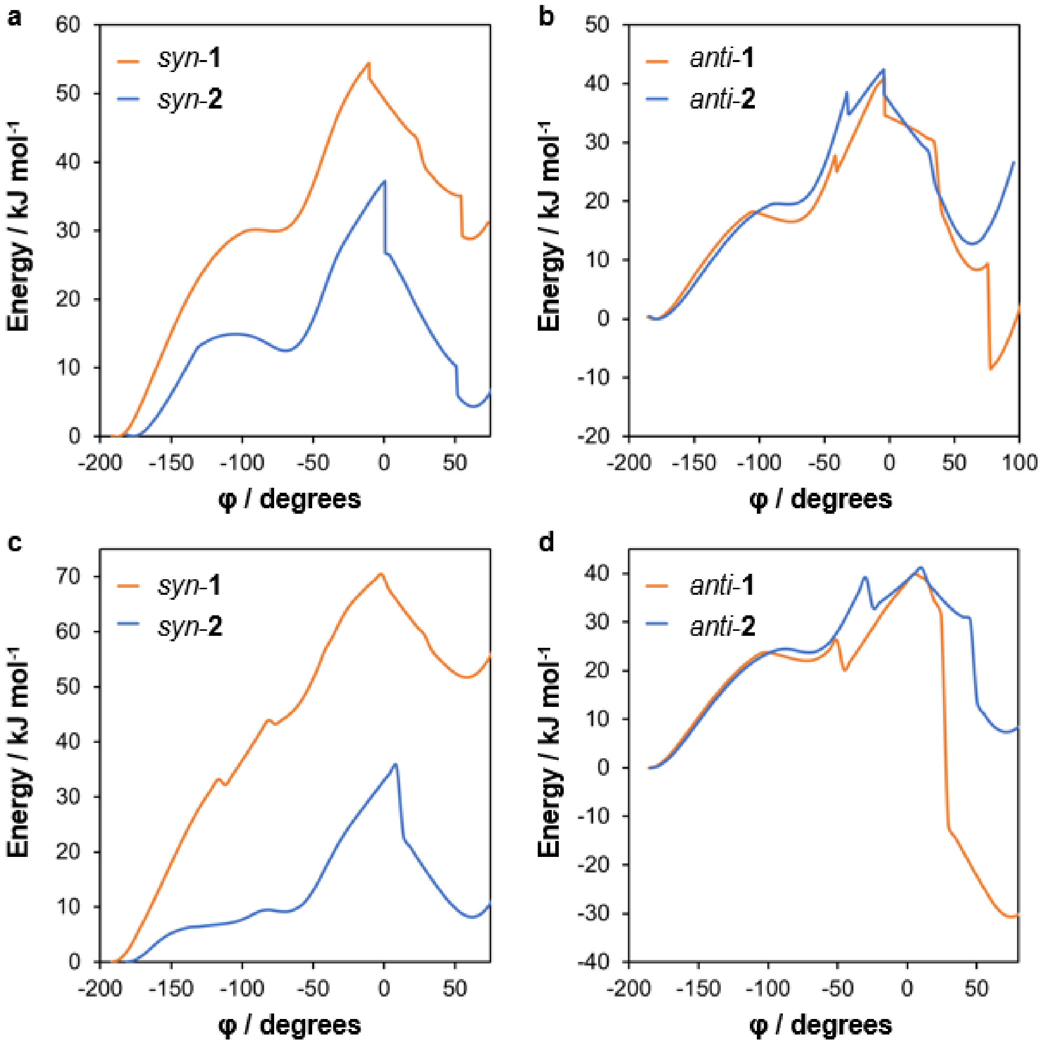

Fig. S29 Changes in energy of macrocycles **1** and **2** for increasing methyl-imine torsion angles (φ), beginning with the DFT-optimized (a) *syn* and (b) *anti* conformations. The macrocycle geometries were optimized after each scan step (keeping φ fixed) using the DFT method B3LYP in the basis set 6-31+G* with no dispersion correction. Repeating the scans with the dispersion correction D3BJ produced the plots shown in (c) and (d). Only small changes are observed in the energy landscape of **2**, whereas the barrier for the transition of *syn*-**1** to *anti*-**1** is greatly increased.

Method	Compound	Energy difference (*syn-anti*) / kJ mol^{-1}		
		6-31++G**	def2-TZVP	aug-cc-PVDZ
B3LYP	**1**	-13.29	-12.09	-13.46
B3LYP-D3BJ	**1**	-31.39	-29.14	-30.73
B3LYP	**2**	6.24	6.32	5.43
B3LYP-D3BJ	**2**	6.53	1.83	0.77

Table S4 Energy differences between the *syn* and *anti* conformers of **1** and **2**, calculated in a range of basis sets via the DFT method B3LYP with and without the D3BJ dispersion correction. The geometry of each molecule was optimized in the selected basis set after an initial optimization in the smaller basis set 6-31+G*. Negative energy values correspond to systems in which the *syn* conformer is more stable.

Method	Compound	Basis set	Activation energy for *syn-anti* transition / kJ mol^{-1}		
			Initial structure *syn*	Initial structure *anti*	Mean
B3LYP	**1**	6-31++G**<br>def2-TZVP<br>aug-cc-PVDZ<br>**Mean**	48.02<br>53.86<br>54.79<br>57.0 ± 2.6	54.81<br>47.68<br>55.25<br>52.6 ± 3.0	51.4 ± 3.4<br>50.8 ± 3.1<br>55.0 ± 0.2<br>**52.4 ± 1.6**
B3LYP-D3BJ	**1**	6-31++G**<br>def2-TZVP<br>aug-cc-PVDZ<br>**Mean**	71.22<br>68.02<br>69.81<br>69.7 ± 1.1	70.28<br>69.86<br>69.64<br>69.9 ± 0.2	70.8 ± 0.5<br>68.9 ± 0.9<br>69.7 ± 0.1<br>**69.8 ± 0.6**
B3LYP	**2**	6-31++G**<br>def2-TZVP<br>aug-cc-PVDZ<br>**Mean**	38.28<br>33.25<br>43.13<br>39.2 ± 3.5	43.26<br>39.43<br>39.10<br>40.6 ± 1.6	40.8 ± 2.5<br>36.3 ± 3.1<br>41.1 ± 2.0<br>**39.4 ± 1.9**
B3LYP-D3BJ	**2**	6-31++G**<br>def2-TZVP<br>aug-cc-PVDZ<br>**Mean**	42.21<br>43.47<br>41.91<br>42.5 ± 0.6	38.25<br>39.21<br>37.53<br>38.3 ± 0.6	40.2 ± 2.0<br>41.3 ± 2.1<br>39.7 ± 2.2<br>**40.4 ± 0.6**

Table S5 Energy barriers for interconversion of the *syn* and *anti* conformers of **1** and **2**, calculated via the DFT method B3LYP with and without the dispersion correction. The conformational change was simulated by varying the torsion angle between one oxazolidine ring and its *anti* methyl group and re-optimizing the structure after each scan step. Torsions were incremented in steps of 0.2° near the energetic maximum and 1-5° elsewhere, depending on the smoothness of the geometric changes. Geometry optimizations were performed in the basis set 6-31+G*, and the highest-energy geometry refined in a range of larger basis sets while fixing the scanned torsion angle. Mean activation energies (most reliable in bold) correspond to the difference between the most stable macrocycle conformer (*syn*-**1** or *anti*-**2**) and the maximum-energy geometry, which was calculated for both the *syn* → *anti* and *anti* → *syn* transitions. Errors in the mean values for each basis set are equal to half the difference between the two calculations. All other errors correspond to standard errors in the mean values.

Compound	Conformational energy / kJ mol^{-1}		
	Maximum	Mean	Standard deviation
**6**	25.4	13.3	5.3
**7**	9.5	4.1	1.8

Table S6 Maximum and mean and standard deviation values extracted from the conformational energy landscapes of **6** and **7**. Conformational energies were calculated by optimizing the geometries of the molecules for different combinations of torsion angles ϕ_1 and ϕ_2, spanning the full range of possibilities $0 \leq \phi_1 < 360°$ and $0 \leq \phi_2 \leq 180°$. The final conformational landscapes were constructed by performing eight replicate calculations with different initial molecular conformations and averaging the results.

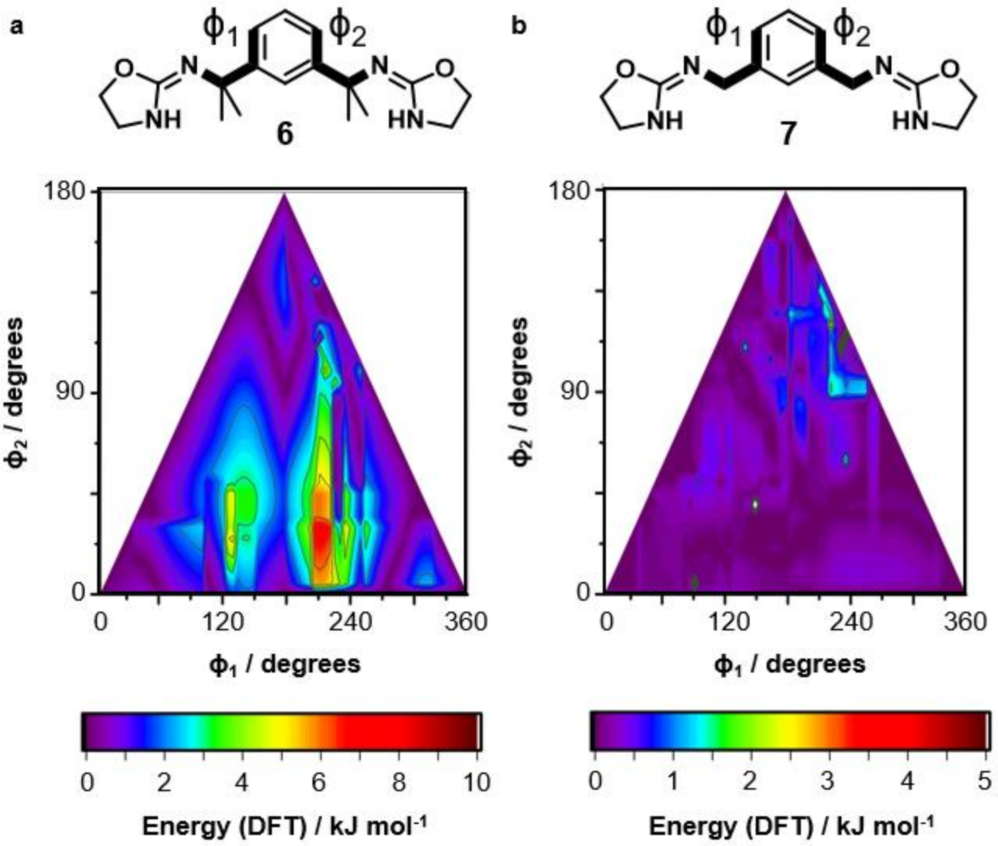

Fig. S30 Convergence tests for the conformational energy landscapes of (a) **6** and (b) its theoretical non-methylated analogue **7**. Mean energy values were calculated from eight replicate analyses with different initial combinations of the torsion angles ϕ_1 and ϕ_2. Illustrated in the contour plots are differences in energy for pairs of symmetry-equivalent combinations of ϕ_1 and ϕ_2. Small differences (<2 kJ mol^{-1}) are indicative of consistency in the DFT calculations and convergence of the mean results. The mean differences in (a) and (b) are 1.5 and 0.23 kJ mol^{-1}, respectively, suggesting that most areas of the energy landscapes have reached convergence.

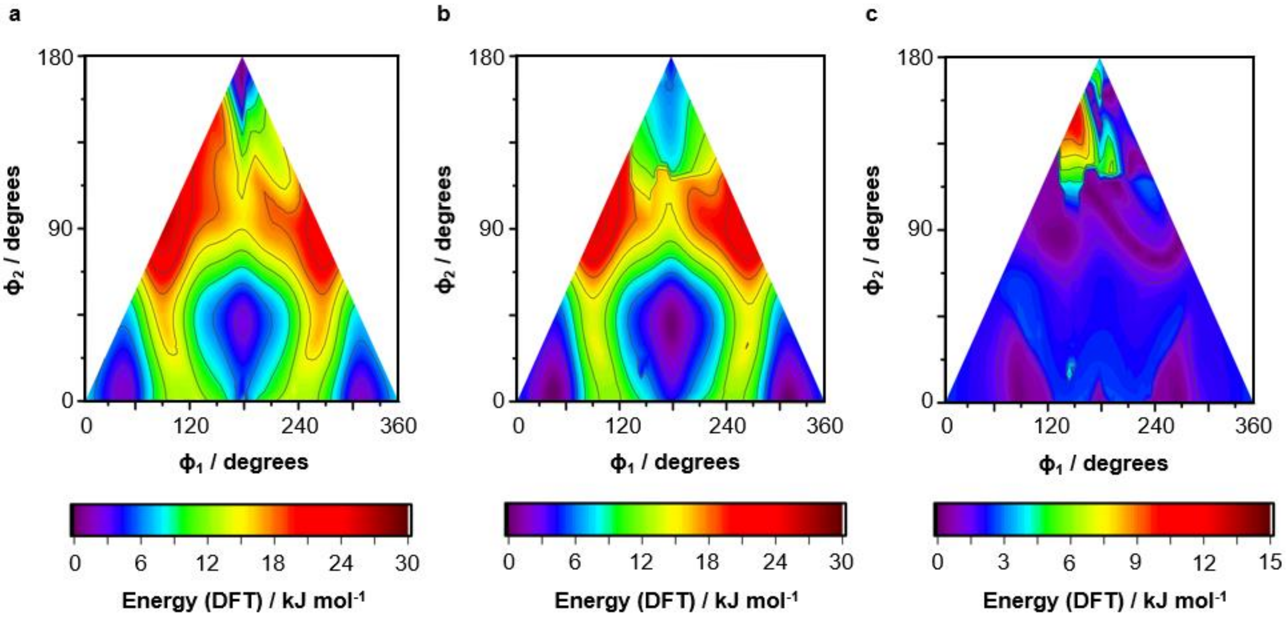

Fig. S31 Conformational energy landscapes of **6** calculated using the DFT method B3LYP/6-31+G* (a) with and (b) without the D3BJ correction for dispersion forces, and (c) the absolute differences between these alternative results. Calculations were performed once for each combination of torsion angles ϕ_1 and ϕ_2 using the same initial molecular conformations. Applying the correction slightly increases the separation of peaks and troughs and raises the energies of conformations near $\phi_1 = \phi_2 = 180°$, although the latter discrepancy is largely eliminated with further repeats (see Fig. 10). The qualitative appearance of the plot, the positions of the peaks and troughs and the majority of energies are only weakly affected. The maximum conformational energies in (a) and (b) are 25.8 and 24.8 kJ mol^{-1}, respectively, while the mean energies are 11.6 and 10.0 kJ mol^{-1} and the standard deviations 5.4 and 5.7 kJ mol^{-1}. The mean difference between equivalent combinations of ϕ_1 and ϕ_2 is 1.9 kJ mol^{-1}, with a standard deviation of 1.4 kJ mol^{-1}.

6 Batch synthesis

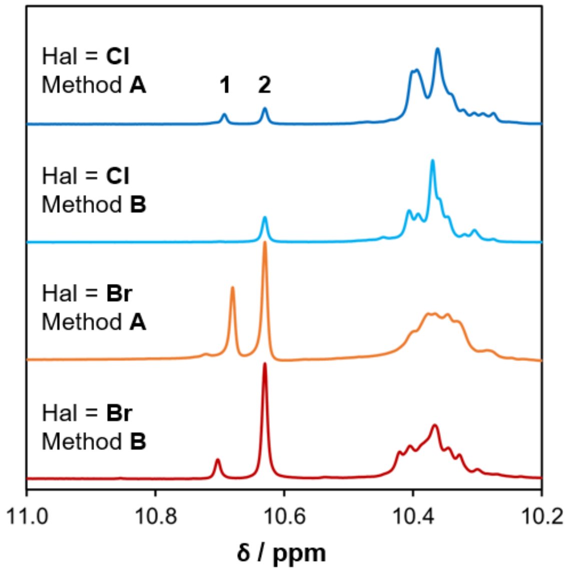

Fig. S32 Representative ^{1}H NMR spectra of the crude products of batch syntheses performed with different methods and starting materials at room temperature. The intensities of the macrocycle NH signals (labelled in bold) are scaled by concentration via normalization against an acetonitrile internal standard. For ease of comparison, the NH signal of **2** is shifted to a fixed position of δ = 10.63 ppm in all experiments. The use of 2-bromoethylamine greatly increases the total conversion but with lower selectivity for product **2**. Selectivities are also higher when Method B is used.

Method	Hal	Sample	Conversion (total) / %	Conversion (2) / %	Selectivity / %
		1	1.99	1.10	55.5
		2	1.75	1.01	57.3
A	Cl	3	1.68	1.00	59.3
		4	1.48	0.97	65.6
		**Mean**	**1.7 ± 0.2**	**1.02 ± 0.06**	**59 ± 4**
		1	2.46	2.46	100
		2	2.05	2.05	100
B	Cl	3	2.15	2.12	98.3
		4	2.71	2.65	98.0
		**Mean**	**2.3 ± 0.3**	**2.3 ± 0.3**	**99 ± 1**
		1	20.6	12.2	59.1
		2	19.1	11.9	62.4
A	Br	3	23.1	14.9	64.7
		4	23.1	13.5	58.6
		**Mean**	**21.4 ± 2.0**	**13.1 ± 1.4**	**61 ± 3**
		1	18.7	15.5	82.7
		2	14.2	12.3	87.1
B	Br	3	14.5	13.3	91.6
		4	14.3	12.5	87.5
		**Mean**	**15.4 ± 2.2**	**13.4 ± 1.5**	**87 ± 4**

Table S7 Conversions and selectivities of replicate macrocycle syntheses performed using Methods A and B. Values were obtained through ^{1}H NMR analysis of the crude products after quenching with methanol, removal of the solvent *in vacuo* and dissolution of the residue in $CDCl_3$. Selectivities for **2** were calculated from the relative integrals of the macrocycle NH signals, while conversions were estimated by comparison with the CH_3 integral of an acetonitrile internal standard. Errors are equal to the standard deviations of the replicate results.

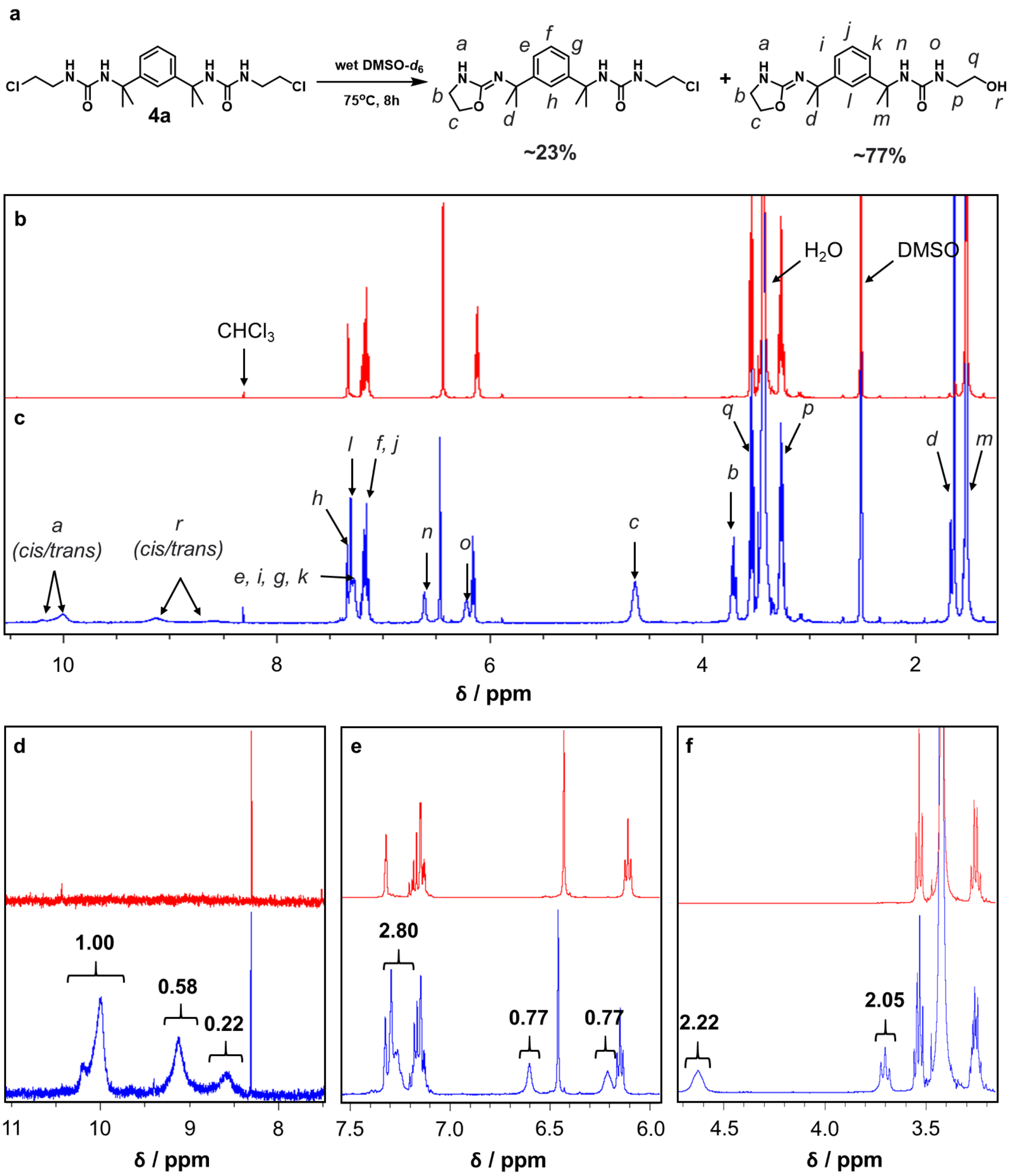

Fig. S33 (a) One possible scheme of oxazolidine formation from **4a** in DMSO-d_6, deduced from tentative NMR assignments; (b) ^{1}H NMR spectrum of **4a** (60 mM) in wet DMSO-d_6 before heating; (c) ^{1}H NMR spectrum of the same solution of **4a** after heating and stirring at 75°C for 8 hours, with new signals marked by their proposed assignments; (d) comparison of the NH and OH signals of **4a** (red line) and the proposed oxazolidine products (blue), with the relative integrals of unique product signals labelled. We hypothesize that the new signals correspond to the oxazolidine NH groups in the two geometric (*cis*/*trans*) isomers of the product (each potentially existing in two rapidly equilibrating tautomeric forms[14]) and downfield alcohol groups of their hydrolysates. The positions of the OH signals may differ between geometric isomers due to intramolecular hydrogen bonding; (e) aryl CH and urea signals of the reactant and product; (f) alkyl CH signals of the reactant and product. The relative integral of the new signal at δ = 1.65 ppm is measured as 5.74, approximately matching the expected integrals of CH_3 protons *d* (6.00).

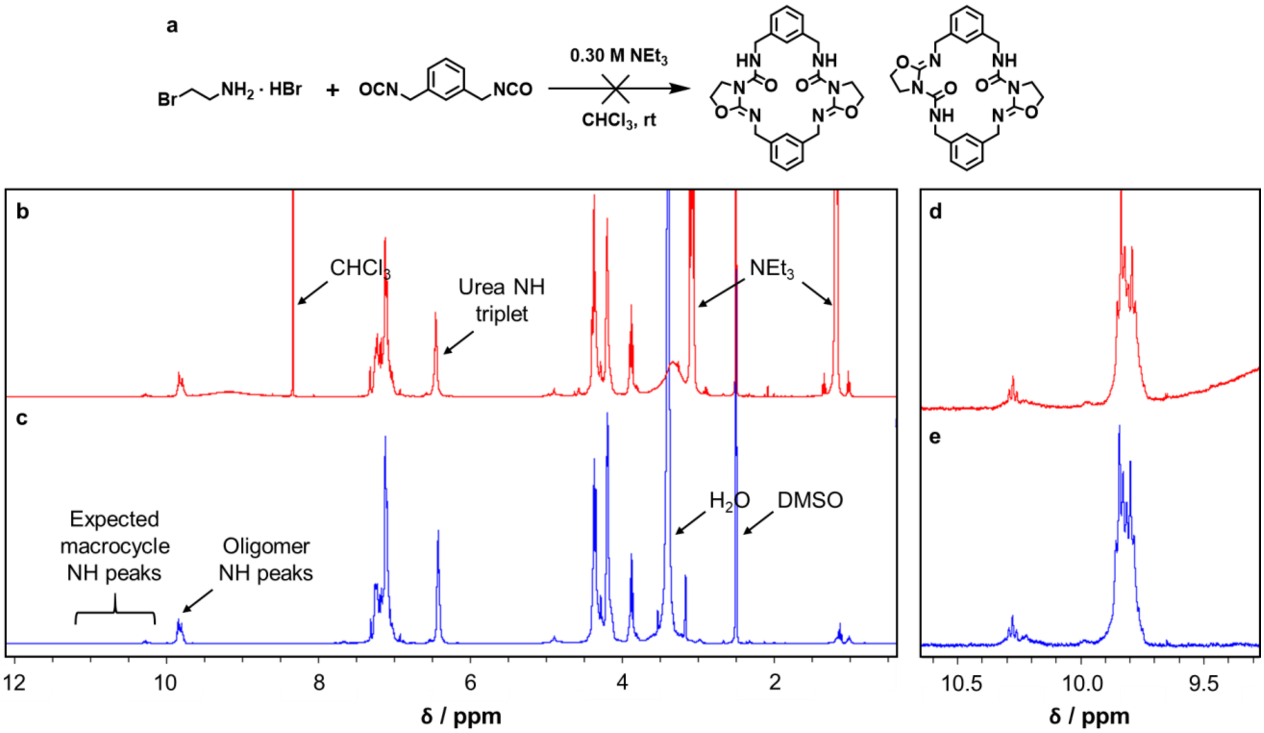

Fig. S34 (a) Attempted macrocycle synthesis from 2-bromoethylamine hydrobromide and *m*-xylylene diisocyanate, performed in a stirred chloroform solution of triethylamine (0.300 M, 10 mL) over 24 hours at room temperature. The solution of 2-bromoethylamine (0.145 M) was added to neat isocyanate (0.5 eq.) and further neat isocyanate (0.5 eq.) was added after 3 hours, resulting in a yellow solution. Rotary evaporation of the reaction mixture produced an off-white solid, which could not be redissolved in triethylamine/chloroform with heating; (b) ^{1}H NMR spectrum of the crude product in DMSO-d_6 after rotary evaporation; (c) ^{1}H NMR spectrum of the solid product (318 mg) after washing with chloroform (10 mL); (d) downfield NH signals of the crude product before washing with chloroform; (e) downfield NH signals of the product after washing with chloroform. Note that the weak triplets in the region δ = 9.5-10.5 ppm were not affected by the chloroform wash, so are unlikely to correspond to the urea NH groups of the target macrocycles.

7 Kinetic studies

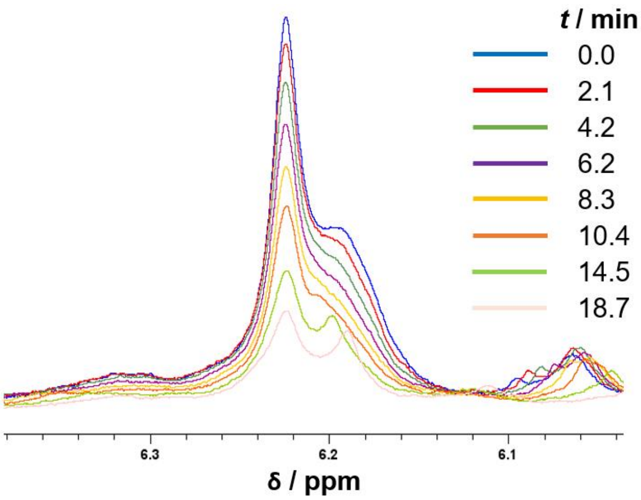

Fig. S35 ^{1}H NMR spectra measured during the reaction of 2-bromoethylamine hydrobromide with tetramethylxylylene diisocyanate in triethylamine/chloroform at 21°C. Spectra are normalized against the integral of the triethylamine CH_2 (quartet, δ = 2.95 ppm) and CH_3 (triplet, δ = 1.30 ppm) signals. For clarity, each spectrum is shifted to coincide with the peak positions of the initial spectrum.

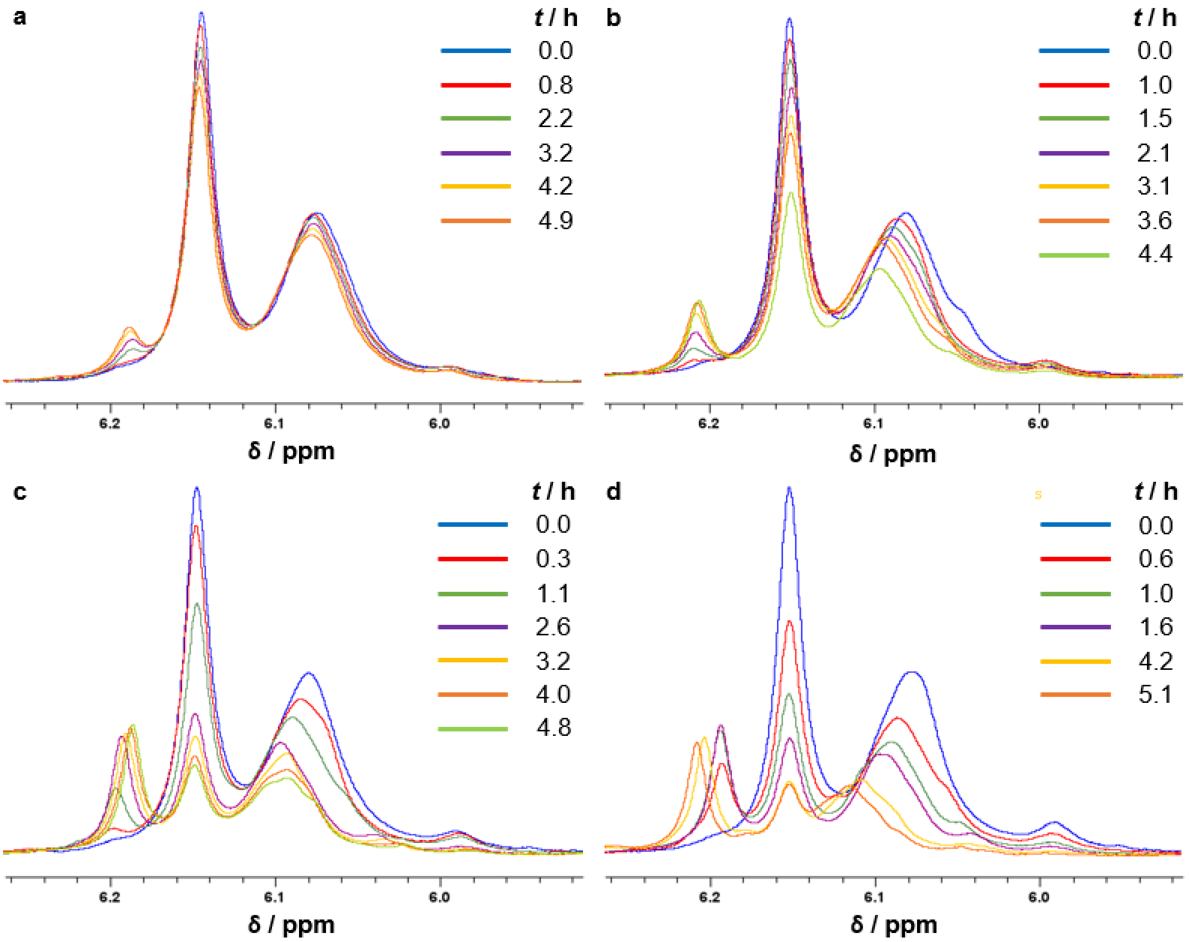

Fig. S36 ^{1}H NMR spectra measured during the reaction of 2-chloroethylamine hydrochloride with tetramethylxylylene diisocyanate in triethylamine/chloroform at (a) 21°C, (b) 30°C, (c) 40°C and (d) 50°C. Spectra are normalized against the integral of the triethylamine CH_2 (quartet, δ = 2.95 ppm) and CH_3 (triplet, δ = 1.30 ppm) signals. For clarity, each spectrum is shifted to coincide with the peak positions of the initial spectrum.

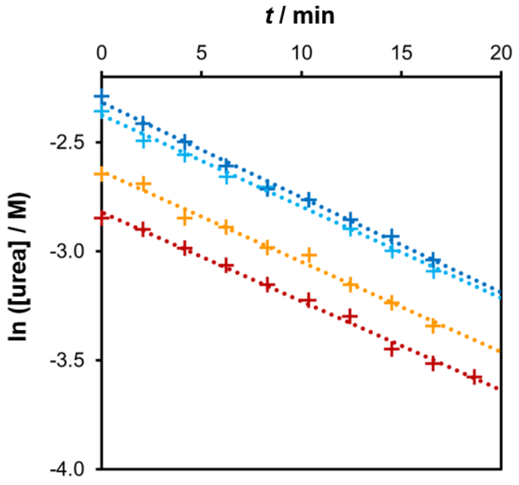

Fig. S37 First-order kinetic plots for replicate reactions of 2-bromoethylamine hydrobromide with tetramethylxylylene diisocyanate in a 0.3 M solution of triethylamine in chloroform at 21°C. The reaction time *t* is measured from the start of the NMR experiment rather than the mixing of the reagents, as the variable effect of agitation on the initial value of [urea] prevents meaningful extrapolation of the plotted trend lines.

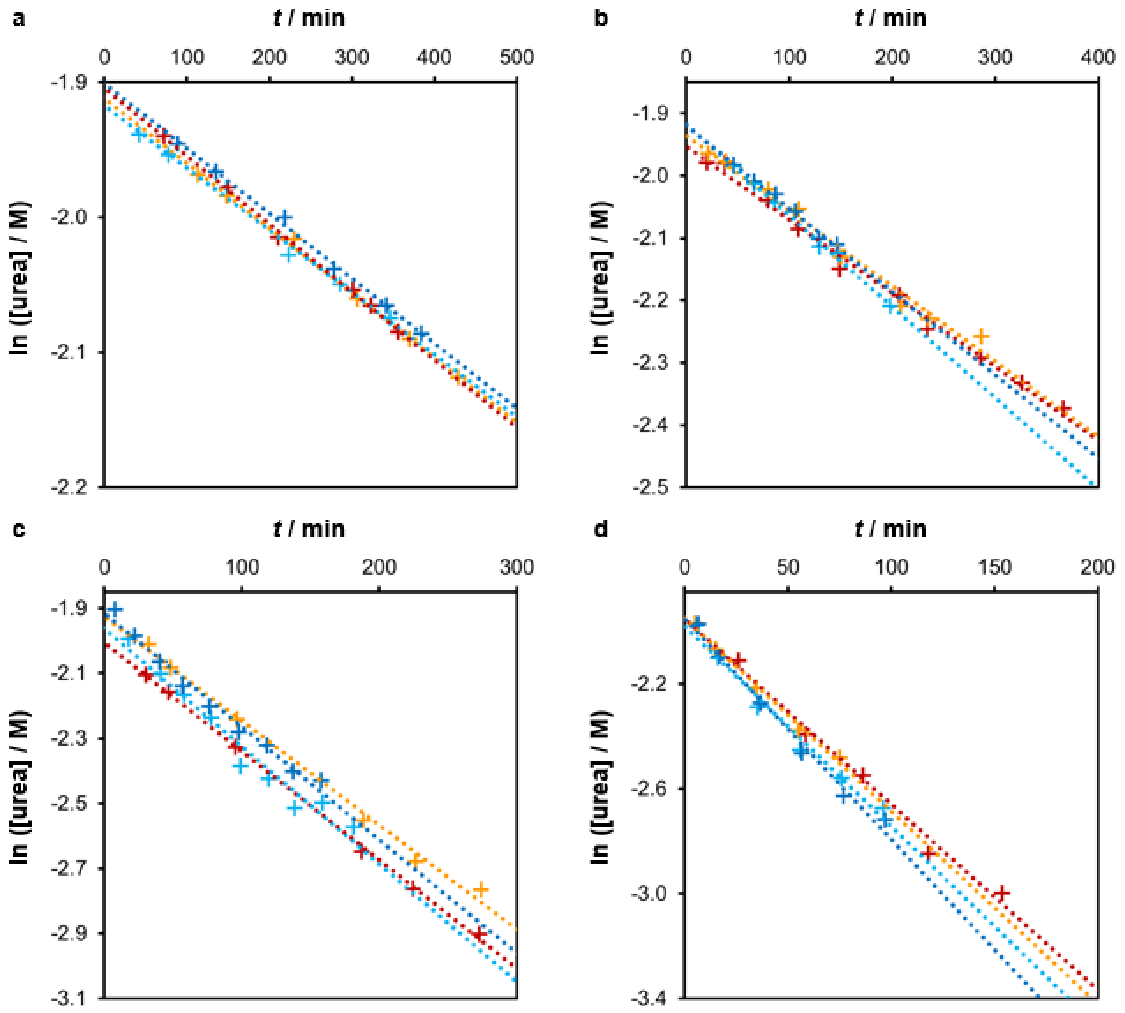

Fig. S38 First-order kinetic plots for replicate reactions of 2-chloroethylamine hydrochloride with tetramethylxylylene diisocyanate in a 0.3 M solution of triethylamine in chloroform at (a) 21°C, (b) 30°C, (c) 40°C and (d) 50°C. The reaction time *t* is measured from the point at which reagents were mixed.

T / °C	Hal	Measured mean T / °C	Sample	k / s^{-1}	R^2 of first-order plot	Mean ln(k / s^{-1})
21	Br	21.0 ± 0.5	1<br>2<br>3<br>4<br>**Mean**	(6.80 ± 0.19) x 10^{-4}<br>(6.91 ± 0.29) x 10^{-4}<br>(7.00 ± 0.27) x 10^{-4}<br>(7.27 ± 0.21) x 10^{-4}<br>**(7.0 ± 0.1) x 10^{-4}**	0.994 (n = 10)<br>0.988 (n = 9)<br>0.990 (n = 9)<br>0.994 (n = 9)	**-7.27 ± 0.02**
21	Cl	21.0 ± 0.5	1<br>2<br>3<br>4<br>**Mean**	(8.38 ± 0.20) x 10^{-6}<br>(8.00 ± 0.21) x 10^{-6}<br>(7.67 ± 0.35) x 10^{-6}<br>(8.02 ± 0.22) x 10^{-6}<br>**(8.0 ± 0.2) x 10^{-6}**	0.998 (n = 6)<br>0.997 (n = 6)<br>0.992 (n = 6)<br>0.997 (n = 6)	**-11.73 ± 0.02**
30	Cl	30.0 ± 0.9	1<br>2<br>3<br>4<br>**Mean**	(1.97 ± 0.06) x 10^{-5}<br>(2.02 ± 0.11) x 10^{-5}<br>(2.42 ± 0.08) x 10^{-5}<br>(2.22 ± 0.12) x 10^{-5}<br>**(2.2 ± 0.1) x 10^{-5}**	0.993 (n = 10)<br>0.982 (n = 8)<br>0.996 (n = 6)<br>0.989 (n = 6)	**-10.74 ± 0.06**
40	Cl	40.1 ± 0.8	1<br>2<br>3<br>4<br>**Mean**	(5.56 ± 0.09) x 10^{-5}<br>(5.34 ± 0.21) x 10^{-5}<br>(6.03 ± 0.45) x 10^{-5}<br>(5.80 ± 0.30) x 10^{-5}<br>**(5.7 ± 0.2) x 10^{-5}**	0.999 (n = 6)<br>0.994 (n = 6)<br>0.963 (n = 9)<br>0.982 (n = 9)	**-9.78 ± 0.03**
50	Cl	49.6 ± 0.5	1<br>2<br>3<br>4<br>**Mean**	(1.18 ± 0.08) x 10^{-4}<br>(1.23 ± 0.07) x 10^{-4}<br>(1.29 ± 0.17) x 10^{-4}<br>(1.44 ± 0.12) x 10^{-4}<br>**(1.3 ± 0.1) x 10^{-4}**	0.987 (n = 5)<br>0.992 (n = 5)<br>0.937 (n = 6)<br>0.972 (n = 6)	**-8.96 ± 0.05**

Table S8 Measured temperatures and rate constants for replicate reactions of 2-chloro and 2-bromoethylamine with tetramethylxylylene diisocyanate. Conversions were measured from changing integrals of urea signals in ^{1}H NMR spectra of the reaction mixtures, with the CH_2 and CH_3 integrals of triethylamine used as normalization factors. Rate constants were calculated from the gradients of first-order kinetic plots, for which the correlation coefficients (R^2) and number of fitted points (n) are shown. Values of ln(k) were used to construct Arrhenius plot, yielding an estimate for the activation energy (E_a) of the reaction with 2-chloroethylamine. Uncertainties in temperature correspond to the standard deviations for the aggregated experiments, while all other uncertainties correspond to standard errors in the mean values.

Linear regression parameter	Value	Arrhenius parameter	Value
Slope / K	(-9.20 ± 0.17) x 10^3	E_a / kJ mol^{-1}	76.5 ± 1.4
Intercept	19.6 ± 0.6	A / s^{-1}	(3.6 ± 1.9) x 10^8

Table S9 Linear regression fit and derived Arrhenius parameters for the reaction of 2-chloroethylamine with tetramethylxylylene diisocyanate. It is noted that formation of the oxazolidine must be accompanied by a deprotonation step. Thus, it is probable that triethylamine participates in the rate-determining step (i.e. k is dependent on [NEt_3]), causing the reaction to exhibit a lower A value than a typical unimolecular process[15] (10^{10}-10^{13} s^{-1}).

Parameter	Mean	Upper limit	Lower limit
E_a / kJ mol^{-1}	76.46	77.91	75.02
ln(A / s^{-1})	19.56	20.12	19.00
k_{Br} / s^{-1}	6.99 x 10^{-4}	7.11 x 10^{-4}	6.88 x 10^{-4}
T_{eq} / °C	69.68	70.64	68.75

Table S10 Estimation of the temperature (T_{eq}) needed for 2-chloroethylamine to equal the reaction rate of 2-bromoethylamine at room-temperature. The value of T_{eq} is estimated by rearranging the Arrhenius relation $k_{\mathrm{Br}} = A\exp(-E_{\mathrm{a}}/\mathrm{R}T_{\mathrm{eq}})$ to obtain $T_{\mathrm{eq}} = -E_{\mathrm{a}}/(R(\ln k_{\mathrm{Br}} - \ln A))$, where E_a and A are the activation energy and pre-exponential constant of the 2-chloroethylamine reaction. To obtain conservative estimates for the uncertainty in T_{eq}, the calculation was repeated for the upper and lower bounds of the input parameters. In all cases, these bounds were estimated by raising or lowering the mean value by the corresponding standard error.

8 Semi-continuous flow synthesis

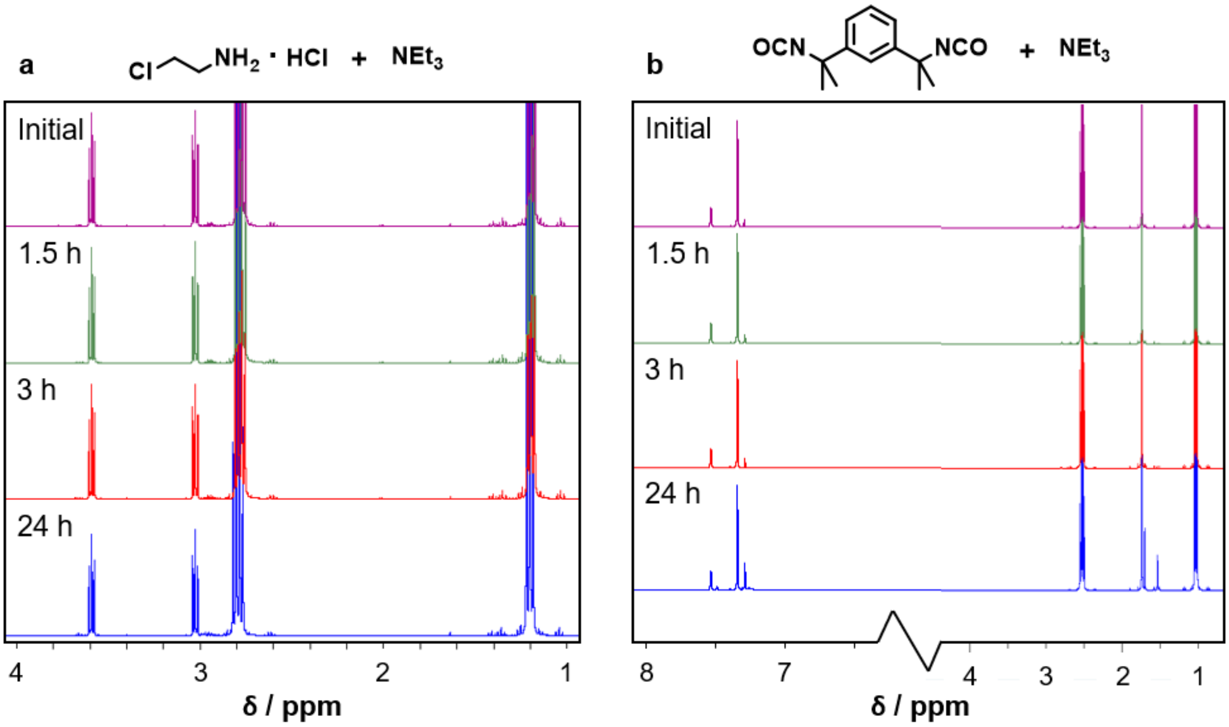

Fig. S39 ^{1}H NMR spectra of (a) 2-chloroethylamine hydrochloride (0.145 M) and (b) tetramethylxylylene diisocyanate (0.145 M) in a 0.300 M solution of triethylamine in $CDCl_3$, measured at multiple intervals over 24 hours. Comparison of the spectra reveals little alteration over the duration of the experiment.

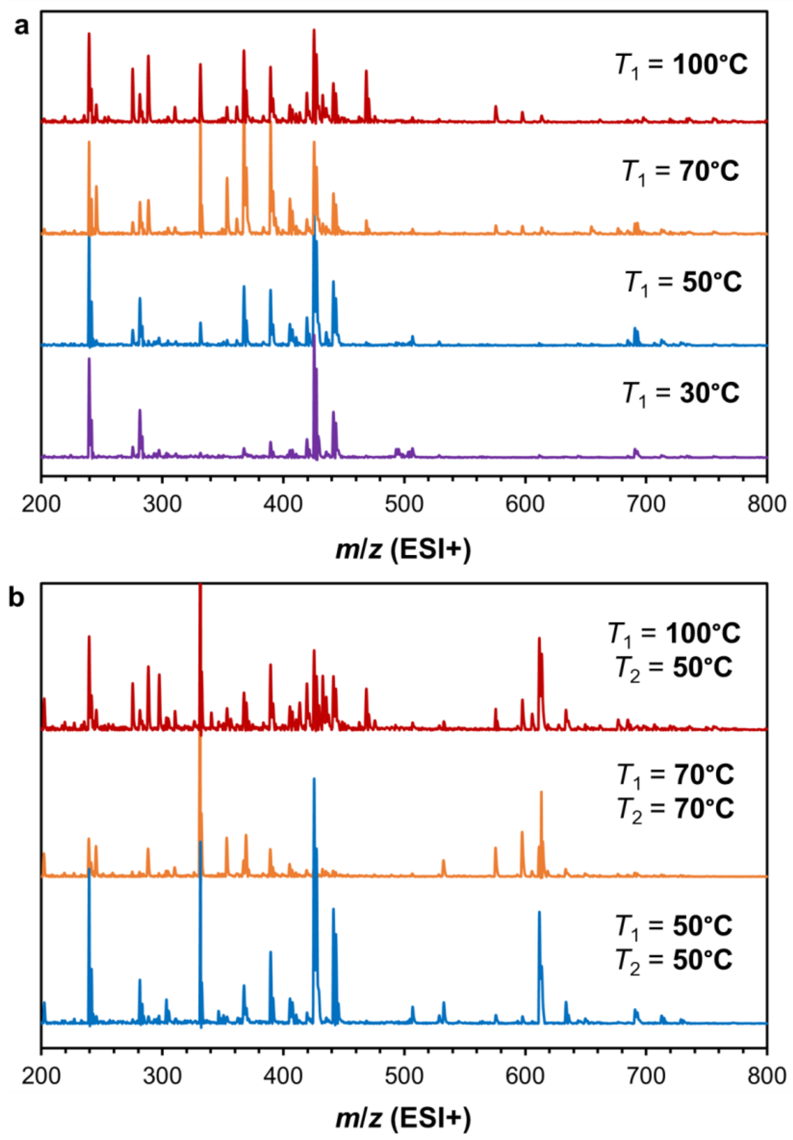

Fig. S40 At-line mass spectra for reaction mixtures sampled after (a) the first reaction step at different values of T_1 and (b) the second reaction step at different values of T_1 and T_2. Spectra were recorded in methanol doped with 0.1% (v/v) formic acid.

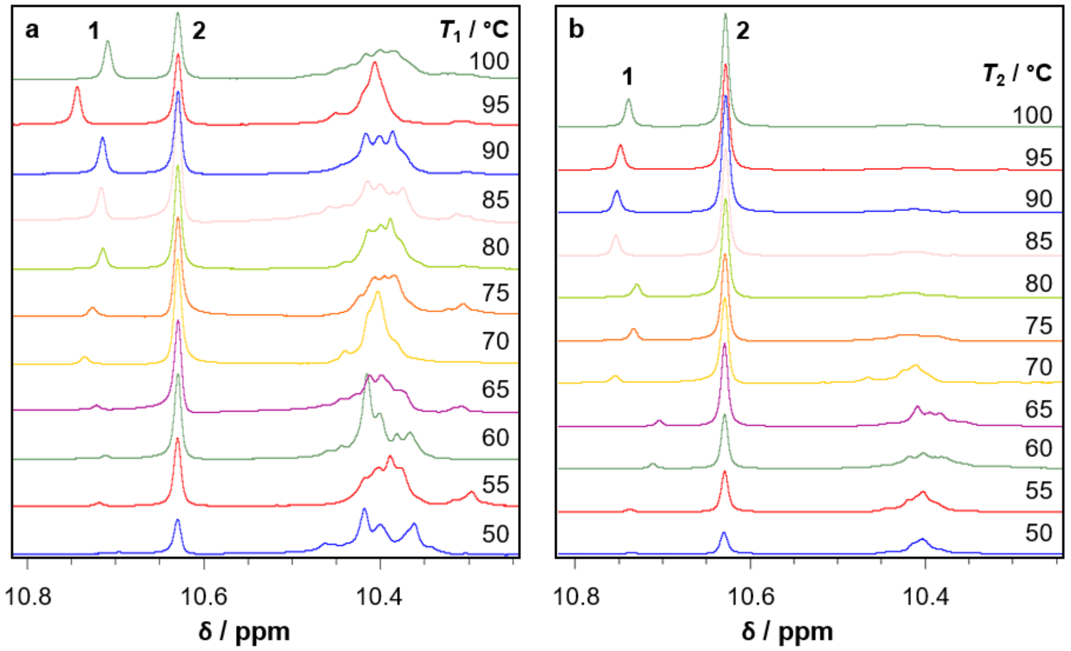

Fig. S41 ^{1}H NMR spectra of the crude products from semi-continuous flow syntheses performed at varying values of (a) T_1 with T_2 = 50°C and (b) T_2 with T_1 = 70°C. The intensities of the macrocycle NH signals (labelled in bold) are scaled by concentration via normalization against an acetonitrile internal standard. For ease of comparison, the NH signal of **2** is shifted to a fixed position of δ = 10.63 ppm in all experiments. Increasing T_1 leads to a moderate rise in conversion with substantial loss of selectivity for **2**. Increasing T_2, however, produces a sharp increase in conversion with a smaller decrease in selectivity. At the highest values of T_2, the intermediate signals around δ = 10.4 ppm are almost absent, indicating complete conversion to the target products.

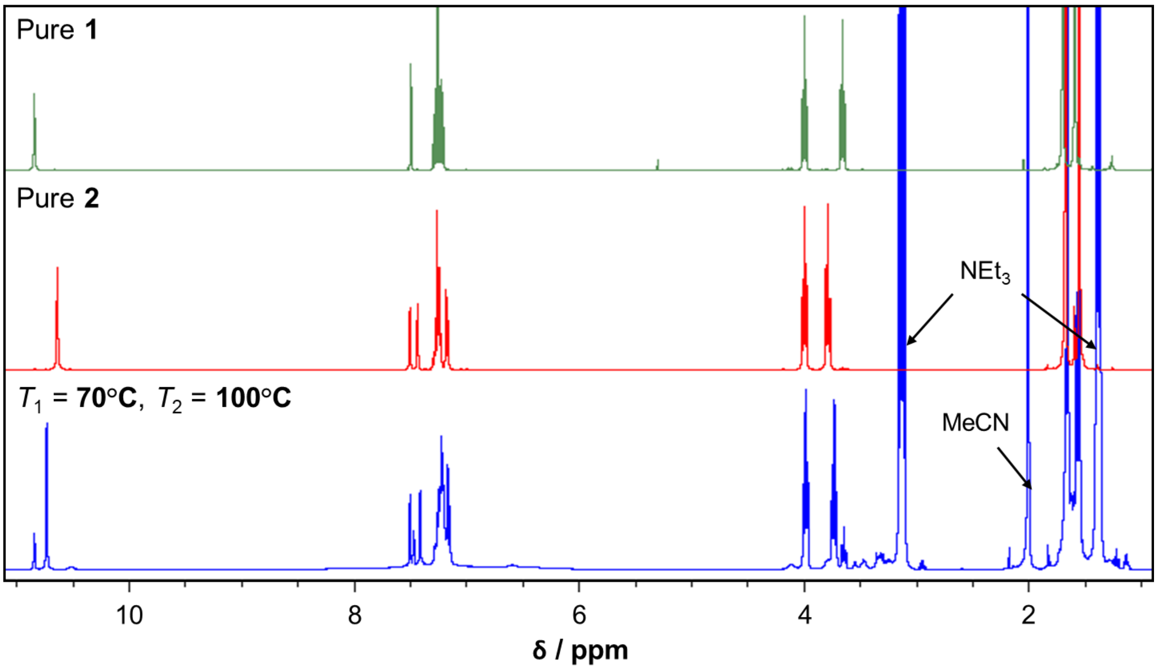

Fig. S42 ^{1}H NMR spectra of **1** and **2** and the crude product of a semi-continuous flow synthesis performed at T_1 = 70°C and T_2 = 100°C. All spectra were recorded in $CDCl_3$. The product of the flow reaction contains triethylamine and triethylammonium chloride and an acetonitrile standard for conversion measurements. However, all other major peaks can be assigned to **1** and **2**, suggesting near-complete formation of the target compounds. A total macrocycle conversion of 93% and selectivity of 80% were calculated for this reaction trial.

T_1 / °C	Conversion (total) / %	Conversion (2) / %	Selectivity / %
50	7.5	7.2	94.8
55	12.4	11.7	94.3
60	15.4	14.4	93.6
65	21.2	19.7	93.1
70	24.5	22.7	92.7
75	24.6	22.8	92.7
80	23.8	19.5	81.8
85	24.3	18.2	75.0
90	25.4	17.2	67.8
95	23.2	14.8	64.0
100	19.7	12.2	62.1

Table S11 Conversions and selectivities of semi-continuous flow syntheses performed at T_2 = 50°C. Values were obtained through ^{1}H NMR analysis of the crude products after quenching with methanol, removal of the solvent *in vacuo* and dissolution of the residue in $CDCl_3$. Selectivities for **2** were calculated from the relative integrals of the macrocycle NH signals, while conversions were estimated by comparison with the CH_3 integral of an acetonitrile internal standard. Where replicate experiments were performed, mean values are shown (see Table S12).

T_1 / °C	Sample	Conversion (total) / %	Conversion (2) / %	Selectivity / %
50	1	6.91	6.51	94.15
	2	8.18	7.81	95.54
	**Mean**	7.5 ± 0.6	7.2 ± 0.7	94.8 ± 0.7
65	1	20.84	19.33	92.80
	2	21.52	20.10	93.40
	**Mean**	21.2 ± 0.3	19.7 ± 0.4	93.1 ± 0.3
80	1	24.96	20.55	82.30
	2	22.63	18.40	81.31
	**Mean**	23.8 ± 1.2	19.5 ± 1.1	81.8 ± 0.5
90	1	26.19	17.87	68.24
	2	24.68	16.62	67.32
	**Mean**	25.4 ± 0.8	17.2 ± 0.6	67.8 ± 0.5
100	1	18.08	11.30	62.50
	2	21.29	13.12	61.61
	**Mean**	19.7 ± 1.6	12.2 ± 0.9	62.1 ± 0.4

Table S12 Replicate conversion and selectivity measurements for semi-continuous flow syntheses performed at T_2 = 50°C. Errors correspond to the separation of each pair of measurements from the mean. The replicates are reasonably concordant, exhibiting deviations from the mean of less than two percentage points.

T_2 / °C	Conversion (total) / %	Conversion (2) / %	Selectivity / %
50	23.7	22.4	94.7
55	27.0	25.5	94.4
60	31.1	28.6	92.0
65	44.4	40.5	91.2
70	61.1	54.2	88.7
75	72.2	62.9	87.2
80	80.8	69.3	85.7
85	82.8	68.7	83.0
90	84.9	69.8	82.2
95	88.9	72.8	81.9
100	92.7	74.2	80.0

Table S13 Conversions and selectivities of semi-continuous flow syntheses performed at T_1 = 70°C. Values were obtained through ^{1}H NMR analysis of the crude products after quenching with methanol, removal of the solvent *in vacuo* and dissolution of the residue in $CDCl_3$. Selectivities for **2** were calculated from the relative integrals of the macrocycle NH signals, while conversions were estimated by comparison with the CH_3 integral of an acetonitrile internal standard. Where replicate experiments were performed, mean values are shown (see Table S14).

T_2 / °C	Sample	Conversion (total) / %	Conversion (2) / %	Selectivity / %
55	1	25.49	24.13	94.68
	2	28.42	26.76	94.17
	**Mean**	27.0 ± 1.5	25.5 ± 1.3	94.4 ± 0.3
65	1	44.06	40.33	91.53
	2	44.71	40.64	90.91
	**Mean**	44.4 ± 0.3	40.5 ± 0.2	91.2 ± 0.3
75	1	74.00	64.07	86.58
	2	70.32	61.78	87.84
	**Mean**	72.2 ± 1.8	62.9 ± 1.1	87.2 ± 0.6
80	1	82.69	70.56	85.33
	2	78.97	67.96	86.05
	**Mean**	80.8 ± 1.9	69.3 ± 1.3	85.7 ± 0.4
90	1	84.73	69.88	82.48
	2	85.14	69.80	81.98
	**Mean**	84.9 ± 0.2	69.8 ± 0.1	82.2 ± 0.2
95	1	89.97	73.57	81.78
	2	87.82	72.02	82.02
	**Mean**	88.9 ± 1.1	72.8 ± 0.8	81.9 ± 0.1

Table S14 Replicate conversion and selectivity measurements for semi-continuous flow syntheses performed at T_1 = 70°C. Errors correspond to the separation of each pair of measurements from the mean. The replicates are reasonably concordant, exhibiting deviations from the mean of less than two percentage points.

Test	Batch Conversion (total) / %	Batch Conversion (**2**) / %	Batch Selectivity / %	Flow Conversion (total) / %	Flow Conversion (**2**) / %	Flow Selectivity / %
1	28.72	28.07	97.71	26.12	24.93	95.42
2	29.19	28.46	97.51	25.76	24.59	95.44
3	26.73	26.33	98.52	29.21	27.71	94.87
4	28.54	27.84	97.58	27.39	26.36	96.21
**Mean**	28.3 ± 1.1	27.7 ± 0.9	97.8 ± 0.5	27.1 ± 1.6	25.9 ± 1.4	95.5 ± 0.5

Table S15 Replicate conversion and selectivity measurements for batch and semi-continuous flow syntheses performed for equal durations at $T_1 = T_2 = 60°C$. To account for the time taken to reach steady-state conditions in flow, batch mixtures were left to stand at room temperature (21°C) for 30 minutes before being stirred at 60°C. Errors correspond to the standard deviations of the replicate experiments.

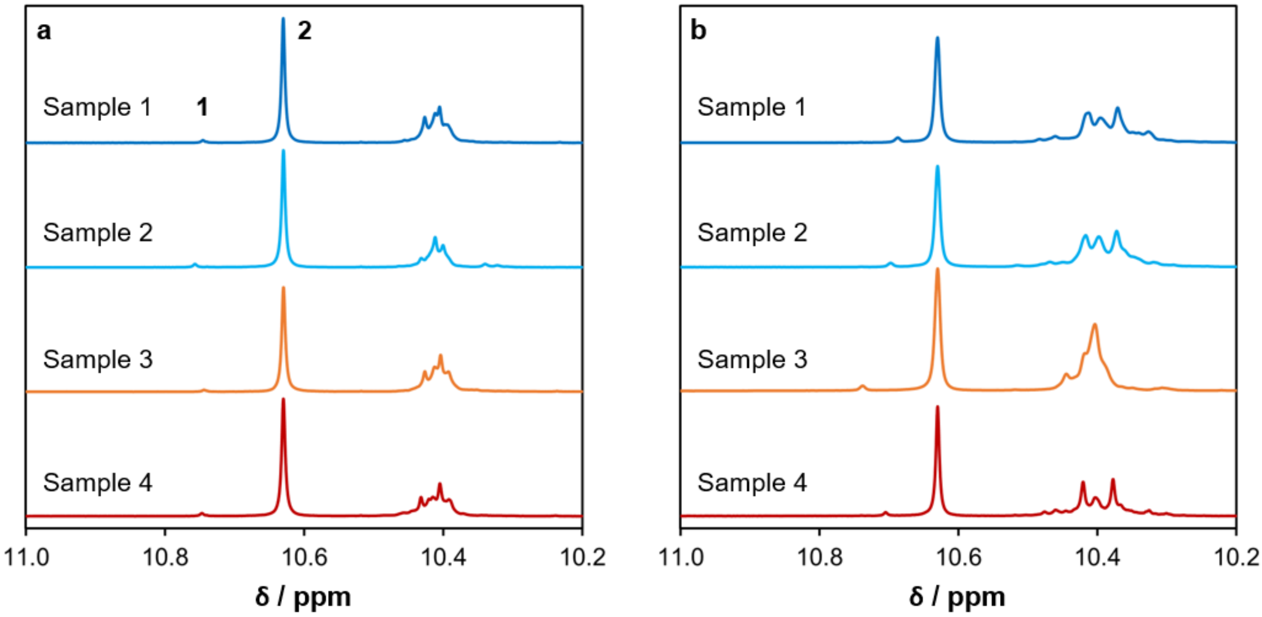

Fig. S43 ^{1}H NMR spectra of the crude products from replicate (a) batch and (b) semi-continuous flow syntheses at 60°C. The intensities of the macrocycle NH signals (labelled in bold) are scaled by concentration via normalization against an acetonitrile internal standard. For ease of comparison, the NH signal of **2** is shifted to a fixed position of δ = 10.63 ppm in all experiments. The two synthetic methods produce similar NH signals, indicating that there is little variation in their conversions or selectivities. Differences between the non-macrocycle NH signals around δ = 10.4 ppm suggest the use of a flow platform affects the formation of other products and intermediates, perhaps due mixing of reagents in flow before the second reaction step. These effects will be investigated further in future work.

9 Host-guest binding studies

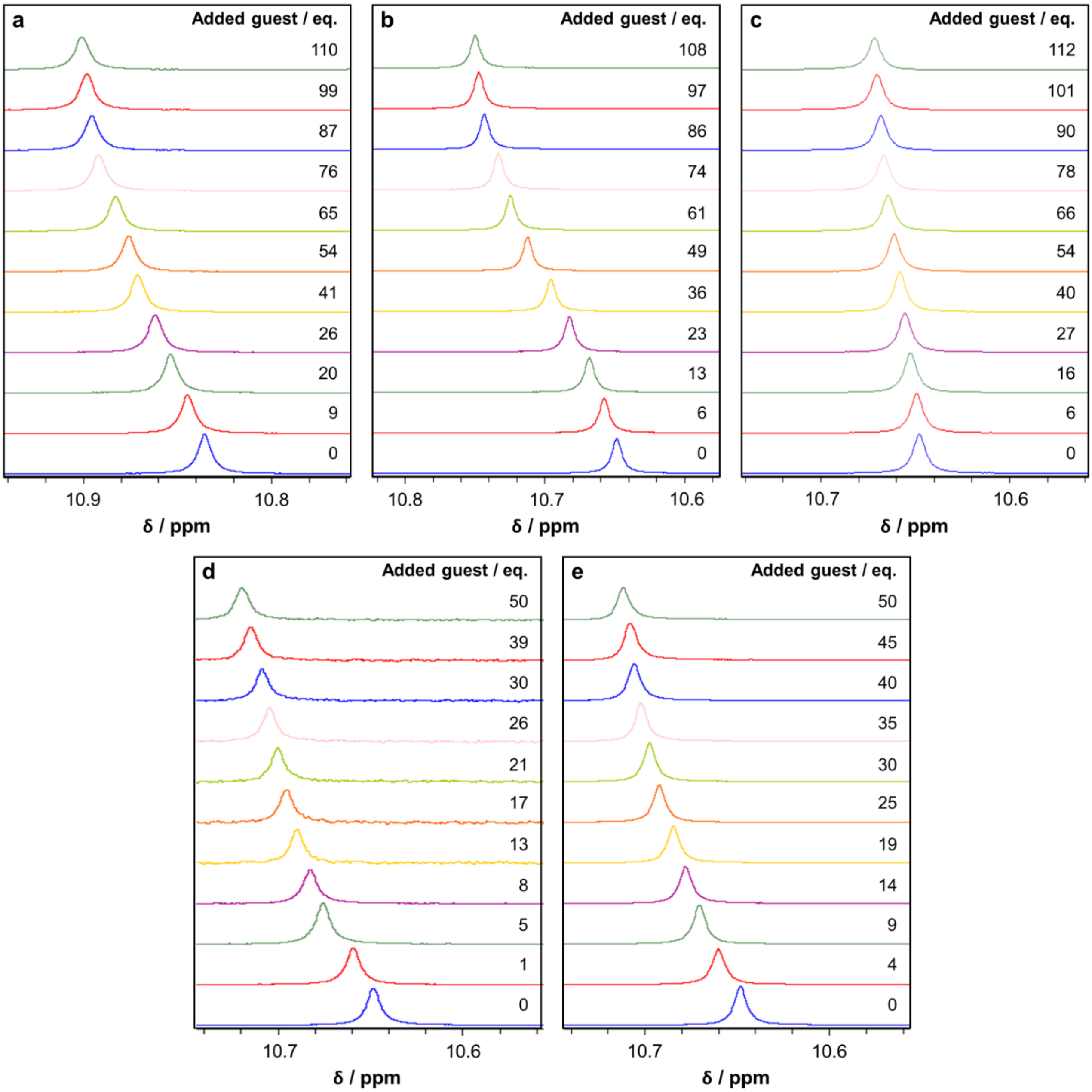

Fig. S44 ^{1}H NMR spectra showing the macrocycle NH signals in solutions of (a) **1** and methanol, (b) **2** and methanol, (c) **2** and acetonitrile, (d) **2** and TBAF trihydrate and (e) **2** and anhydrous TBACl. All spectra were recorded in $CDCl_3$ with TMS (0.05% w/v) as an internal reference (δ = 0.0 ppm).

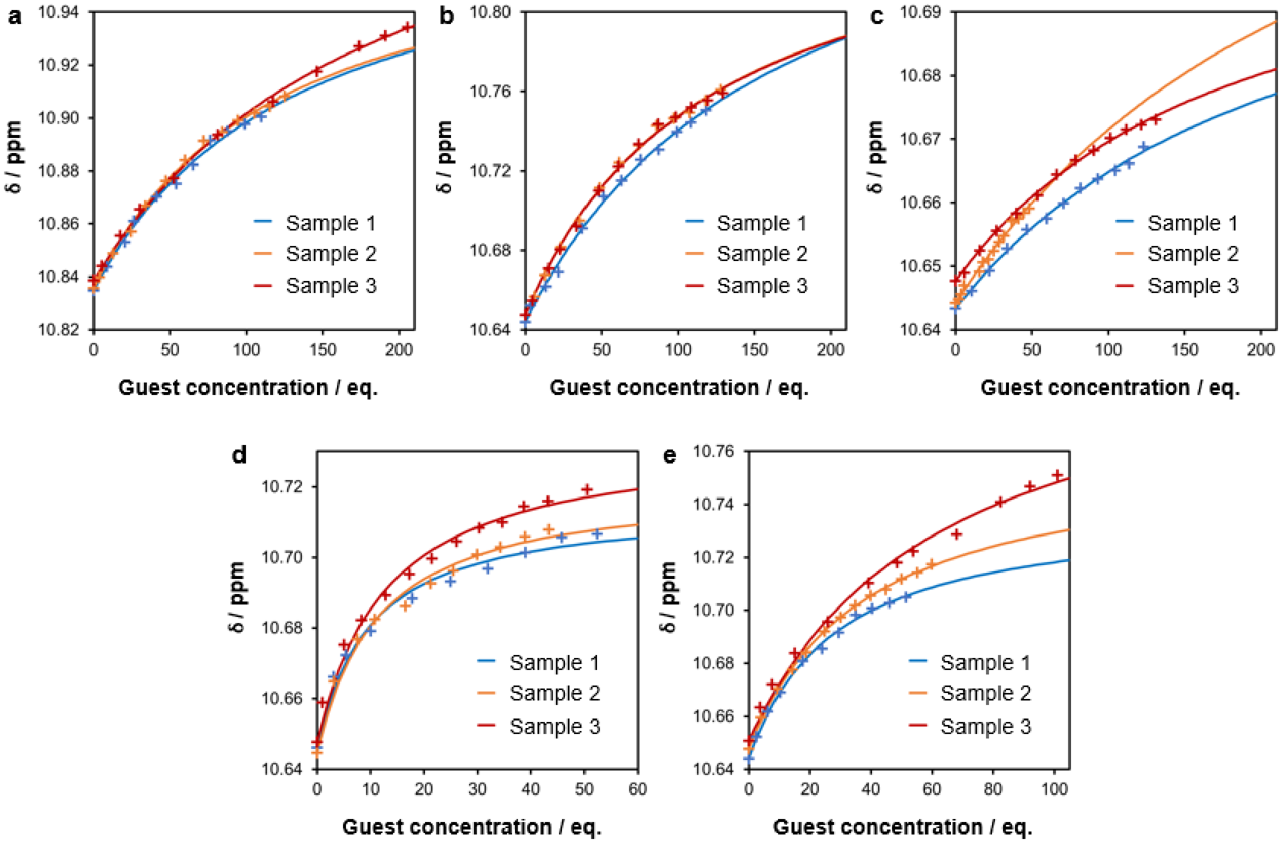

Fig. S45 ^{1}H chemical shifts of macrocycle NH peaks in $CDCl_3$ solutions of (a) **1** and methanol, (b) **2** and methanol, (c) **2** and acetonitrile, (d) **2** and TBAF trihydrate and (e) **2** and anhydrous TBACl. The titration data were obtained by varying the guest concentration at a fixed macrocycle concentration of 9.0 mM, with TMS (0.05% w/v) as an internal reference (δ = 0.0 ppm). Trend lines illustrate 1:1 binding isotherms fitted to the data via a Nelder-Mead algorithm in the online software BindFit.[4, 5] For some host-guest combinations, different guest concentration ranges were used to assess the effect of the experimental design on the consistency of the fitted binding models. Divergence of replicate plots at high guest concentrations is likely attributable to small differences in sample preparation and has little effect on the fitted K_{11} values (Table S16).

**Host**	**Guest**	**K_{11} / M^{-1}**			**Mean / M^{-1}**	**Host bound by 50 eq. guest / %**
		Trial 1	Trial 2	Trial 3		
**1**	MeOH	0.841 ± 0.035	0.929 ± 0.026	0.585 ± 0.016	0.78 ± 0.13	26 ± 3
**2**	MeOH	0.686 ± 0.022	0.913 ± 0.026	0.908 ± 0.027	0.84 ± 0.09	27 ± 2
	MeCN	0.498 ± 0.013	0.584 ± 0.013	0.434 ± 0.014	0.51 ± 0.05	18 ± 2
	TBAF	11.9 ± 2.0	10.5 ± 1.2	8.9 ± 0.8	10.4 ± 1.0	90 ± 1
	TBACl	3.99 ± 0.16	2.97 ± 0.05	1.79 ± 0.11	2.9 ± 0.8	56 ± 7

Table S16 Binding constants for host-guest complexes of **1** and **2**, measured from ^{1}H NMR spectra of 9.0 mM solutions of the macrocycles in $CDCl_3$ with varying concentrations of the added guest. TMS (0.05% w/v) was included as an internal reference (δ = 0.0 ppm). Data were fitted to 1:1 binding isotherms using a Nelder-Mead algorithm in the online software BindFit.[4, 5] Errors in the individual K_{11} measurements correspond to uncertainty in the fits, while standard errors are shown for the mean values.

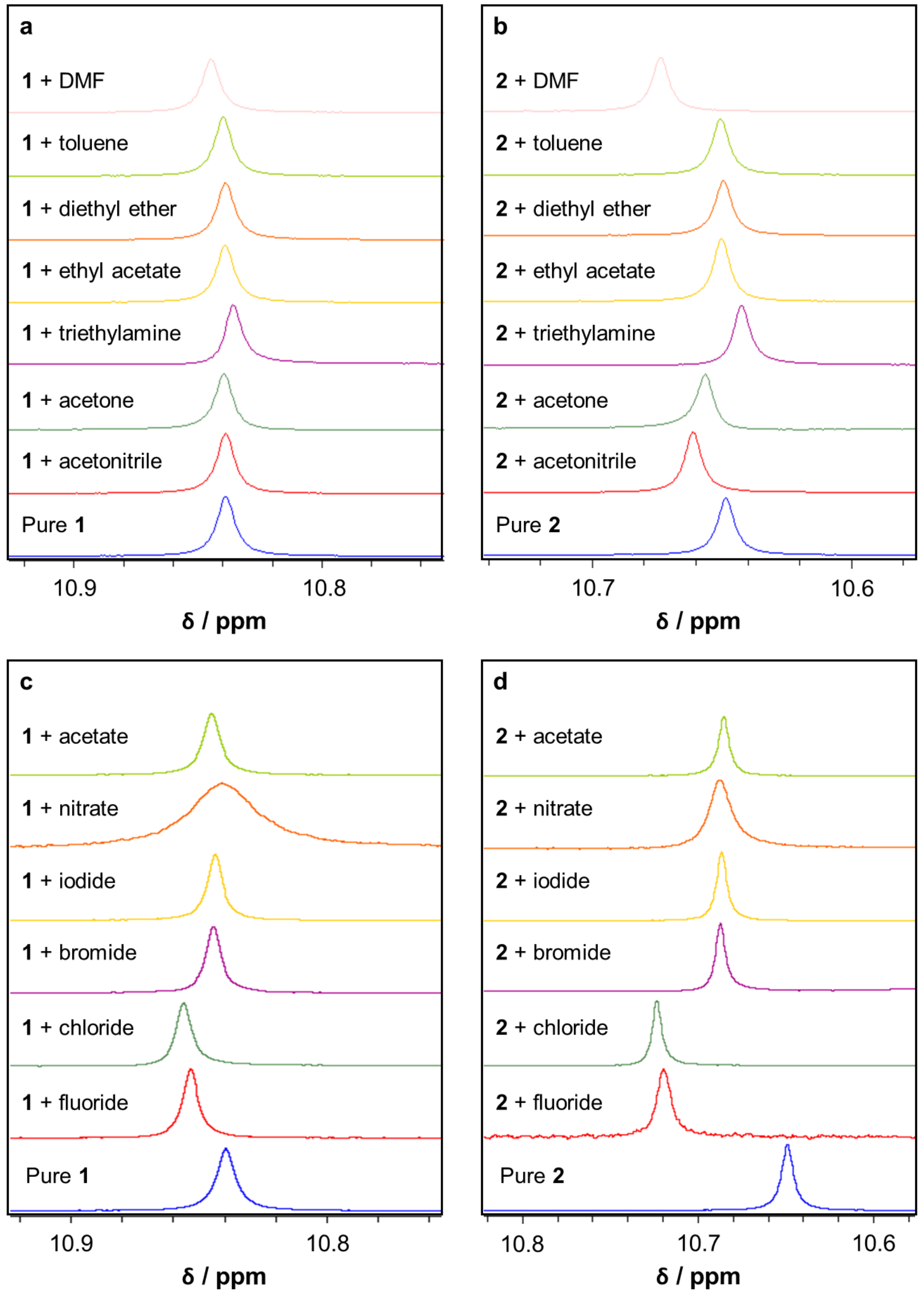

Fig. S46 ^{1}H NMR spectra illustrating changes in the NH signals of (a) **1** with neutral guests, (b) **2** with neutral guests, (c) **1** with ionic guests and (d) **2** with ionic guests. In all experiments, 50 eq. of guest were added to a 9.0 mM macrocycle solution in $CDCl_3$. Ionic guests were added as TBA salts and TMS (0.05% w/v) was used as an internal reference (δ = 0.0 ppm).

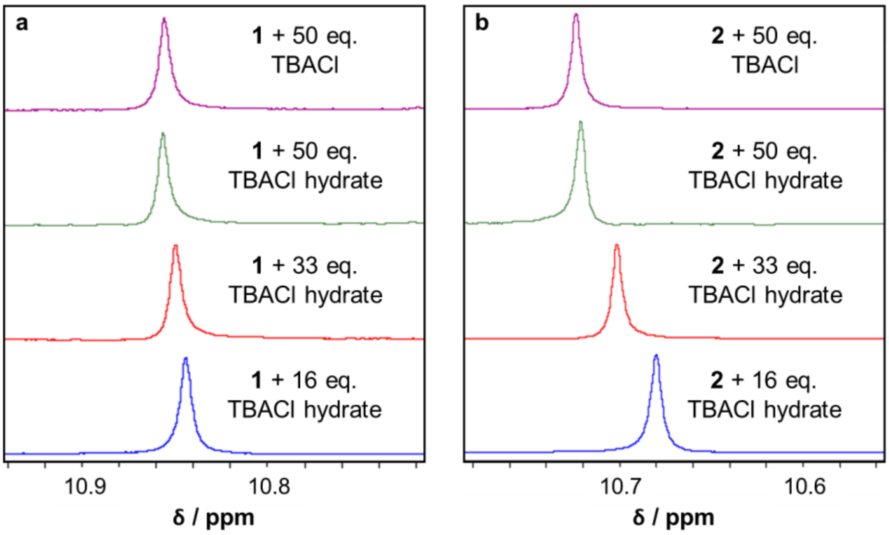

Fig. S47 ^{1}H NMR spectra illustrating changes in the NH signals of **1** and **2** (9.0 mM) in $CDCl_3$ with varying concentrations of hydrated and anhydrous TBACl. Equivalents of TBACl hydrate were estimated assuming a water-chloride ratio of 3:1, and TMS (0.05% w/v) was used as an internal reference (δ = 0.0 ppm). The hydrated and anhydrous salts produce similar changes in the NH chemical shifts, suggesting that water of crystallization has little effect on host-guest binding. The weak influence of water relative to other hydrogen bond donors such as methanol may be attributable to the immiscibility of the guest with $CDCl_3$.

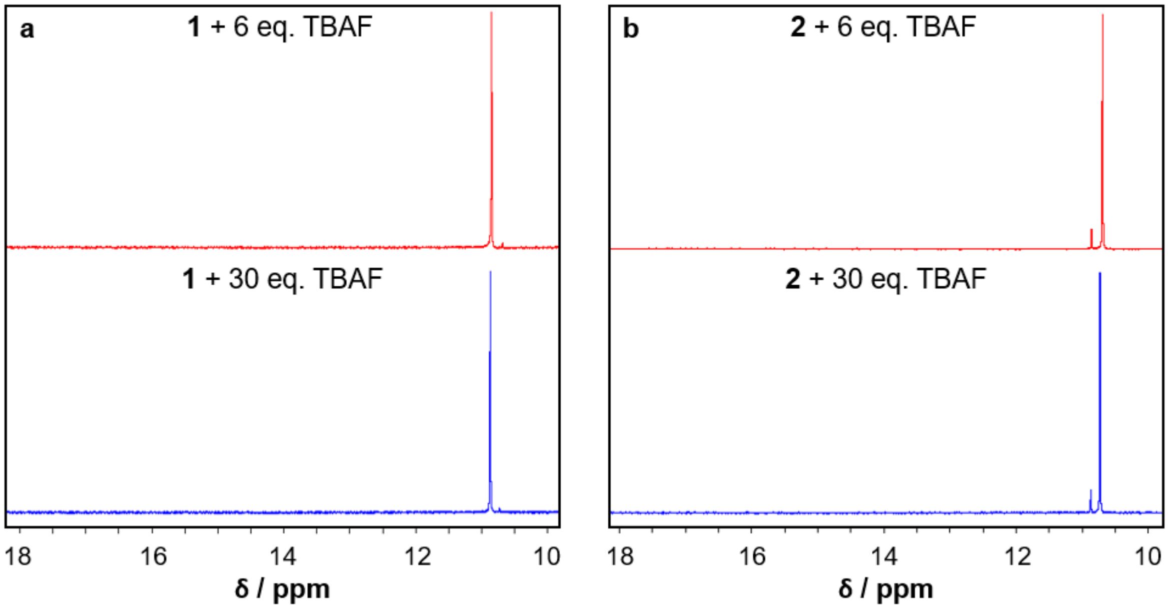

Fig. S48 ^{1}H NMR spectra of (a) **1** and (b) **2** with different concentrations of TBAF trihydrate in $CDCl_3$. To increase the signal-to-noise ratio of potential product signals, elevated macrocycle concentrations of 17 mM were used in all experiments. No signals are visible in the region 15-17 ppm, suggesting that the macrocycles are not deprotonated by fluoride to form the bifluoride (HF_2^-) ion.[16]

10 Binding energy calculations

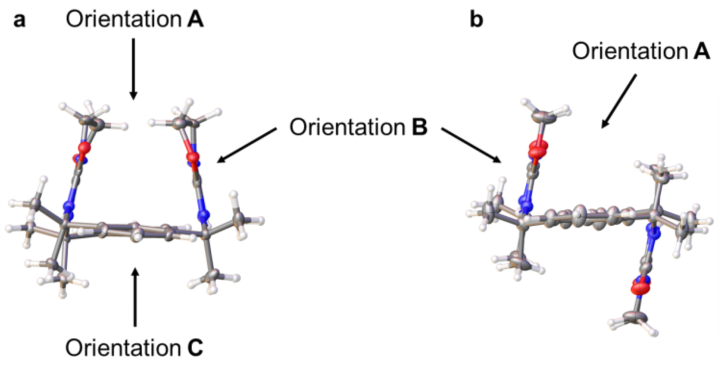

Fig. S49 Possible orientations of guests relative to macrocycle **1** in the (a) *syn* and (b) *anti* conformations. Host-guest interaction energy (E_{int}) values were calculated using manually constructed model systems of **1** and **2** with all of the guest orientations shown. In some cases, multiple models with similar guest orientations were used, in order to assess the relative stabilities of different packing modes, dipole-dipole interactions and hydrogen bond donor-acceptor motifs. These orientations were not always preserved during the DFT geometry optimizations.

**Guest**	**E_{int} / kJ mol^{-1}**									
	***syn*-1** *Orientation*			***anti*-1** *Orientation*		***syn*-2** *Orientation*			***anti*-2** *Orientation*	
	A	B	C	A	B	A	B	C	A	B
Chloroform	**-52.7**	-34.9	-38.9	**-23.7**	-23.6	-24.2	-6.8	-21.0	**-23.9**	-23.9
	-52.7			-23.6		**-37.4**			-10.8	
Acetone	**-48.3**	-33.4	-35.6	**-19.7**	-11.9	-28.4	-28.3	-17.7	**-20.4**	-11.6
	-35.6	-48.3		-18.4		**-44.6**	-2.1		-10.8	
Acetonitrile	**-50.7**	-32.4	-35.2	**-20.9**	-2.1	-33.7	-33.7	-16.6	**-21.6**	-21.3
	-42.7			-12.5		**-52.1**			-12.4	
Methanol	**-66.7**	-9.8	-41.8	**-32.6**	-13.8	-38.9	-10.3	-17.4	**-32.8**	-32.7
	-66.7	-11.7		-26.9	-13.9	**-54.9**	-12.6		-32.8	-14.0
	-66.6	-11.7		-32.6	-9.8	-38.8	-12.6		-32.8	-14.0
Fluoride	-173.3	-134.7	-187.3	**-149.9**	-128.0	**-181.3**	-138.8	-166.9	**-158.9**	-128.4
	-170.9			-128.0		-181.3			-158.9	
	**-187.3**					-132.3				
	-147.5					-166.9				
Chloride	**-110.4**	-62.0	-94.2	**-69.3**	-48.1	**-111.8**	-54.8	-78.1	**-69.1**	-49.9
	-100.0			-69.3		-98.2			-69.1	
Nitrate	-95.6	-76.7	-68.2	**-67.1**	-59.4	-92.1	-77.6	-69.1	**-67.5**	-59.5
	**-106.2**			-67.1		**-93.8**			-67.5	

Table S17 Calculated interaction energy (E_{int}) values for complexes of **1** and **2** with a variety of guests. Each complex was optimized from a range of estimated initial configurations with different relative orientations of the host and guest (see Fig. S49), using the DFT method B3LYP in the basis set 6-31+G*. The output structures were further optimized in the larger basis set 6-31++G**, and the E_{int} values calculated by subtracting the energies of the separate macrocycles and guest species. For ease of comparison, relative E_{int} values were determined (Figs. S50 and S51 and Fig. 15, main article) from the absolute E_{int} values shown in this table by subtracting the energy of the most stable chloroform complex from the most negative E_{int} value of each complex (highlighted in bold).

Guest	BSSE / kJ mol^{-1}
Chloroform	0.093<br>0.079
Acetone	0.070<br>0.062
Acetonitrile	0.052<br>0.024
Methanol	0.206<br>0.116<br>0.221
Fluoride	0.034<br>0.035<br>0.014<br>0.010
Chloride	0.006<br>0.063
Nitrate	0.130<br>0.095

Table S18 Basis set superposition error (BSSE) values for DFT optimizations of *syn*-**2** in complexes with various guests. All guests were bound between the oxazolidine rings in the starting structures (orientation A) and optimized with the B3LYP method in the basis set 6-31+G*, followed by refinement in the larger basis set 6-31++G**. BSSE values were calculated by performing the final optimization step with and without counterpoise corrections and calculating the difference between the converged energy values. Energies calculated without counterpoise corrections were more negative in all cases, indicating that BSSE increases the apparent stabilities of the host-guest complexes. However, BSSE accounts for a negligible proportion (<0.6%) of the E_{int} values.

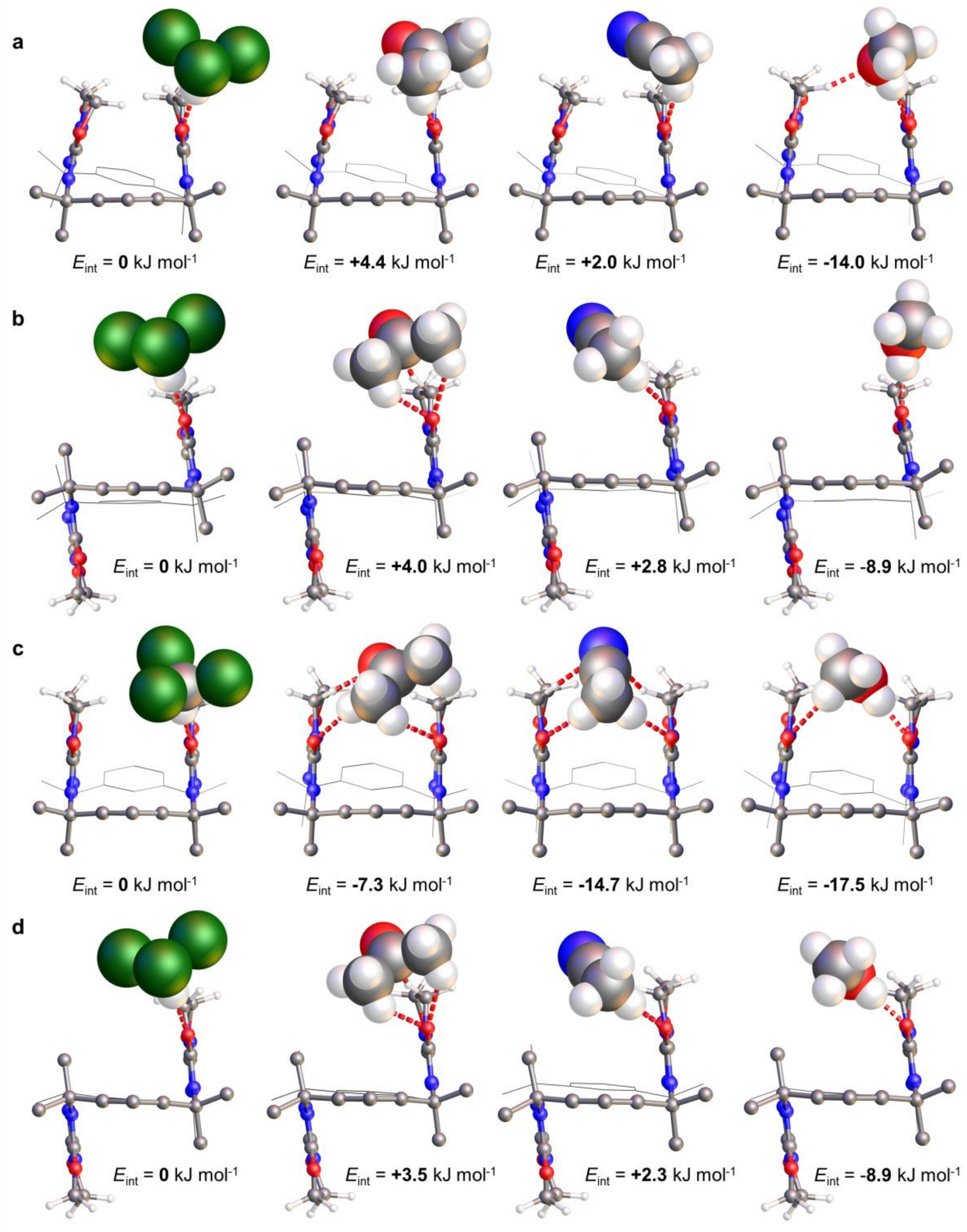

Fig. S50 Most stable calculated host-guest complexes of chloroform, acetone, acetonitrile and methanol with (a) *syn*-**1**, (b) *anti*-**1**, (c) *syn*-**2** and (d) *anti*-**2**. Geometry optimizations were performed using the DFT method B3LYP in the basis set 6-31+G*, followed by refinement in the larger basis set 6-31++G**. Close contacts (<2.7 Å) between hydrogen bond donor and acceptor sites are marked with red dashed lines, and parts of the macrocycles are omitted for clarity. In all cases, the most stable complexes are formed by guests in orientation A (Fig. S49). E_{int} values are expressed relative to the corresponding chloroform complexes.

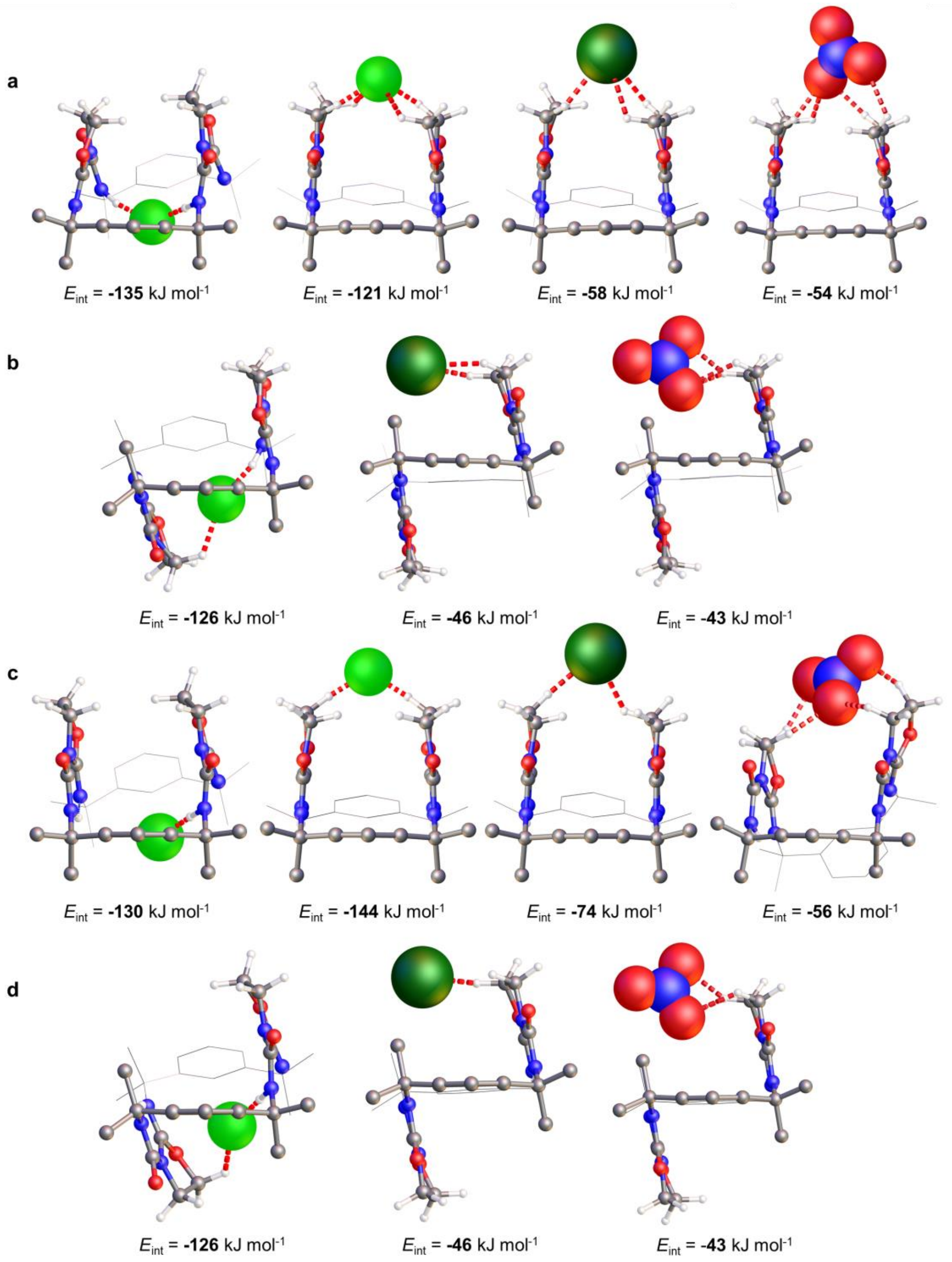

Fig. S51 Most stable calculated host-guest complexes of fluoride, chloride and nitrate with (a) *syn*-**1**, (b) *anti*-**1**, (c) *syn*-**2** and (d) *anti*-**2**. Where NH$\cdots$F$^-$ and CH$\cdots$F$^-$ motifs are similarly stable, both complexes are shown. Geometry optimizations were performed using the DFT method B3LYP in the basis set 6-31+G*, followed by refinement in the larger basis set 6-31++G**. Close contacts (<2.7 Å) between ions and hydrogen atoms are marked with red dashed lines, and parts of the macrocycles are omitted for clarity. In all cases, the most stable complexes are formed by ions in orientation A (Fig. S49). E_{int} values are expressed relative to the corresponding chloroform complexes.

11 References

1. Dolomanov, O. V.; Bourhis, L. J.; Gildea, R. J.; Howard, J. A. K.; Puschmann, H., OLEX2: a complete structure solution, refinement and analysis program. *J. Appl. Crystallogr.* **2009,** *42* (2), 339-341.
2. Sheldrick, G., SHELXT - Integrated space-group and crystal-structure determination. *Acta Cryst. A* **2015,** *71* (1), 3-8.
3. Sheldrick, G., Crystal structure refinement with SHELXL. *Acta Cryst. C* **2015,** *71* (1), 3-8.
4. http://supramolecular.org (accessed December 2020).
5. Brynn Hibbert, D.; Thordarson, P., The death of the Job plot, transparency, open science and online tools, uncertainty estimation methods and other developments in supramolecular chemistry data analysis. *Chem. Commun.* **2016,** *52* (87), 12792-12805.
6. Frisch, M. J.; Trucks, G. W.; Schlegel, H. B.; Scuseria, G. E.; Robb, M. A.; Cheeseman, J. R.; Scalmani, G.; Barone, V.; Petersson, G. A.; Nakatsuji, H.; Li, X.; Caricato, M.; Marenich, A. V.; Bloino, J.; Janesko, B. G.; Gomperts, R.; Mennucci, B.; Hratchian, H. P.; Ortiz, J. V.; Izmaylov, A. F.; Sonnenberg, J. L.; Williams; Ding, F.; Lipparini, F.; Egidi, F.; Goings, J.; Peng, B.; Petrone, A.; Henderson, T.; Ranasinghe, D.; Zakrzewski, V. G.; Gao, J.; Rega, N.; Zheng, G.; Liang, W.; Hada, M.; Ehara, M.; Toyota, K.; Fukuda, R.; Hasegawa, J.; Ishida, M.; Nakajima, T.; Honda, Y.; Kitao, O.; Nakai, H.; Vreven, T.; Throssell, K.; Montgomery Jr., J. A.; Peralta, J. E.; Ogliaro, F.; Bearpark, M. J.; Heyd, J. J.; Brothers, E. N.; Kudin, K. N.; Staroverov, V. N.; Keith, T. A.; Kobayashi, R.; Normand, J.; Raghavachari, K.; Rendell, A. P.; Burant, J. C.; Iyengar, S. S.; Tomasi, J.; Cossi, M.; Millam, J. M.; Klene, M.; Adamo, C.; Cammi, R.; Ochterski, J. W.; Martin, R. L.; Morokuma, K.; Farkas, O.; Foresman, J. B.; Fox, D. J. *Gaussian 16 Rev. C.01*, Wallingford, CT, 2016.
7. Becke, A. D., A new mixing of Hartree–Fock and local density-functional theories. *J. Chem. Phys.* **1993,** *98* (2), 1372-1377.
8. Frisch, M. J.; Pople, J. A.; Binkley, J. S., Self-consistent molecular orbital methods 25. Supplementary functions for Gaussian basis sets. *J. Chem. Phys.* **1984,** *80* (7), 3265-3269.
9. Weigend, F.; Ahlrichs, R., Balanced basis sets of split valence, triple zeta valence and quadruple zeta valence quality for H to Rn: Design and assessment of accuracy. *Phys. Chem. Chem. Phys.* **2005,** *7* (18), 3297-3305.
10. Woon, D. E.; Dunning, T. H., Gaussian basis sets for use in correlated molecular calculations. III. The atoms aluminum through argon. *J. Chem. Phys.* **1993,** *98* (2), 1358-1371.
11. Grimme, S.; Ehrlich, S.; Goerigk, L., Effect of the damping function in dispersion corrected density functional theory. *J. Comput. Chem.* **2011,** *32* (7), 1456-1465.
12. van Duijneveldt, F. B.; van Duijneveldt-van de Rijdt, J. G. C. M.; van Lenthe, J. H., State of the Art in Counterpoise Theory. *Chem. Rev.* **1994,** *94* (7), 1873-1885.
13. Jones, C. D.; Kennedy, S. R.; Walker, M.; Yufit, D. S.; Steed, J. W., Scrolling of Supramolecular Lamellae in the Hierarchical Self-Assembly of Fibrous Gels. *Chem* **2017,** *3* (4), 603-628.
14. Remko, M.; Walsh, O. A.; Richards, W. G., Ab initio and DFT study of molecular structure and tautomerism of 2-amino-2-imidazoline, 2-amino-2-oxazoline and 2-amino-2-thiazoline. *Chem. Phys. Lett.* **2001,** *336* (1), 156-162.
15. Gowenlock, B. G., Arrhenius factors (frequency factors) in unimolecular reactions. *Q. Rev. Chem. Soc.* **1960,** *14* (2), 133-145.
16. Montis, R.; Bencini, A.; Coles, S. J.; Conti, L.; Fusaro, L.; Gale, P. A.; Giorgi, C.; Horton, P. N.; Lippolis, V.; Mapp, L. K.; Caltagirone, C., Fluoride binding by an anionic receptor: tuning the acidity of amide NH groups for basic anion hydrogen bonding and recognition. *Chem. Commun.* **2019,** *55* (19), 2745-2748.
